# Supplementary material for: Protocol for a cluster randomised placebo-controlled trial of adjunctive ivermectin mass drug administration for malaria control on the Bijagós Archipelago of Guinea-Bissau: the MATAMAL trial
Source: BMJ Open. 2023 Jul 7;13(7):e072347. doi: 10.1136/bmjopen-2023-072347 (PMC10335573; doi:10.1136/bmjopen-2023-072347)
Supplement: Supplementary data [file bmjopen-2023-072347supp001.pdf]

MATAMAL v2.0 20/04/2020

PARTICIPANT INFORMATION SHEET (ADULT)

Study Title:

MATAMAL

Ivermectin Mass Drug Administration for Malaria Control on the Bijagós Archipelago of Guinea Bissau: A cluster randomised placebo-controlled trial

Sponsor & Funder: The Joint Global Health Trials Scheme (DFID / NIHR / MRC / Wellcome Trust) is funding the study and LSHTM is the sponsor.

What is informed consent?

You are invited to take part in a research study. Before you decide you need to understand why the research study is being done and what it will involve. Please take time to read the following information or get the information explained to you in your language. Listen carefully and feel free to ask if there is anything that is not clear or you do not understand. You may also wish to consult your spouse, family members, friends or others before deciding to take part in the study.

If you decide to join the study, you will need to sign or put a thumbprint on a consent form saying that you agree to be in the study.

Why is this study being done?

This study is being done to assess the impact of a new approach to prevent malaria by using two different medicines, which will be given to everyone on the islands. As you may know, malaria in several African countries has declined substantially over the past few years, because of control measures like bed nets and use of medicines to prevent people from getting malaria or to treat people when they become ill with malaria. Even though many people are using these interventions, people on the island still get sick with malaria. To prevent malaria, we need to find extra control interventions. One of these interventions could be mass drug administration, which means giving medicines (tablets) to everyone from every community on the islands. We are going to use two medicines. One is called dihydroartemisinin-piperaquine (DP). This is an antimalarial drug that will kill the parasite that causes malaria if it is in your blood. This has worked well to reduce malaria in other countries in West Africa. The other drug is called ivermectin (IVM). When a person takes IVM, their blood can kill the mosquitoes that transmit malaria when they bite you. IVM is a drug that is also used to kill other parasites that infect people (such as some intestinal worms, headlice and bedbugs). We want to see if giving treatment to the whole community with these two drugs is better than just giving the whole community DP at reducing malaria on the islands. We also want to find out whether giving IVM can treat other parasites in addition to reducing malaria.

What does this study involve?

We are working in all of the villages on all the islands in the archipelago. We have done previous surveys on the islands to measure the proportion of people with malaria infection. All the islands will be included in this study. People from every island will be asked to participate in the study and will be given the two medicines described above. Half the islands will be given a placebo (‘sugar’ or ‘dummy’) medicine instead of ivermectin. The placebo medicine does not contain any drug of any kind. It looks exactly the same as the ‘real’ ivermectin medicine. The islands that are given the placebo medicine have been chosen randomly (by chance). Everyone will receive medication that looks exactly the same. Neither the study team nor you will know which medicine you will receive (placebo or ivermectin). Dihydroartemesinin-piperaquine will be given to everyone. The reason that we are using a placebo medicine is so that we can see whether it is better to ivermectin MDA with DP MDA or whether DP alone is good enough. All medicines will be given according to body weight or height. The medicine will be given to you once each day (by mouth with water and without food) for three days each month in June, July and August every year for two years. The medicine should be taken at least three hours after you last ate food and no food should be eaten for three hours after each dose. If you are unable to swallow the tablets, the medicines may be crushed and mixed with water. The mixture will be used immediately after preparation. If you vomit within 30 minutes of taking the medicine, the whole dose will be given again, but if vomiting occurs after 30-60 minutes, only half the dose will be given again. All the treatment will be directly supervised by our study team. All the islands will also receive the malaria programme control measures provided by the Bissau-Guinean Ministry of Public Health (bed nets, preventative treatment in pregnancy and antimalarial treatment if anyone becomes unwell with malaria).

We will conduct surveys for the presence of malaria and other diseases in your communities. Participants in these surveys will be examined by trained health care members of the study research team. We will conduct one survey each year after the rainy season in October/November. We will conduct an additional survey for other diseases such as scabies and worms in February. During the surveys, a questionnaire will be used to collect information on whether you slept under a bed net the previous night. You will also be asked whether you have travelled recently. Questions will also be asked about how and whether you took the medicines given during the mass drug administration. In addition, several droplets of blood will be taken from your finger onto a filter paper to see if there are parasites in his/her blood. This test will be done later in the MRC laboratory in The Gambia. However, if you have fever or recent history of fever, a malaria rapid diagnostic test will be done at the time to check if he/she is sick with malaria. If this is positive, he/she will receive treatment for malaria at that time.

During the course of the study, you will be encouraged to visit your nearest health post or talk to the ASC responsible for your tabanca if you feel sick with a fever. At the health facility a finger prick will be done for a malaria rapid diagnostic test. If the rapid diagnostic test is positive,

additional droplets of blood will be collected onto a filter paper and slide for malaria tests in the MRC laboratory in The Gambia and you will be treated with artemether-lumefantrine according to national guidelines.

Your house may be selected to house a special light trap to capture mosquitoes for six nights each month during the intervention and surveys. This will be like having a low level lamp on in your house during the night.

The following three paragraphs of information are relevant to you if you have children, for whom you will be asked to provide consent on their behalf on a separate form.

*****Most cases of malaria happen in children between the ages of five to fourteen years. During the rainy season we will randomly select 50 children from each island in this age group and our study team will visit them each month until the end of the transmission season (six months from June-November). Your child may be selected to participate in this cohort (group). The team will complete a short questionnaire, take your child's temperature and take a finger prick blood sample to collect several droplets of blood onto a filter paper to see if there are parasites in his/her blood. This test will be done later in the MRC laboratory in The Gambia. However, if your child has a fever or recent history of fever, a malaria rapid diagnostic test will be done at the time to check if he/she is sick with malaria. If this is positive, he/she will receive treatment for malaria at that time and a blood film will be taken to look for parasites under the microscope.

*****The medicine ivermectin also has additional benefits because it also treats common infestations and worms that we see in children. To see its effect on these infections, we will randomly select 100 children from each island, between the ages of four to thirteen years, the age group at highest risk of these infections. Your child may be selected for this assessment and will participate in a survey that will be done to estimate the prevalence of these common infections including parasites that affect the external body surface, scabies and worms. Bedbugs will be detected by visual inspection of the beds for 10 minutes using torchlight. Bed nets, mattresses (if present), bed frames and the wall next to the bed will be searched for the presence of eggs, immature and adult bedbugs. Head lice will be detected by a visual search of the scalp. The field assistant will sit with a white cloth across her/his lap and search the child's scalp before and after combing with a fine-toothed white nit comb. The comb and sheet will be inspected for the presence of lice and will take about 10 minutes to complete. Hair length will also be recorded. Scabies will be identified from a complete physical examination of the body and additionally, lesions will be examined for secondary infection. Skin swabs may be taken if there is any evidence of secondary skin infection. The examination will take approximately 10 minutes to complete. A stool sample will be taken to identify the presence of worms. All of the lab tests will be done at the MRC laboratory in The Gambia up to several months after the survey.

*If our study team find that your child has an infection that needs treatment at that time, our clinical team will dispense treatment or arrange for treatment at your health post. If our study team discovers that your child is sick and decides that your child cannot participate in the study because of that, your child will receive immediate care at the study site and then be referred to the appropriate health facility.

If the research study needs to be stopped for any reason, you will be informed and you and/or your child will have normal medical care.

What will happen to the samples taken in this study?

The blood samples will be used to determine whether malaria parasites are present in your blood. In addition, we will do tests to study how your body reacts to malaria parasites (immune tests) that will tell us whether you have had recent or past malaria exposure. These tests will be done in the MRC laboratory The Gambia but for quality control a selection of samples may be sent abroad to collaborating laboratories. Samples are labelled with unique identifying labels, so the laboratory technicians will not have any personal information about the sample. Stool samples will be used to test for the presence of worms. Additional stool tests for immune responses may be sent abroad to collaborators. Additional tests for infection and resistance to the medicines that we are using in this study may be carried out on stored blood samples from this study.

What harm or discomfort can you expect in the study?

All risks associated with the trial are low or moderate. You may experience discomfort from the finger prick blood samples we are taking. Sterile equipment will be used to take these samples. The medications used in this study are safe for use in this setting. Individuals to be treated will be advised to take the medicines as per manufacturer's instructions. Women in the first trimester of pregnancy or infants under 12 months of age will not be given dihydroartemisinin-piperaquine. Children less than 90 cm in height (or weighing less than 15kg), pregnant or breastfeeding women will not be treated with ivermectin or placebo. All participants will be monitored for any possible adverse events that may occur during the trial.

What benefits can you expect in the study?

This is the first large trial investigating the impact of ivermectin MDA with dihydroartemisinin-piperaquine MDA on malaria. Your participation in this trial is providing essential information to researchers and health care programmes so that we can establish whether ivermectin MDA will be successful in reducing malaria in your communities. If it does, ivermectin MDA might be able to accelerate the path towards malaria elimination on the Bijagos Islands and in other African countries where malaria is a big problem.

Will you be compensated for your participation in the study?

You will not get paid for your participation. In case the study clinician feels it is in your interest to visit a health facility, you will either get transport provided by the study team or get the costs for the transport reimbursed.

MATAMAL v2.0 20/04/2020

A clinical assessment and simple treatment may be provided by the study team in the field before referral.

What happens if you decide not to participate in the study or change your mind later?

You are free to decide whether to participate or not in the study and you have the right to stop participating at any time without giving a reason. This will not affect the medical care that you would normally receive. In case you decide to withdraw your participation during the study, any information already generated from the samples until the time of withdrawal will be used and samples already collected, for which you have given consent, will also be analysed and data used. The study doctor may also ask for tests for your safety.

Should any new information become available during the study that may affect your participation, you will be informed as soon as possible.

What compensation will be available if you are injured during the study?

We will be responsible for the provision of treatment for injuries caused by procedures related to the research study. If medical treatment is required as an emergency, please go directly to your health centre or clinic and contact the field assistant who gave his/her telephone number to you or contact Mrs Eunice Teixeira e Silva Cassama on +245 96 6288282. In addition, LSHTM carries Clinical Trial/Non-Negligent Harm Insurance and Medical Malpractice Insurance applicable to this study.

How will your information be kept and who will be allowed to see it?

All information that is collected about you in the course of the study will be kept strictly confidential. Your personal information will only be available to the study team members and might be seen by some rightful persons from the Ethics Committees, the sponsor and for confidential external monitoring purposes.

At the end of the project, the study data will be archived in London. The data will be made available to other researchers worldwide for research and to improve medical knowledge and patient care. Your personal information will not be included and there is no way that you can be identified, as these data will have been de-identified, or 'de-personalised', meaning that we have removed any personal identifiers, before making the data available for use in the circumstances above.

Who should you contact if you have questions?

If you have any queries or concerns you can contact Dr Harry Hutchins on +245 96 6871504 or Mrs Eunice Teixeira e Silva Cassama on +245 96 6288282 and you can always call the mobile phone numbers of the study staff that are given to you.

Please feel free to ask any question you might have about the research study.

Who has reviewed this study?

This study has been reviewed and approved by the Comite de Eticas na Saude in Bissau and the Ethics Committee at the London School of Hygiene and Tropical Medicine (UK). It has also been reviewed by a panel of scientists at the Medical Research Council The Gambia Unit. These committees review such studies to protect your rights and wellbeing before approving them.

Thank you for taking time to read this information leaflet. If you agree to take part in the study please read and sign the consent form in the presence of our researcher.
