# Supplementary material for: Protocol for a cluster randomised placebo-controlled trial of adjunctive ivermectin mass drug administration for malaria control on the Bijagós Archipelago of Guinea-Bissau: the MATAMAL trial
Source: BMJ Open. 2023 Jul 7;13(7):e072347. doi: 10.1136/bmjopen-2023-072347 (PMC10335573; doi:10.1136/bmjopen-2023-072347)
Supplement: Supplementary data [file bmjopen-2023-072347supp004.pdf]

MATAMAL v1.0 22/01/2020

CONSENT FORM (ADULT)

MATAMAL (CONSENT TO MASS DRUG ADMINISTRATION)

Ivermectin Mass Drug Administration for Malaria Control on the Bijagós Archipelago of Guinea Bissau: A cluster randomised placebo-controlled trial

A study conducted by the London School of Hygiene and Tropical Medicine (UK), The Ministry of Public Health (Guinea Bissau), The National Institute of Public Health (INASA), The Bandim Health Project and MRC @ LSHTM The Gambia Unit (The Gambia)

(Principal Investigator/Responsible Researcher: Dr Anna Last)

Participant's Name ____

Participant's Identification Number: |__|__|__|__|__|__|__|__|__|__|__|

Tick as appropriate

☐ I have read the written information **OR**
☐ I have had the information explained to me by study personnel in a language that I understand
☐ I confirm that my choice to participate is entirely voluntary and that I can withdraw consent at any time
☐ I confirm that I have had the opportunity to ask questions about this study and I am satisfied with the answers and explanations that have been provided by the study team
☐ I understand that I am allowing access to information about/from me to authorised persons described in the information sheet
☐ I understand that information about/from me may be shared on a public data repository or with other researchers but that I will not be identifiable from this information
☐ I have received sufficient time to consider my participation in this study
☐ I agree to take part in this study

Participant's signature/thumbprint* [ ] ________________________
Date (dd/mmm/yyyy) Time (24hr)

Printed name of witness* ____

Printed Name of Person obtaining consent ____

I attest that I have explained the study information accurately in ________________________ (*insert language*) to, and was understood to the best of my knowledge by, the participant/parent/guardian. He/she has freely given consent to participate in the presence of the above named witness*.

Signature of Person obtaining consent ________________________ ________________________
Date (dd/mmm/yyyy) Time (24hr)

** Only required if the participant is unable to read or write.*

A copy of this informed consent document has been provided to the participant
