# Supplementary material for: Protocol for a cluster randomised placebo-controlled trial of adjunctive ivermectin mass drug administration for malaria control on the Bijagós Archipelago of Guinea-Bissau: the MATAMAL trial
Source: BMJ Open. 2023 Jul 7;13(7):e072347. doi: 10.1136/bmjopen-2023-072347 (PMC10335573; doi:10.1136/bmjopen-2023-072347)
Supplement: Supplementary data [file bmjopen-2023-072347supp005.pdf]

MDA Administration Record

MATAMAL

Cluster	Island	Village	Head of Family, Name	Head of Family, Nickname	Year
					2021 2022

Number	Name	Surname	Age	Sex	Consent			Month 1			Month 2			Month 3		
				M F	DP eligible			1	2	3	1	2	3	1	2	3
			Weight		IVM eligible		**DP**									
			________ Kg		Pregnant		**IVM**									

Number	Name	Surname	Age	Sex	Consent			Month 1			Month 2			Month 3		
				M F	DP eligible			1	2	3	1	2	3	1	2	3
			Weight		IVM eligible		**DP**									
			________ Kg		Pregnant		**IVM**									

Number	Name	Surname	Age	Sex	Consent			Month 1			Month 2			Month 3		
				M F	DP eligible			1	2	3	1	2	3	1	2	3
			Weight		IVM eligible		**DP**									
			________ Kg		Pregnant		**IVM**									

Number	Name	Surname	Age	Sex	Consent			Month 1			Month 2			Month 3		
				M F	DP eligible			1	2	3	1	2	3	1	2	3
			Weight		IVM eligible		**DP**									
			________ Kg		Pregnant		**IVM**									

Number	Name	Surname	Age	Sex	Consent			**Month 1**			**Month 2**			**Month 3**		
				M F	DP eligible			1	2	3	1	2	3	1	2	3
			Weight		IVM eligible		**DP**									
			________ Kg		Pregnant		**IVM**									
Number	Name	Surname	Age	Sex	Consent			**Month 1**			**Month 2**			**Month 3**		
				M F	DP eligible			1	2	3	1	2	3	1	2	3
			Weight		IVM eligible		**DP**									
			________ Kg		Pregnant		**IVM**									
Number	Name	Surname	Age	Sex	Consent			**Month 1**			**Month 2**			**Month 3**		
				M F	DP eligible			1	2	3	1	2	3	1	2	3
			Weight		IVM eligible		**DP**									
			________ Kg		Pregnant		**IVM**									
Number	Name	Surname	Age	Sex	Consent			**Month 1**			**Month 2**			**Month 3**		
				M F	DP eligible			1	2	3	1	2	3	1	2	3
			Weight		IVM eligible		**DP**									
			________ Kg		Pregnant		**IVM**									
Number	Name	Surname	Age	Sex	Consent			**Month 1**			**Month 2**			**Month 3**		
				M F	DP eligible			1	2	3	1	2	3	1	2	3
			Weight		IVM eligible		**DP**									
			________ Kg		Pregnant		**IVM**									
Number	Name	Surname	Age	Sex	Consent			**Month 1**			**Month 2**			**Month 3**		
				M F	DP eligible			1	2	3	1	2	3	1	2	3
			Weight		IVM eligible		**DP**									
			________ Kg		Pregnant		**IVM**									
