# Supplementary material for: Protocol for a cluster randomised placebo-controlled trial of adjunctive ivermectin mass drug administration for malaria control on the Bijagós Archipelago of Guinea-Bissau: the MATAMAL trial
Source: BMJ Open. 2023 Jul 7;13(7):e072347. doi: 10.1136/bmjopen-2023-072347 (PMC10335573; doi:10.1136/bmjopen-2023-072347)
Supplement: Supplementary data [file bmjopen-2023-072347supp007.pdf]

MATAMAL
Adjunctive Ivermectin Mass Drug Administration for Malaria Control: A cluster-randomised placebo-controlled trial
Standard Operating Procedure

DNA extraction of dry blood spots (DBS)	
SOP Ref:	MATAMAL/SOP/J2
Version:	1.0
Authors:	Hristina Vasileva
Effective Date:	21.01.2020
Review by:	
Approved by:	
Approval Date:	
Signed by:	

Version	Date	Reason for Change
1.0	21.01.2020	N/A

Hutchins H, *et al. BMJ Open* 2023; 13:e072347. doi: 10.1136/bmjopen-2023-072347

Date: 21.01.2020

Equipment and Consumables:

- PureLink™ Pro 96 Genomic DNA purification Kit (cat No: K182104A)
- 96 well 0.8mL polypropylene deepwell storage plate (cat No: AB0765)
- Filter Tips (200µL; 1200µL)
- Multichannel pipette (200µL; 1200µL)
- 96 well plate centrifuge
- Water bath
- 200mL measuring cylinder/ 50mL Stripettes

Reagents:

- 99.9% pure Ethanol

Preparation of Buffer Solutions (according to PureLink™ Pro 96 Genomic DNA purification kit guidelines)

1. **Wash buffer 1:**
- Add 150mL of 99.9% pure Ethanol to the Wash Buffer 1 bottle and mix well.
- Indicate that ethanol is added and note the date on the bottle.
2. **Wash buffer 2:**
- Add 175mL of 99.9% pure Ethanol to the Wash Buffer 2 bottle and mix well. #
- Indicate that ethanol is added and note the date on the bottle.
3. **Digestion buffer*:**
- Add 20µL Proteinase K (PTK) to 180µL Genomic Digestion (GD) buffer per sample and mix well.
- Make as much buffer as needed each day and allow for 10% access as follow:
  - For 1x96 well plate: 2200µL PTK to 19800µL GD buffer
  - For 2x96 well plates: 4400µL PTK to 39600µL GD buffer
  - For 3x96 well plates: 6600µL PTK to 59400µL GD buffer
  - For 4x96 well plates: 8800µL PTK to 79200µL GD buffer
4. **Lysing binding buffer:**
- Add 200µL of 99.9% pure Ethanol (EtOH) to 200µL genomic lysing-binding (L-B) buffer per sample and mix well.
- Make as much buffer as needed each day and allow for 10% access as follow:
  - For 1x96 well plate: 22000µL EtOH to 22000µL L-B buffer
  - For 2x96 well plates: 44000µL EtOH to 44000µL L-B buffer
  - For 3x96 well plates: 66000µL EtOH to 66000µL L-B buffer
  - For 4x96 well plates: 88000µL EtOH to 88000µL L-B buffer

- ***If the digestion buffer appears stringy with small crystals, put it in a warm incubator for 30 minutes before using (or until the crystals have dissolved). The stringiness indicates that the temperature in the room where the kit is stored is below the normal room temperature.***

Hutchins H, *et al. BMJ Open* 2023; 13:e072347. doi: 10.1136/bmjopen-2023-072347

Laboratory Procedure

1. Prepare the needed buffers as described above.
2. Take up to 4 96 deep well plates containing punched DBS as described in MATAMAL SOP J1.
3. Switch on the water bath and set it to 55°C.
4. Using filter tips and multichannel pipette, add 200µL of digestion buffer to each well to completely immerse the filter paper pieces (press with the tips if pieces and not immersed).
5. Change tips for each sample.
6. Seal the plate with foil tape provided in the kit and vortex the plate for 5 seconds.
7. Incubate the seal plate in the water bath (at 55°C) for 30 minutes.
8. Centrifuge the plate at 2100g (RFC) for 3 minutes at room temperature (20°C) to pellet the paper fibres.
9. In a new 96 deep well plate add 20µL RNase A per well and label it.
10. Add the 200µL of digest material without paper fibres to the 96 well plate containing the RNase A

- **Make sure you keep the order of the samples (eg: transfer A1 to A1, B1 to B1 etc).**

11. Prepare the 96 well column plate included in the kit on top of a fresh 96 deep well plate and label it.
12. Add 400µL of genomic lysis/binding buffer to each well containing the digest and the RNase A

- **Use the same tip to transfer the lysate to the assembled 96 well column plate.**
- **Make sure again that the sample order is kept while transferring.**

13. Seal the stacked plate with foil tape and centrifuge at 2100g for 10 minutes.
14. Discard the flow throw, collected in the deep well, and place the column plate back on top.
15. Add 500µL Wash buffer 1 into each well of the column plate
16. Centrifuge the stacked plate at 2100g for 5 minutes.
17. Discard the flow through and reassemble the stack.
18. Add 500µL Wash buffer 2 to each well of the column plate.
19. Centrifuge the stacked plate at 2100g for 5 minutes.
20. Discard the flow through and reassemble the stack.
21. Centrifuge the empty stack at 2100g for 5 minutes.
22. Discard the deep well pate and stack the column plate on top of a fresh 96 deep well plate.
23. Add 80µL of Elution buffer to each well and incubate on the bench for 10 minutes.
24. Centrifuge the stack at 2100g for 3 minutes.
25. Take the elution (flow through) and add it back on the column.
26. Reassemble the column on top of the same plate.

- **Very crucial step to not cross contaminate as the elution contains the DNA.**

27. Incubate at room temperature for 10 minutes.
28. Centrifuge at 2100g for 3 minutes.
29. Discard the column plate and seal the 96 deep well plate, containing the eluted DNA, with a foil tape.
30. Store the DNA at 4°C (fridge) for short term storage (up to 2 weeks) or at -20°C (freezer) for long term storage.

- **Avoid multiple freeze thawing.**

Hutchins H, *et al. BMJ Open* 2023; 13:e072347. doi: 10.1136/bmjopen-2023-072347
