# Supplementary material for: Protocol for a cluster randomised placebo-controlled trial of adjunctive ivermectin mass drug administration for malaria control on the Bijagós Archipelago of Guinea-Bissau: the MATAMAL trial
Source: BMJ Open. 2023 Jul 7;13(7):e072347. doi: 10.1136/bmjopen-2023-072347 (PMC10335573; doi:10.1136/bmjopen-2023-072347)
Supplement: Supplementary data [file bmjopen-2023-072347supp006.pdf]

MATAMAL

Adjunctive Ivermectin Mass Drug Administration for Malaria Control: A cluster-randomised placebo-controlled trial

Standard Operating Procedure<br>Finger Prick Blood Sampling for Dry Blood Spot Collection	
SOP Ref:	MATAMAL/SOP/C9
Version:	1.0
Authors:	Harry Hutchins
Effective Date:	
Review by:	
Approved by:	
Approval Date:	
Signed by:	

SOP Chronology

Version	Date	Reason for Change	Author
1.0		N/A	

Hutchins H, *et al. BMJ Open* 2023; 13:e072347. doi: 10.1136/bmjopen-2023-072347

Table of Contents	Page
1. Purpose	3
2. Policy Statement	3
3. Background	3
4. Scope	3
5. Responsibilities	3
6. Equipment	3
7. Procedure	4
8. References	5
9. Appendices	

Hutchins H, *et al. BMJ Open* 2023; 13:e072347. doi: 10.1136/bmjopen-2023-072347

Purpose

This Standard Operating Procedure (SOP) describes the technique for obtaining uniform and high-quality dry blood spot (DBS) samples to facilitate laboratory analysis and reduce harm to participants.

Policy Statement

All LSHTM SOPs will be produced and approved in accordance with the LSHTM SOP on SOPs. All studies sponsored or hosted by LSHTM, including within the LSHTM Clinical Trials Unit, should comply with these procedures and must be used in conjunction with overarching LSHTM policies and guidelines and all relevant legal and ethical requirements governing the conduct of the study.

Background

DBS have been an important tool for sample collection, especially in infectious disease and metabolic medicine, for over a century. They provide a robust medium for transport and preservation of clinical samples from even remote and hostile environments. They are low-cost, simple to produce and collect, and can be used for complex laboratory analysis.[1,2]

Development of techniques in serological and molecular analysis mean a simple DBS sample can now be used to identify and quantify a variety of infections as part of a single assay[3]. This can then be compared with other diagnostic techniques, timepoints and locations to improve understanding of disease epidemiology.

A structured SOP combined with effective training ensures that high-quality DBS are taken on properly prepared booklets, improving the likelihood of successful and accurate analysis on delivery to the laboratory.

Scope

This SOP applies to the collection of all DBS in the context of the MATAMAL trial.

Responsibilities

Clinician: informing participants and taking DBS using the correct method.

Field assistant: identifying participants and ensuring informed consent is in place. Safely storing DBS. Assisting the clinician as directed.

Equipment

- Informed consent/assent form
- Non-sterile gloves
- Hand sanitiser
- Clinical waste bag
- Sharps bin
- Alcohol wipe
- Lancet (paediatric or adult)
- Cotton wool balls
- Pre-made DBS booklet (see appropriate SOP)
- Zip-lock/sealable plastic bag or box
- Elastic band

- Silica gel (1g sachets)

Procedure[1,4]

1. Ensure informed consent is in place and explain procedure to participant.
2. Ensure sample ID is affixed to the DBS booklet and documented alongside participant ID.
3. Sanitise hands and don gloves.
4. It is important that no-one touches the filter paper without gloves.
5. Collect the sample:
    a. Clean the pulp of the 4th finger of the participants' non-dominant hand with an alcohol wipe. Allow to air dry.
    b. Use a lancet to prick the finger. Dispose of the lancet into a sharps bin. (Figure 1)
    c. Wipe the first bead of blood away with cotton wool.
    d. With the hand palm-down, exert gentle pressure to encourage bleeding and press the blood against the filter paper in three distinct circles. (Figure 2)
    e. Ensure enough blood in each circle to soak through to the other side. (Figure 3A, unlike Figure 3B)
    f. Allow samples to air dry to brown before folding securely.
6. Collect 10-25 DBS booklets together with an elastic band and place in a zip-lock bag or sealed box containing silica gel sachets. (Figure 4)
7. These DBS should ultimately be stored in -20°C freezers. They may be kept at -4°C or even ambient temperature for up to 14 days.
8. Discard all waste: sharps into sharps bin, other waste into clinical waste bag.

<table>
<tr><td>Figure 1</td><td>Figure 2</td></tr>
<tr><td>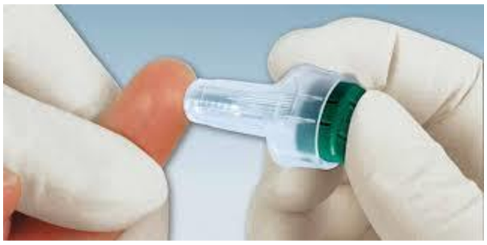</td><td>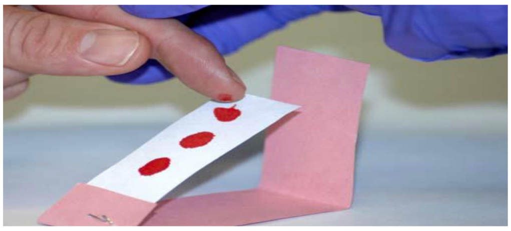</td></tr>
<tr><td>Figure 3</td><td>Figure 4</td></tr>
<tr><td>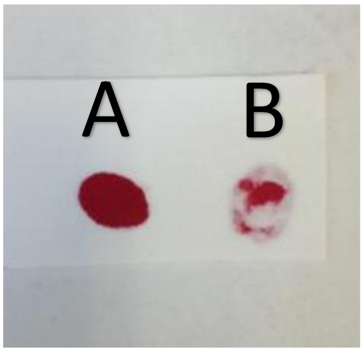
</td><td>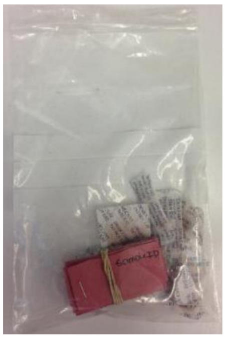</td></tr>
</table>

References

1. Grüner N, Stambouli O, Ross RS. Dried blood spots - Preparing and processing for use in immunoassays and in molecular techniques. *J Vis Exp*. 2015;2015(97). doi:10.3791/52619

2. Zakaria R, Allen KJ, Koplin JJ, Roche P, Greaves RF. Advantages and challenges of dried blood spot analysis by mass spectrometry across the total testing process. *JIFCC*. 2016;27(4):288-317.

3. Sanprasert V, Kerdkaew R, Srirungruang S, Charuchaibovorn S, Phadungsaksawasdi K, Nuchprayoon S. Development of Conventional Multiplex PCR: A Rapid Technique for Simultaneous Detection of Soil-Transmitted Helminths. *Pathogens*. 2019;8(3):152. doi:10.3390/pathogens8030152

4. Mei J V, Richard Alexander J, Adam BW, Harry Hannon W, Finley A. Use of Filter Paper for the Collection and Analysis of Human Whole Blood Specimens. *J Nutr*. 2001;131:1631S-1636S. https://academic.oup.com/jn/article-abstract/131/5/1631S/4686823. Accessed April 19, 2020.
