# Supplementary material for: Protocol for a cluster randomised placebo-controlled trial of adjunctive ivermectin mass drug administration for malaria control on the Bijagós Archipelago of Guinea-Bissau: the MATAMAL trial
Source: BMJ Open. 2023 Jul 7;13(7):e072347. doi: 10.1136/bmjopen-2023-072347 (PMC10335573; doi:10.1136/bmjopen-2023-072347)

# MATAMAL
# Adjunctive Ivermectin Mass Drug Administration for Malaria Control: A cluster-randomised placebo-controlled trial
# Standard Operating Procedure

| *Plasmodium falciparum VarATS* Quantifying Real-Time Polymerase Chain Reaction (qPCR). | |
|---|---|
| SOP Ref: | MATAMAL/SOP/J3 |
| Version: | 1.0 |
| Authors: | Hristina Vasileva |
| Effective Date: | 21.04.2020 |
| Review by: | |
| Approved by: | |
| Approval Date: | |
| Signed by: | |

| Version | Date | Reason for Change |
|---|---|---|
| 1.0 | 21.04.2020 | N/A |
| | | |
| | | |
| | | |



Hutchins H, *et al. BMJ Open* 2023; 13:e072347. doi: 10.1136/bmjopen-2023-072347

Date: 22.01.2020

## Equipment and Consumables:

- Bench top tube centrifuge
- Vortex (SLS, Serial: 12030824)
- 1.5mL DNA LowBind Eppendorf tubes (cat No: 10031282)
- Filter tips (10µL, 20µL, 200µL, 1000µL)
- Multichannel micropipettes (P10; P50)
- Singlechannel micropipettes (P10; P20; P200; P1000)
- 96 well PCR plate (Life Sciences; cat No: 04729692001)
- MicroAmp optical adhesive film (Thermofisher Scientific; cat No: 4311971)
- 1.5mL DNA LoBind Eppendorf tubes (Eppendorf; cat No: 0030108051)
- Plate centrifuge (Benchmark, Serial: E081501133)
- Bio Rad CFX996 real-time PCR machine (Serial: 785BR13221)
- Mustang Purple Dye Spectral Calibration Plate, 96-weels (Life Technologies; cat No:4461599)

## Reagents:

- TaqMan™ Universal Master Mix (Applied Biosystems; cat No: 4304437) stored at 4°C
- *P. falciparum varATS* forward primer (5'- CCCATACACAACCAAYTGGA -3') stored at -20°C
- *P. falciparum varATS* reverse primer (5'- TTCGCACATATCTCTATGTCTATCT -3') stored at -20°C
- *P. falciparum varATS* probe (6FAM- TRTTCCATAAATGGT-NFQ-MGB) stored at -20°C
- Nuclase-free water (Qiagen; cat No: 129114) *stored at 4°C
- Bleach (Madar)
- Ethanol (VWR; cat No: 20821.321)

## Laboratory Procedure:

1. Generate a plate plan with corresponding sample IDs which should include the following and shown in Table 1:
- Serial dilution standards with known parasitaemia (for trend-line and PCR positive control), refer to MATAMAL SOP J…
- 
2. Make 500µL of 10µM forward and reverse primers.
- Original concentration of primers: 100µM
- Dilution factor to make 10µM stock: 100/10=10
- For 500µL primer mix use 500/10=50µL of each primer
- Add 400µL of Nuclease-free water to make up the volume to 500µL
3. Make 500µL of 4µM FAM probe.
- Original concentration of probe: 100µM
- Dilution factor to make 4µM stoke: 100/4=25
- For 500µL probe mix use 500/25=20µL of FAM probe
- Add 480µL of Nuclease-free water to make the volume up to 500µL3.
4. Calculate volume of 10µM primers and 4µM probe needed for 1 qPCR reaction of 20µL.
- TaqMan Multiplex Master mix: 10µL
- 100nM/20µL primers: 10/0.2=50 (dilution factor); 20/50=0.4µL of 10µM stoke
- 80nM/20µL probe: 4/0.2=50 (dilution factor); 20/50=0.4µL of 4µM stoke
- Sample/Standards/Control DNA: 5µL
- Nuclease-free water: 4.2µL
5. Working in a DNA hood, make master mix (excluding the sample volume) enough for 110 samples (to allow for excess) in a 2mL DNA LowBind Eppendorf.
- TAqMan Multiplex Master Mix: 110*10=1100µL
- Primers: 110*0.4=44µL of 10µM stock
- Probe: 110*0.4=44µL of 4µM stock
- Nuclease-free water: 110*4.2=462µL
6. Vortex the master mix for 10 seconds.
7. Using a repetitive pipette, pipette 15µL in each well of a 96 well optical plate.
8. Take everything out of the DNA hood and UV blast it.
9. On the working bench, pipette 5µL of Samples, Standards and Controls as shown in Figure1 and mix by aspirating up and down multiple times.

**Figure1**



Hutchins H, *et al. BMJ Open* 2023; 13:e072347. doi: 10.1136/bmjopen-2023-072347

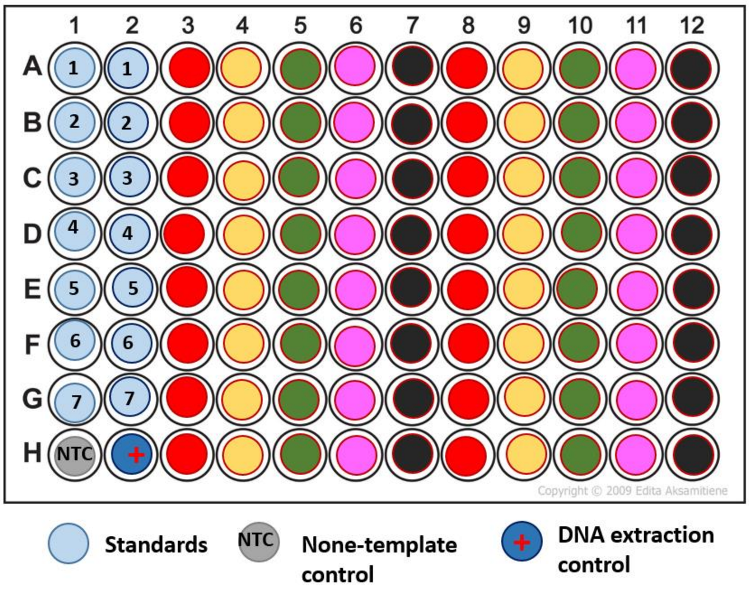


10. The grid of Figure1 shows the organization of samples and standards, where same colours indicate the same samples in duplicates
    - Eg: Columns 3 and 8 contain the same samples in the exact same order.
    - None-template control is 5µL of Nuclease-free water.
    - DNA extraction control information is found in MATAMAL SOP J2.
11. Seal the plate with optical adhesive film and press around the edges.
12. Centrifuge the plate in the plate centrifuge for 5 seconds and make sure that there are no bubbles on the bottom of the wells.
13. Run the plate an Applied Biosystems 7500 Real-Time PCR machine at the absolute quantification mode.
14. Set the parameters of the run as shown below*.

- ***Ensure that MUSTANG PURPLE is selected as a reference dye. If MUSTANG PURPLE is not present as option, a calibration using the Mustang Purple Dye Spectral Calibration Plate must be performed.***
- ***Ensure that the amplification cycle number is set as 45, rather than the default set up of 40 cycles.***



Hutchins H, *et al. BMJ Open* 2023; 13:e072347. doi: 10.1136/bmjopen-2023-072347

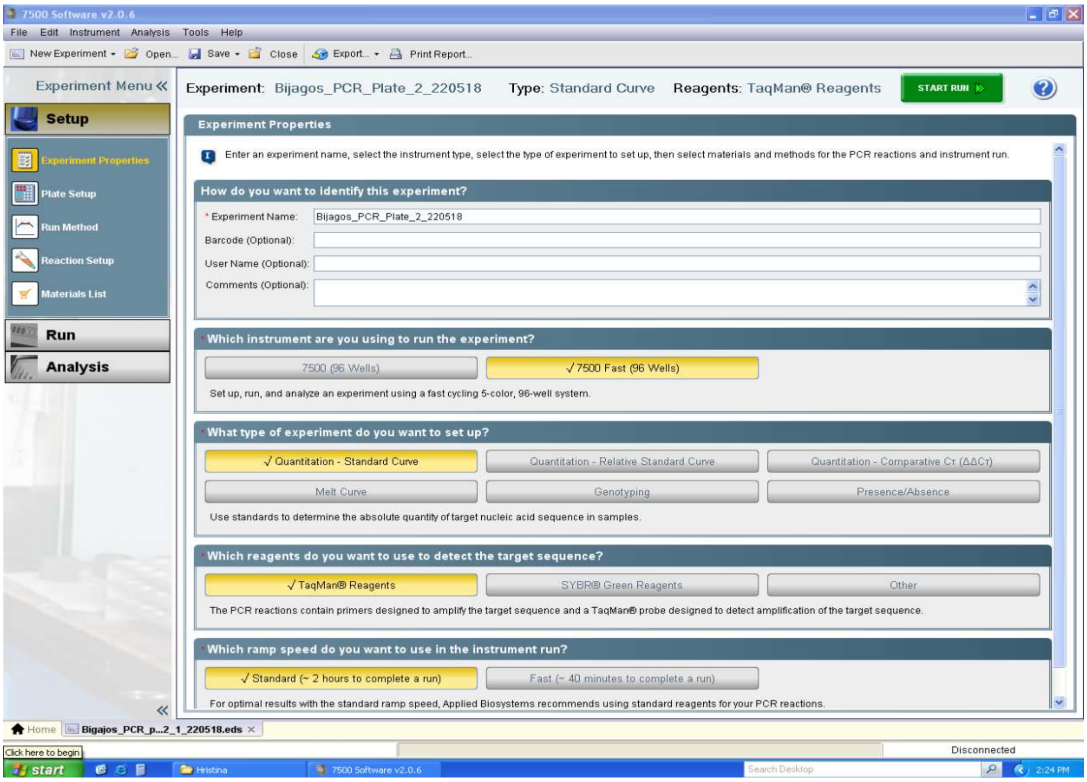
7500 Software v2.0.6
File Edit Instrument Analysis Tools Help
New Experiment Open... Save Close Export... Print Report...
Experiment Menu
Setup
Experiment Properties
Plate Setup
Run Method
Reaction Setup
Materials List
Run
Analysis
Experiment: Bijagos_PCR_Plate_2_220518
Type: Standard Curve
Reagents: TaqMan® Reagents
START RUN
Experiment Properties
Enter an experiment name, select the instrument type, select the type of experiment to set up, then select materials and methods for the PCR reactions and instrument run.
How do you want to identify this experiment?
* Experiment Name: Bijagos_PCR_Plate_2_220518
Barcode (Optional):
User Name (Optional):
Comments (Optional):
Which instrument are you using to run the experiment?
7500 (96 Wells)
√ 7500 Fast (96 Wells)
Set up, run, and analyze an experiment using a fast cycling 5-color, 96-well system.
What type of experiment do you want to set up?
√ Quantitation - Standard Curve
Quantitation - Relative Standard Curve
Quantitation - Comparative CT (ΔΔCT)
Melt Curve
Genotyping
Presence/Absence
Use standards to determine the absolute quantity of target nucleic acid sequence in samples.
Which reagents do you want to use to detect the target sequence?
√ TaqMan® Reagents
SYBR® Green Reagents
Other
The PCR reactions contain primers designed to amplify the target sequence and a TaqMan® probe designed to detect amplification of the target sequence.
Which ramp speed do you want to use in the instrument run?
√ Standard (~ 2 hours to complete a run)
Fast (~ 40 minutes to complete a run)
For optimal results with the standard ramp speed, Applied Biosystems recommends using standard reagents for your PCR reactions.
Home
Bigajos_PCR_p...2_1_220518.eds
Click here to begin
Disconnected
start
Hristina
7500 Software v2.0.6
Search Desktop
2:24 PM

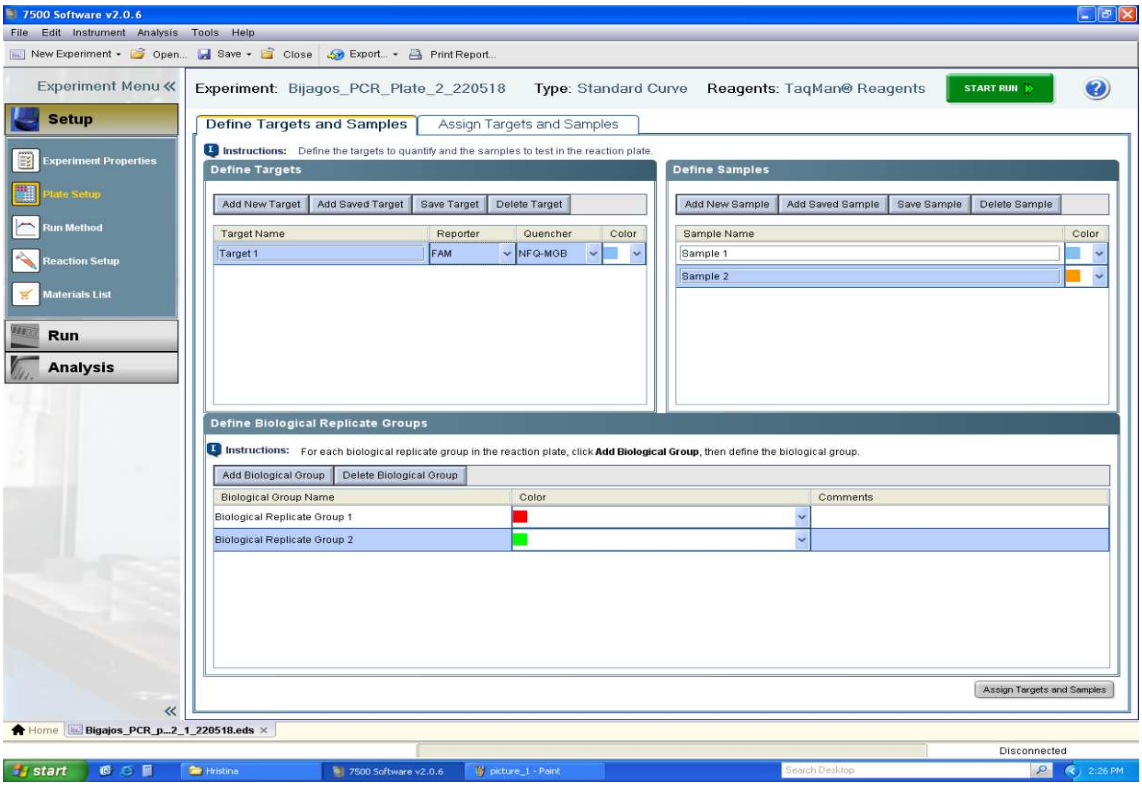

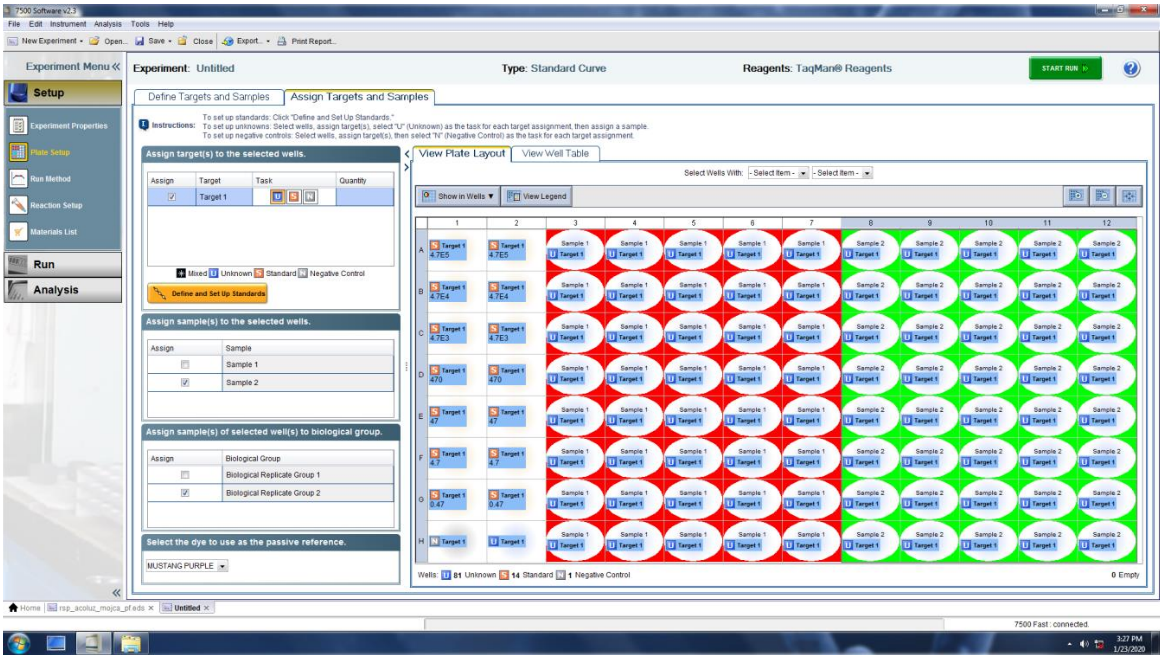



Hutchins H, *et al. BMJ Open* 2023; 13:e072347. doi: 10.1136/bmjopen-2023-072347

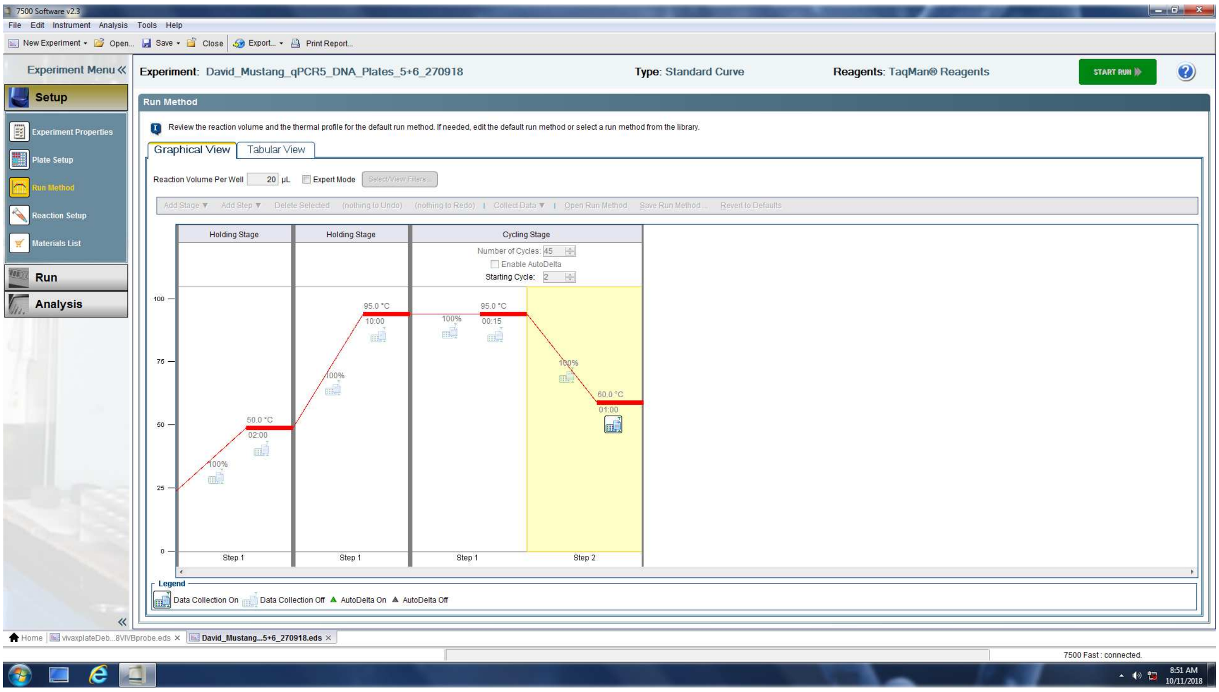

15. Select threshold and baseline value based on the Standards output and investigate the results quality using output plots show below.

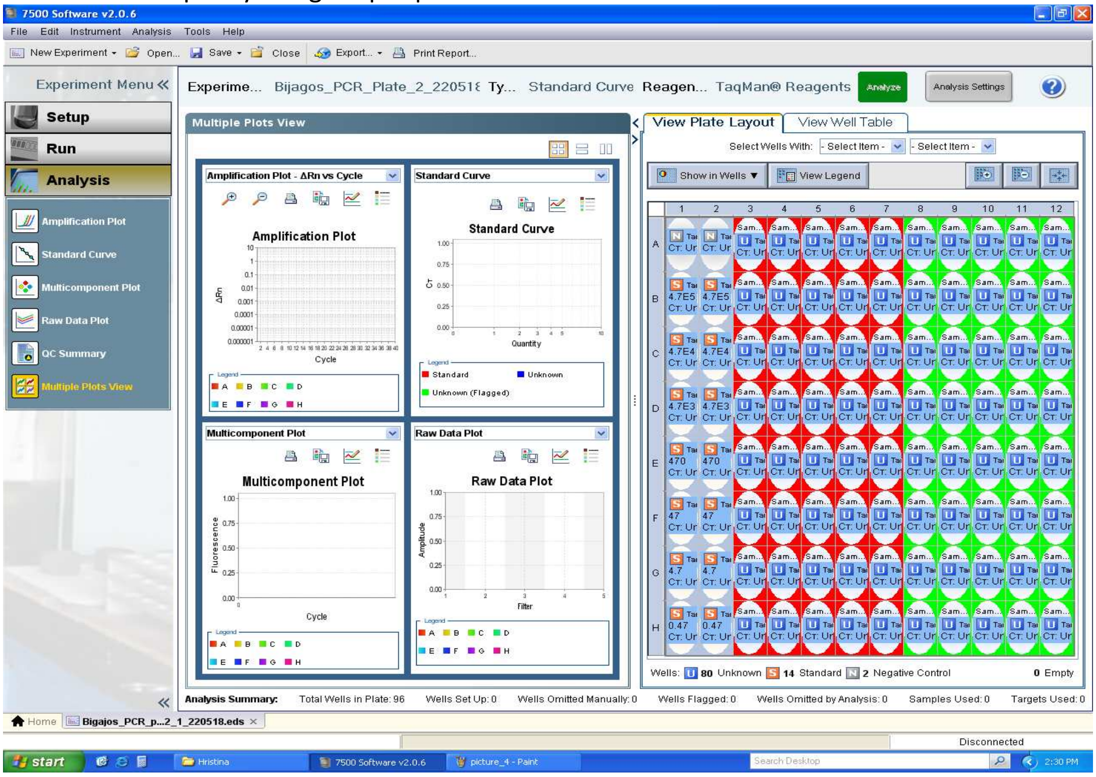

Supplement: Supplementary data [file bmjopen-2023-072347supp008.pdf]
