# Supplementary material for: Protocol for a cluster randomised placebo-controlled trial of adjunctive ivermectin mass drug administration for malaria control on the Bijagós Archipelago of Guinea-Bissau: the MATAMAL trial
Source: BMJ Open. 2023 Jul 7;13(7):e072347. doi: 10.1136/bmjopen-2023-072347 (PMC10335573; doi:10.1136/bmjopen-2023-072347)
Supplement: Supplementary data [file bmjopen-2023-072347supp009.pdf]

Membership of Trial Oversight Bodies

MATAMAL Clinical Trial

All appointments have been approved, and made in line with the guidelines issued by, the trial funders and independent ethics review boards. All bodies are independent of the sponsor. All members declare that they have no competing interests.

Trial Steering Committee

- Steve Lindsay (***Independent Chair***)
  Professor of Public Health Entomology
  Durham University, Durham, UK
- Menno Smit
  Honorary Associate Professor
  LSTM, Liverpool, UK
- Thom Eisele
  Director, Applied Malaria Research and Evaluation
  Tulane University School of Public Health and Tropical Medicine, USA
- Oumar Gaye
  Professor of Medical Parasitology
  University Cheikh Anta Diop, Dakar, Senegal
- Anna Last (***Principal Investigator***)
  Associate Professor in Infectious Diseases
  LSHTM, London, UK
- David Mabey
  Professor of Communicable Diseases
  LSHTM, London, UK
- Umberto D'Alessandro
  Director
  MRC The Gambia @ LSHTM, Fajara, The Gambia
- Amabelia Rodrigues (***Local Principal Investigator***)
  Director of Research
  Bandim Health Project, Bissau, Guinea-Bissau
- John Bradley (***Trial Statistician***)
  Assistant Professor of Medical Statistics and Epidemiology
  LSHTM, London, UK
- Harry Hutchins (***Clinical Trial Manager***)
  Clinical Research Fellow
  LSHTM, London, UK

Trial Steering Committee Observers

- Immo Kleinschmidt

Professor of Epidemiology
LSHTM, London, UK

- Brian Greenwood
  Professor of Clinical Tropical Medicine
  LSHTM, London, UK
- MRC appointed observer (Jo Mulligan, FCDO)
- LSHTM appointed observer (Sponsor/Host)

Data Safety Monitoring Board

- Chi Eziefula
  Clinical Epidemiologist
  Brighton and Sussex Medical School, Brighton, UK
- Johan Ursing
  Clinical Epidemiologist
  Karolinska Institute, Sweden
- Mahamadou Ali Thera (***Chair***)
  Malariologist
  University of Sciences Techniques and Technology, Bamako, Mali
- Jennifer Smith
  Epidemiologist and Biostatistician
  University of California, San Francisco, USA
