# Supplementary material for: Protocol for a cluster randomised placebo-controlled trial of adjunctive ivermectin mass drug administration for malaria control on the Bijagós Archipelago of Guinea-Bissau: the MATAMAL trial
Source: BMJ Open. 2023 Jul 7;13(7):e072347. doi: 10.1136/bmjopen-2023-072347 (PMC10335573; doi:10.1136/bmjopen-2023-072347)
Supplement: Author's manuscript [file bmjopen-2023-072347.draft_revisions.pdf]

BMJ Open is committed to open peer review. As part of this commitment we make the peer review history of every article we publish publicly available.

When an article is published we post the peer reviewers' comments and the authors' responses online. We also post the versions of the paper that were used during peer review. These are the versions that the peer review comments apply to.

The versions of the paper that follow are the versions that were submitted during the peer review process. They are not the versions of record or the final published versions. They should not be cited or distributed as the published version of this manuscript.

BMJ Open is an open access journal and the full, final, typeset and author-corrected version of record of the manuscript is available on our site with no access controls, subscription charges or pay-per-view fees (http://bmjopen.bmj.com).

If you have any questions on BMJ Open's open peer review process please email info.bmjopen@bmj.com

Protocol for a cluster randomised placebo-controlled trial of adjunctive ivermectin mass drug administration for malaria control on the Bijagós Archipelago of Guinea-Bissau: The MATAMAL trial

Journal:	*BMJ Open*
Manuscript ID	bmjopen-2023-072347
Article Type:	Protocol
Date Submitted by the Author:	30-Jan-2023
Complete List of Authors:	Hutchins, Harry; London School of Hygiene & Tropical Medicine, Clinical Research Department<br>Bradley, John; London School of Hygiene and Tropical Medicine<br>Pretorius, Elizabeth; London School of Hygiene & Tropical Medicine, Department of Disease Control<br>Teixeira da Silva, Eunice; Projecto de Saúde Bandim; Ministério de Saúde Pública<br>Vasileva, Hristina; London School of Hygiene & Tropical Medicine, Department of Infection Biology<br>Jones, Robert; London School of Hygiene & Tropical Medicine, Department of Disease Control<br>Ndiath, Mamadou; Medical Research Council The Gambia<br>dit Massire Soumare, Harouna; Medical Research Council The Gambia<br>Mabey, David; London School of Hygiene & Tropical Medicine, Clinical Research Department<br>Nante, Ernesto; Ministério de Saúde, Programa Nacional de Luta Contra o Paludismo<br>Martins, Cesario; Projecto de Saúde Bandim<br>Logan, James; London School of Hygiene & Tropical Medicine, Department of Disease Control<br>Slater, Hannah; PATH<br>Drakeley, Chris; London School of Hygiene & Tropical Medicine, Department of Infection Biology<br>D'Alessandro, Umberto; Medical Research Council The Gambia<br>Rodrigues, Amabelia; Projecto de Saúde Bandim; Ministério de Saúde Pública<br>Last, Anna R.; London School of Hygiene & Tropical Medicine, Clinical Research Department
Keywords:	Clinical trials < THERAPEUTICS, EPIDEMIOLOGIC STUDIES, Public health < INFECTIOUS DISEASES

Protocol for a cluster randomised placebo-controlled trial of adjunctive ivermectin mass drug administration for malaria control on the Bijagós Archipelago of Guinea-Bissau: The MATAMAL trial

Harry Hutchins*[1], John Bradley[1], Elizabeth Pretorius[2], Eunice Teixeira da Silva[3,4], Hristina Vasileva[1,5], Robert T Jones[2], Mamadou Ousmane Ndiath[6], Harouna dit Massire Soumare[6], David Mabey[1], Ernesto Jose Nante[7], Cesario Martins[3], James G Logan[2,8], Hannah Slater[9], Chris Drakeley[5], Umberto D'Alessandro[6], Amabelia Rodrigues[3,4], Anna Last[1].

1. Clinical Research Department, London School of Hygiene & Tropical Medicine (LSHTM), London, UK
2. Department of Disease Control, LSHTM, London, UK
3. Projecto de Saúde Bandim, Bissau, Guinea-Bissau
4. Ministério de Saúde Pública, Bissau, Guinea-Bissau
5. Department of Infection Biology, LSHTM, London, UK
6. Medical Research Council Unit The Gambia, Fajara, The Gambia
7. Programa Nacional de Luta Contra o Paludismo, Ministério de Saúde, Bissau, Guinea-Bissau
8. Arctech Innovation, London, UK
9. PATH, Seattle, USA

*Corresponding author
Email: harry.hutchins@lshtm.ac.uk
Address: Office 359, LSHTM, London, WC1E7HT, UK

Word count: 3932

For peer review only - http://bmjopen.bmj.com/site/about/guidelines.xhtml

KEY WORDS

- Clinical Trial
- Mass Drug Administration
- Guinea-Bissau
- Malaria
- Neglected Tropical Diseases
- Ivermectin
- Mosquito Vectors

ABSTRACT

Introduction

As malaria declines, innovative tools are required to further reduce transmission and achieve elimination. Mass drug administration (MDA) of artemisinin-based combination therapy (ACT) is capable of reducing malaria transmission where coverage of control interventions is already high, though the impact is short-lived. Combining ACT with ivermectin, an oral endectocide shown to reduce vector survival, may increase its impact whilst also treating ivermectin-sensitive co-endemic diseases and minimizing the potential impact of ACT resistance in this context.

Methods and Analysis

MATAMAL is a cluster-randomised placebo-controlled trial. The trial is being conducted in 24 clusters on the Bijagos Archipelago, Guinea-Bissau where the peak prevalence of *Plasmodium falciparum* (*Pf*) parasitaemia is approximately 15%. Clusters have been randomly allocated to receive MDA with dihydroartemisinin-piperaquine and either ivermectin or placebo. The primary objective is to determine whether the addition of ivermectin MDA is more effective than dihydroartemisinin-piperaquine MDA alone in reducing the prevalence of *Pf* parasitaemia, measured during peak transmission season after two years of seasonal MDA. Secondary objectives include assessing prevalence after one year of MDA; malaria incidence monitored through active and passive case surveillance; age-adjusted prevalence of serological markers indicating exposure to *Pf* and anopheline mosquitoes; vector parous rates, species composition, population density and sporozoite rates; prevalence of vector pyrethroid resistance; prevalence of artemisinin resistance in *Pf* using genomic markers; the impact of ivermectin on co-endemic diseases; coverage estimates, and the safety of combined MDA through monitoring of adverse events.

Ethics and Dissemination

The trial has been approved by the London School of Hygiene & Tropical Medicine Ethics Committee (UK) (19156) and the Comite Nacional de Eticas de Saude (Guinea-Bissau) (084/CNES/INASA/2020). Results will be disseminated in peer-reviewed publications and in discussion with the Bissau-Guinean Ministry of Public Health, the regional health directorate and participating communities.

Trial Registration

The trial is registered with ClinicalTrials.gov (NCT04844905). Last updated 24/02/22.

STRENGTH AND LIMITATIONS OF THE STUDY

- This is the only clinical trial to evaluate the additive impact of mass administered

For peer review only - http://bmjopen.bmj.com/site/about/guidelines.xhtml

ivermectin with dihydroartemisinin-piperaquine for malaria control and control of co-endemic neglected tropical diseases.

- The geographical separation of island clusters minimizes effects of contamination and spillover between clusters.
- Sample size of 200 over 24 clusters achieves power of 80% for the primary outcome measure.
- The study has been developed and is being conducted in partnership with the Bissau-Guinean National Malaria Control Programme and Ministry of Public Health.
- This is a unique study setting such that results may not be directly generalisable elsewhere, although malaria endemicity and transmission dynamics appear similar to those elsewhere in the region.

INTRODUCTION

Malaria in sub-Saharan Africa has declined dramatically since 2000, with much of the decrease due to vector control methods such as insecticide-treated nets (ITN) and Indoor Residual Spraying (IRS).[1] However, these methods are threatened by increasing insecticide resistance in vectors[2] and their limited efficacy against outdoor-biting or outdoor-resting mosquitoes.[3] There is a clear need for additional vector-control methodologies, including novel tools or novel uses of existing tools.

Mass Drug Administration (MDA) with Artemisinin-based Combination Therapy (ACT) could reduce transmission where coverage of vector-control interventions is high by impacting the human parasite reservoir,[4] reaching even asymptomatic cases which help perpetuate transmission in such settings.[5] Although MDA temporarily reduces malaria prevalence there is little evidence of prolonged effect[6] and modelling predicts that without additional vector-control measures, efficacy is short-lived.[7]

Dihydroartemisinin-piperaquine (DP) is an efficacious and safe antimalarial.[8–10] Piperaquine's long half-life makes it attractive for ACT MDA.[11–14] A cluster-randomised controlled trial in Zambia showed reduced community-level parasite prevalence after two rounds of DP MDA to households with at least one case, particularly in low-transmission areas.[15] DP MDA in The Gambia also resulted in a significant reduction in infection incidence, however these gains were short-lived in higher transmission areas.[16]

Ivermectin (IVM) has been widely used in MDA campaigns against onchocerciasis and lymphatic filariasis (LF) in Africa for decades.[17–21] It is an effective endectocide and could be used in MDA to complement vector control strategies, particularly where existing measures have been maximized.[17,22] In West Africa, IVM MDA using 150µg/Kg decreased *An. gambiae* survival and sporozoite rates (SR).[23] Modelling predicts that three consecutive days at this dosage would reduce infectious vector populations by 68% for 60 days.[24] Three daily doses of 300 or 600µg/Kg/day were mosquitocidal at 28 days post-treatment[25,26] and whilst there was a slight increase in minor adverse events (AE) at the higher dose, significantly higher doses have been safely used to treat headlice and onchocerciasis.[18–21,27]

Several clinical trials have demonstrated that combined IVM/ACT MDA is safe, and that it remains an effective anti-malarial treatment and endectocide.[25,26,28–30] IVM/ACT MDA has been shown to be more lethal to vectors than IVM MDA alone[31] and population-level transmission modelling predicts adjunctive IVM would boost the efficacy of DP MDA in reducing malaria prevalence in both high and low prevalence settings.[24] Clinical trial data are needed to confirm these findings.

For peer review only - http://bmjopen.bmj.com/site/about/guidelines.xhtml

The RIMDAMAL trial in Burkina Faso reported that communities receiving IVM-only MDA (150-200µg/Kg), given five times at three-weekly intervals after a single dose of IVM and albendazole, saw reduced incidence of clinical malaria in young children compared to communities receiving the single dose alone.[29] However, independent statistical analysis has questioned these findings.[32] The MASSIV trial in The Gambia compared two years of IVM (300-400µg/Kg) and DP MDA on three consecutive days in three consecutive months, against no intervention.[30] It found significantly lower malaria prevalence in the intervention arm, however, it is impossible to separate the effect of IVM from DP due to the absence of MDA in the control arm. Further trials are therefore required not only to confirm optimal dosage and regimen for IVM/ACT MDA, but also its impact on malaria transmission.

The Bijagos Archipelago lies 50km off the Atlantic coast of Guinea-Bissau. Eighteen of the 88 tropical islands are inhabited, supporting a population of approximately 25,000 fishermen, hunter-gatherers and subsistence farmers. There is a long dry season alternating with a short rainy season (June – October). The mean temperature is 27.3°C with little monthly variation.[33] Qualitative surveys and daily activity mapping have shown that population movement, including for farming or ceremonies, is more limited than in continental Guinea-Bissau, likely due to the islands' remoteness and lack of transport.[34]

Malaria transmission is highly seasonal, peaking at the end of the rains. In 2018, qPCR prevalence of *Pf* infection was 8.5% in January (95% CI 6.2-10.8), 12.3% in July (95% CI 9.4-15.3) and 17.5% in November (95% CI 16.0-19.1) (manuscript in preparation). Infection is highest in the 6-15 years age-group. Bed net coverage is estimated at 92% (95% CI 86%-96%) with reportedly high usage (86%), however, intermittent preventative therapy in pregnancy (IPTp) coverage is lower and, in some areas, non-existent.[35] Indoor residual spraying is not used. One pilot round of seasonal malaria chemoprevention (SMC) was conducted on the islands during the 2020 rainy season. Indoor CDC light trapping indicates *Anopheles gambiae sensu stricto* is the predominant mosquito species during peak malaria transmission, and SR, determined by Circumsporozoite Enzyme-linked Immunosorbent Assays (CSP ELISA) suggest it is the primary malaria vector.[36]

IVM-sensitive Neglected Tropical Diseases (NTDs) such as LF, Soil-Transmitted Helminths (STH) and scabies are co-endemic, the qPCR prevalence of any STH infection being 47.3%, for instance.[37] NTD studies and control measures have demonstrated that MDA is feasible, acceptable and effective in this setting.[38]

Malaria remains a significant public health problem on the Bijagos Archipelago despite high coverage with control measures. The discrete geographical nature of the islands and the associated limitation of population and vector movement further serve to highlight the Bijagos as an ideal location for this trial.[39]

MATAMAL will provide fill an important knowledge gap as no trial has successfully assessed the effect on malaria of IVM in addition to DP MDA.[40] It will provide valuable data on the effects on co-endemic NTDs, with a view to future integrated strategies. It will complement previous studies in evaluating acceptability, feasibility and cost-effectiveness, adding to the evidence-base examining ivermectin's role in malaria control.

For peer review only - http://bmjopen.bmj.com/site/about/guidelines.xhtml

METHODS AND ANALYSIS

Study Aim

To determine whether adjunctive IVM MDA co-administered with DP MDA significantly reduces the population-based prevalence of *Pf* parasitaemia during peak malaria transmission season compared to DP MDA alone.

Study Design

Quadruple-blind (participant, intervention provider, investigator, analyst) cluster-randomised placebo-controlled trial.

Twenty-four clusters have been assigned in a 1:1 ratio to one of two trial arms using restricted randomisation[41]:

1. Intervention (DP and IVM MDA)
2. Control (DP and IVM-placebo MDA)

Restriction variables included population, baseline *Pf* prevalence (qPCR and RDT), vector density, SMC coverage and presence of a health centre. 100,000 randomisations were performed and the final randomisation selected from those satisfying the criteria.

Population and Setting

The trial is being conducted on the Bijagos Archipelago, Guinea-Bissau (Figure 1). Government projections using the last formal census (2009) estimate a population of 25589.[42] Individual islands will constitute 15 clusters. Three larger islands will each be subdivided into 3 clusters providing 24 clusters in total (Figure 2). There will be buffer zones of at least 2.2km between settlements in different clusters using a modified fried-egg principle.[41] Only one populated island, Soga, will be excluded as its very high baseline malaria prevalence is an outlier.

All residents of the islands, defined as anyone sleeping on the island for the majority of a given month, will be invited to participate unless they meet any of the following exclusion criteria:

1. Severe illness
2. Age under 6 months (DP)
3. Height under 90cm or weight under 15Kg (IVM/placebo)
4. Pregnancy (any trimester) or breastfeeding (IVM/placebo). Pregnancy (first trimester) (DP)
5. Known hypersensitivity to either medication
6. Concomitant use of drugs affecting cardiac function or the QTc interval (DP)
7. Travel to a country endemic for *Loa loa* (IVM/placebo)

Residents excluded from MDA will still be eligible for participation in surveys.

Informed written consent will be obtained after providing written/spoken information (according to literacy) in participants' own language. An independent witness will sign all consent forms of illiterate participants. Parents/guardians will be asked to consent on behalf of all children under 18 years. Children aged 12-17 will be invited to sign informed assent forms.

Intervention and Control

The intervention will be entire community MDA using:

1. Dihydroartemisinin-piperaquine, Alfasigma, Italy
2. Ivermectin, Laboratorio Elea Phoenix, Argentina

For peer review only - http://bmjopen.bmj.com/site/about/guidelines.xhtml

3. IVM placebo, Laboratorio Elea Phoenix, Argentina

Standard National Malaria Control Programme interventions (triennial distribution of bed nets, IPTp, case detection and treatment with artemether-lumefantrine) will continue in both arms. The intervention arm will receive DP and IVM, the control arm will receive DP and placebo (Figure 3). A full course of MDA involves three sequential daily doses of both medications, given monthly in July, August and September of 2021 and 2022. Monthly MDA will begin in all clusters simultaneously, with at least 28 days between each round. Distribution will be conducted by community health workers (Agentes de Saude Communitaria: ASCs) in their own villages, supervised by cluster-level field assistants. Participant age, sex, weight, pregnancy and eligibility will be recorded by household.

DP is a rapidly-acting artemisinin-based schizontocidal drug used to treat uncomplicated malaria. It is safe, well tolerated, efficacious in clearing *Pf* infection and exhibits prophylactic activity for approximately four weeks.[43]

IVM is an avermectin, active against a range of human parasitic infections and infestations. It is active against *Anopheles spp*. at concentrations present in human blood post-ingestion.[44]

All treatment will be directly observed. Doses will be tablet formulations taken with water, with no food for three hours before or after. IVM and IVM-placebo will be visually-identical 6mg tablets dosed at 300µg/Kg to the nearest tablet. DP will be available at doses of 20/160mg and 40/320mg per tablet, administered according to bodyweight. DP can be crushed and mixed with water, and will be readministered in case of vomiting within 30 minutes (half-dose if between 30-60 minutes).

Before delivery to the field, an independent pharmacy team will relabel all ivermectin and placebo bottles with identical "ivermectin or placebo" labels bearing pre-printed cluster codes and recoded lot numbers to prevent unblinding. Only the pharmacist and the independent statistician who conducted the randomisation and anonymised the labels will be unblinded. All laboratory samples will bear only alphanumeric codes. Statistical analysis will be conducted by a blinded statistician. Unblinding will only occur after completion of the primary analysis.

Outcome Measures

Primary Outcome

The population-based qPCR prevalence of *Pf* parasitaemia in all age groups during the peak malaria transmission season after two years of intervention.

Secondary Outcomes

1. Population-based prevalence of *Pf* parasitaemia in all ages, detected by qPCR, during the peak transmission season after the first year of MDA.
2. Incidence of clinical malaria confirmed by pLDH/HRP2 Rapid Diagnostic Test (RDT), determined through passive surveillance of all malaria cases presenting to health facilities throughout the trial.
3. Incidence of clinical malaria identified by RDT (CareStart Malaria PAN pLDH) during active surveillance of a cohort of children aged 5-14 years, during the intervention and peak transmission season.
4. Incidence of malaria infection identified by qPCR and serological analysis during the same period in this cohort of children.
5. Age-adjusted prevalence of serological markers indicating recent exposure to *Pf*.
6. Prevalence of serological markers of recent *Anopheles* exposure.

For peer review only - http://bmjopen.bmj.com/site/about/guidelines.xhtml

7. Parity, as a measure of *An. gambiae sensu latu* survival, measured in mosquitoes caught using indoor CDC light traps 7-14 days after the final MDA round in years 1 and 2.
8. Mosquito species composition, population density and SR in mosquitoes caught using indoor CDC light traps.
9. Prevalence of resistance to pyrethroids in anopheline mosquitoes using bioassay methodologies.
10. Prevalence of resistance to artemisinin and partner drugs in humans using molecular markers of resistance.
11. Safety of intervention through monitoring of AE.
12. Impact on IVM-susceptible NTDs (scabies, strongyloidiasis, other STHs and LF), headlice and bedbug infestation using clinical and serological parameters.
13. Cluster-level intervention coverage estimates.
14. MDA acceptability, feasibility and access.
15. Cost-effectiveness of adjunctive IVM in this setting.

Assessment of Outcomes

Primary

A cross-sectional survey will be conducted across all clusters beginning four weeks after the completion of the second year of MDA, during peak transmission. 200 participants will be selected by a two-step randomisation (household; individual) from within a "yolk" of villages in each cluster, purposively defined to capture sufficiently populated villages far from other clusters but logistically feasible to reach. Socio-demographic and GPS data will be collected alongside Dried Blood Spots (DBS) for varATS qPCR analysis, a technique capable of detecting 0.03-0.15 parasites/µL blood, 10 times the sensitivity of standard 18S rRNA qPCR.[45]

In November 2019, mean malaria 18S qPCR prevalence across the islands was 14.8% (95% CI: 14.5-14.9%) (unpublished). These results informed a conservative estimate of the co-efficient of variation of 0.46. 200 participants/cluster in 12 clusters/arm provides >80% power to detect a difference between arms if the primary outcome prevalence is 10% in the control arm and 5% in the intervention arm.

A mathematical model parameterised to simulate prevalence, seasonality, vector control and coverage, predicts qPCR prevalence of 9% (control) and 4% (intervention) after one year of MDA, and 7% (control) and 1% (intervention) after two (Figure 4).

Secondary

1. A survey, identical to the primary outcome survey, will be conducted after one year of MDA.
2. On the last day of every month, data on RDT-confirmed incident malaria cases will be recorded from each of the 10 health centres on included islands: age, sex, cluster, fever, RDT performed, RDT result, treatment provided.
3. Cohorts of 50 children aged 5-14 will be followed in 18 clusters. Households will be randomly selected from household-head lists of yolk villages. One child will be randomly selected to participate from each household, more if there are fewer than 50 eligible households. A new cohort will be selected in 2022. Participants will be visited 7-14 days after each round of MDA, as well as during the peak transmission surveys for a total of 8 timepoints. Age, sex, village, GPS and temperature will be recorded. Febrile children will be offered an RDT and treated if positive. All children

For peer review only - http://bmjopen.bmj.com/site/about/guidelines.xhtml

will be asked about intercurrent clinical malaria episodes, trial adherence and bed net usage. A DBS will be taken for molecular and serological analysis.

4. Assessed as for Outcomes 3 and 5.
5. Serological analysis will be performed on DBS taken during the cross-sectional and cohort surveys described above using a multiplex bead assay on the Luminex MAGPIX platform. The included antigens have antibodies which are associated with recent or long-term exposure to, and/or protection from, *Pf*: MSP1.19, AMA1, GLURP.R2, EBA175.RIII.V, EBA181.RIII.V, Etramp5.Ag1, Etramp4.Ag2, HSP40.Ag1, CSP, SBP1, Hyp2, GEXP18, Rh2.2030, Rh4.1 and Rh5.2.[46–48]
6. Assessed as for Outcome 5.
7. One yolk village will be randomly selected per cluster. Households will be randomly selected in these villages using household-head lists (10 households/cluster in year 1; 15/cluster in year 2). A CDC light trap will be placed inside each household overnight for three consecutive nights, 7 days after each round of MDA in year 1 (18 clusters) and 7 days after the third round of MDA in year 2 (24 clusters). There will be three further nights of trapping during the peak transmission survey. *Anopheles gambiae s.l.* will be identified morphologically.[49] Parity, estimating mosquito survival, will be done using the Detinova ovarian tracheation method.[50]
8. These mosquitoes will also be used to estimate cluster- and trial arm-level species composition (proportion of species present within *An. gambiae* complex), population density (number of mosquitoes/trap/night; a proxy for human biting rate) and sporozoite detection using CSP ELISA.[51] The Entomological Inoculation Rate (EIR), a proxy for human exposure to infectious mosquitoes and a key indicator of local transmission, will be estimated using these data.[52]
9. Anopheline mosquitoes will be reared from locally-caught larvae during the 2022 rainy season. Batches of 100 adult females will be tested for resistance to alpha-cypermethrin, permethrin and deltamethrin, with and without the synergist piperonyl butoxide, using CDC bottle bioassays and WHO test tubes at once, twice and five-times the discriminating dose.[53,54] Two hundred mosquitoes, including all resistant individuals, will undergo PCR species identification.[55]
10. Samples positive for *Pf* DNA will undergo further extraction from survey DBS, amplified by PCR[56] and sequenced using illumina-based technology to identify genetic mutations previously associated with antimalarial drug resistance, including polymorphisms in *pfkelch, pfmdr1, pfcrt* and *pfexo* genes, before being analysed using bioinformatic tools.[57] Selective whole genome amplification steps will be conducted on the initial DNA extract to increase the quantity of *Pf* DNA.
11. AE data will be collected actively by distributors during MDA and for 48 hours after, and passively by sensitising participants to report AEs. Health centre nurses will be trained to identify and report AEs, and ASCs will monitor births, deaths and miscarriages. Daily reports are made via supervisors to the trial manager for processing. Summaries will be reviewed at least annually by the Data Safety Monitoring Board (DSMB). Cross-sectional and cohort surveys include *post hoc* questions on AEs. All doses will be directly observed. *Loa loa* is not endemic to this region, greatly reducing the risk of IVM-associated encephalitis.
12. A population-based cross-sectional survey will be performed in all clusters during the 2023 dry season: 100 households will be randomly selected from household-head lists in each cluster yolk, and one child aged 2-10 invited to participate from each household. Multiple children will be invited if the sample size is not met. Skin will be

For peer review only - http://bmjopen.bmj.com/site/about/guidelines.xhtml

systematically examined to identify scabies, and impetigo in positive cases. Headlice examination and fine-tooth combing above a white cloth will occur over 10 minutes. Bedframes, mattresses, nets and adjacent walls will be inspected with torchlight for signs of bedbugs, or eggs, over ten minutes. A stool sample (5g) will be collected and stored in ethanol to undergo multiplex parasite PCR (STH, Strongyloides).[58–60] DBS will be collected for serological testing for LF, strongyloidiasis and STH.[61–63]

13. MDA distributors enter administration data into forms listing every householder, including absent members, and capture data on daily refusals, absences and exclusions. These forms generate a *de facto* census and can be used calculate monthly cluster-level coverage, absence, refusal and exclusion statistics.
14. Qualitative and quantitative analysis of MDA acceptability, feasibility and access will be outlined in a separate protocol.
15. Cost-effectiveness analysis will be detailed in a separate protocol.

Data Analysis Plan

All analysis will be intention-to-treat. Analysis will be performed using STATA software (StataCorp) and R.

Cluster-, arm-level and overall demographic characteristics will be presented using descriptive statistics, including age, sex, bed net use, household size, and village/cluster populations.

Primary Outcome

Trial arms will be compared using a t-test on cluster-level malaria qPCR prevalences. A risk difference, 95% CI and P-value will be presented. Analyses adjusting for age groups (<5, 5-14, 15 and above), bed net use and presence of a health centre will be presented for this and all secondary outcomes.

Secondary Outcomes

1. As for the primary outcome, using data from the 2021 survey.
2. Cluster-level incidence rates will be compared using a t-test. If the distribution is markedly skewed then the natural logarithm of each cluster-level summary will be taken. If some clusters have zero events, then 0.5 events will be added to each cluster. Rate differences, 95% CI and P-value will be presented.
3. As for Outcome 2, using RDT-confirmed incidence rates from the two annual cohorts of children.
4. As for Outcome 3, using qPCR-confirmed incidence rates. Serological analysis described in Outcome 5.
5. Cluster-level serological responses to malaria antigens will be presented as both continuous Median Fluorescent Intensity (MFI) data, a proxy for antibody titres, and binary seropositivity prevalence, with a seropositivity threshold of 3sd above mean malaria-naïve MFI responses. MFI will be compared between arms using mixed effect linear regression, with a random effect for cluster. Seropositivity will be compared in the same way as the primary outcome, including analysis by age-group. Cluster-level antigen-specific Seroconversion Rates (SCR) will be generated using serocatalytic models relying on seroprevalence, and age-seroprevalence plots will be generated by fitting data to a reversible catalytic conversion model using maximum likelihood methods. Trial arms' SCR will be compared using a t-test.[48]

For peer review only - http://bmjopen.bmj.com/site/about/guidelines.xhtml

6. As for Outcome 5, using the gSG6 antigen and not employing SCR.
7. Cluster-level anopheline parity will be compared between arms using a t-test. Adjustment will be made for species, MDA round, time relative to MDA date, temperature, humidity and rainfall. Parity difference, 95% CI and P-value will be presented.
8. The proportion of each species identified, and mosquito densities, will be presented by cluster and arm, and compared using chi-square and t-tests. Adjustment will be made for study month and year. Differences, 95% CIs and P-values will be presented. If the distribution of densities is markedly skewed, a log transformation will be applied. SR will be compared between arms using a t-test on cluster-level SR with adjustments and covariates as described for parity analysis above. Risk difference, 95% CI and P-value will be presented. EIR will be compared with a t-test on cluster level summaries.
9. Species composition and level of resistance will be presented using descriptive statistics. Dose-response curves will be generated for each insecticide and probit regression analysis will be performed on these data using maximum likelihood or least squares methods.
10. The number of alleles associated with antimalarial resistance will be compared over time, before and after MDA. However, appropriate statistical tests will need to be selected depending on the allele sample size before and after MDA.
11. Descriptive statistics will be presented on the number, nature, severity and relatedness of all reported AE, with additional details for serious AE. Cluster-level and age-group data will be presented. Rates will be compared between arms.
12. As for the primary outcome, and Outcome 5.
13. Cluster-, arm-level and overall coverage will be presented for each month and year of intervention, and for the trial overall. The denominator for these proportions will be the total number of people recorded on MDA Administration Records by distributors in each cluster, whether receiving MDA or not. Refusals, exclusions and absences will be presented similarly.
14. Qualitative and quantitative analysis of MDA acceptability, feasibility and access will be outlined in a separate protocol.
15. Cost-effectiveness analysis will be detailed in a separate protocol.

Patient and Public Involvement

Qualitative studies report that this population almost unanimously consider malaria to be a significant problem in their homes and the region, that additional malaria control measures would be welcome, and that MDA is acceptable,[35,64] all of which informed MATAMAL's design.

All field assistants and the deputy trial manager are local residents and MDA will be delivered by ASCs within their own communities. Methods were finalised in discussion with these stakeholders, the Regional Health Directorate and MINSAP. There will be monthly feedback sessions with ASCs and staff during MDA and surveys. Trial outcomes, social science, public engagement and qualitative work will be presented to stakeholders and communities using plans developed with the contributors themselves.

For peer review only - http://bmjopen.bmj.com/site/about/guidelines.xhtml

TRIAL STATUS

All necessary approvals are in place. A baseline malaria prevalence survey was completed in November 2019. Two years of MDA has successfully been delivered across all clusters, alongside two annual cohort surveys and entomological sample collection. Two annual peak transmission surveys have been completed. Qualitative and social science work has taken place throughout. The NTD survey is due to begin recruitment in March 2023.

ETHICS AND DISSEMINATION

This protocol development was written in line with SPIRIT guidelines.[65,66] MATAMAL will be reported according to CONSORT guidance.[67] It is a collaboration between LSHTM, Medical Research Council The Gambia and MINSAP. Ethical approval has been obtained from LSHTM Research Ethics Committee (UK) (19156) and CNES (Guinea-Bissau) (084/CNES/INASA/2020), with additional regulatory approvals from The Gambia. The DSMB will oversee trial safety. This work abides by the Declaration of Helsinki[68] and Good Clinical Practice.[69]

Electronic forms are encrypted immediately upon submission to a HERA-compliant secure server at LSHTM. Paper forms will be kept in limited-access locked storage at the site office. Data will be de-identified, except for consent and MDA record forms. All data will be held for a minimum of 7 years, including in LSHTM electronic repositories, whence access to de-identified data can be requested.

Results will be published in peer-review journals, presented at conferences and to local collaborators. Field staff, nurses and ASCs will disseminate findings to participating communities in appropriate and accessible formats.

REFERENCES

1. Bhatt S, Weiss DJ, Cameron E, Bisanzio D, Mappin B, Dalrymple U, et al. The effect of malaria control on Plasmodium falciparum in Africa between 2000 and 2015. Nature [Internet]. 2015 Oct 8 [cited 2019 Sep 23];526(7572):207–11. Available from: http://www.ncbi.nlm.nih.gov/pubmed/26375008
2. Barnes KG, Weedall GD, Ndula M, Irving H, Mzihalowa T, Hemingway J, et al. Genomic Footprints of Selective Sweeps from Metabolic Resistance to Pyrethroids in African Malaria Vectors Are Driven by Scale up of Insecticide-Based Vector Control. PLOS Genet [Internet]. 2017 Feb 1 [cited 2022 Oct 4];13(2):e1006539. Available from: https://journals.plos.org/plosgenetics/article?id=10.1371/journal.pgen.1006539
3. Griffin JT, Hollingsworth TD, Okell LC, Churcher TS, White M, Hinsley W, et al. Reducing Plasmodium falciparum malaria transmission in Africa: a model-based evaluation of intervention strategies. PLoS Med [Internet]. 2010 Aug [cited 2022 Oct 4];7(8). Available from: https://pubmed.ncbi.nlm.nih.gov/20711482/
4. Global Malaria Programme. Malaria Policy Advisory Committee meeting report [Internet]. Geneva; 2016 Mar [cited 2022 Oct 4]. Available from: https://www.who.int/publications/i/item/WHO-HTM-GMP-MPAC-2016.3
5. Bousema T, Okell L, Felger I, Drakeley C. Asymptomatic malaria infections: detectability, transmissibility and public health relevance. Nat Rev Microbiol [Internet]. 2014 Dec 18 [cited 2022 Jan 11];12(12):833–40. Available from: https://pubmed.ncbi.nlm.nih.gov/25329408/
6. Shah MP, Hwang J, Choi L, Lindblade KA, Kachur SP, Desai M. Mass drug

For peer review only - http://bmjopen.bmj.com/site/about/guidelines.xhtml

administration for malaria. Cochrane Database Syst Rev [Internet]. 2021 Sep 29 [cited 2022 Oct 4];2021(9). Available from: /pmc/articles/PMC8479726/

7. Brady OJ, Slater HC, Pemberton-Ross P, Wenger E, Maude RJ, Ghani AC, et al. Role of mass drug administration in elimination of Plasmodium falciparum malaria: a consensus modelling study. Lancet Glob Heal [Internet]. 2017 Jul 1 [cited 2022 Oct 4];5(7):e680–7. Available from: https://pubmed.ncbi.nlm.nih.gov/28566213/
8. Zani B, Gathu M, Donegan S, Olliaro PL, Sinclair D. Dihydroartemisinin-piperaquine for treating uncomplicated Plasmodium falciparum malaria. Cochrane database Syst Rev [Internet]. 2014 Jan 20 [cited 2022 Oct 5];2014(1). Available from: https://pubmed.ncbi.nlm.nih.gov/24443033/
9. Keating GM. Dihydroartemisinin/Piperaquine: a review of its use in the treatment of uncomplicated Plasmodium falciparum malaria. Drugs [Internet]. 2012 [cited 2022 Oct 5];72(7):937–61. Available from: https://pubmed.ncbi.nlm.nih.gov/22515619/
10. Adjei A, Narh-Bana S, Amu A, Kukula V, Nagai RA, Owusu-Agyei S, et al. Treatment outcomes in a safety observational study of dihydroartemisinin/piperaquine (Eurartesim®) in the treatment of uncomplicated malaria at public health facilities in four African countries. Malar J [Internet]. 2016 Jan 27 [cited 2022 Oct 5];15(1):1–10. Available from: https://malariajournal.biomedcentral.com/articles/10.1186/s12936-016-1099-7
11. Pekyi D, Ampromfi AA, Tinto H, Traoré-Coulibaly M, Tahita MC, Valéa I, et al. Four artemisinin-based treatments in African pregnant women with malaria. N Engl J Med [Internet]. 2016 Mar 10 [cited 2022 Oct 5];28(3):139–49. Available from: https://www.nejm.org/doi/full/10.1056/NEJMoa1508606
12. Pfeil J, Borrmann S, Bassat Q, Mulenga M, Talisuna A, Tozan Y. An Economic Evaluation of the Posttreatment Prophylactic Effect of Dihydroartemisinin-Piperaquine Versus Artemether-Lumefantrine for First-Line Treatment of Plasmodium falciparum Malaria Across Different Transmission Settings in Africa. Am J Trop Med Hyg [Internet]. 2015 Nov 4 [cited 2022 Oct 5];93(5):961–6. Available from: https://pubmed.ncbi.nlm.nih.gov/26240155/
13. Eisele TP, Silumbe K, Finn T, Chalwe V, Kamuliwo M, Hamainza B, et al. Assessing the effectiveness of household-level focal mass drug administration and community-wide mass drug administration for reducing malaria parasite infection prevalence and incidence in Southern Province, Zambia: Study protocol for a community randomized controlled trial. Trials [Internet]. 2015 Aug 13 [cited 2022 Oct 5];16(1):1–13. Available from: https://trialsjournal.biomedcentral.com/articles/10.1186/s13063-015-0862-3
14. Zani B, Gathu M, Donegan S, Olliaro PL, Sinclair D. Dihydroartemisinin-piperaquine for treating uncomplicated Plasmodium falciparum malaria. Cochrane database Syst Rev. 2014 Jan;2014(1).
15. Eisele TP, Bennett A, Silumbe K, Finn TP, Chalwe V, Kamuliwo M, et al. Short-term Impact of Mass Drug Administration With Dihydroartemisinin Plus Piperaquine on Malaria in Southern Province Zambia: A Cluster-Randomized Controlled Trial. J Infect Dis [Internet]. 2016 Dec 12 [cited 2022 Oct 5];214(12):1831. Available from: /pmc/articles/PMC5142084/
16. Mwesigwa J, Achan J, Affara M, Wathuo M, Worwui A, Mohammed NI, et al. Mass Drug Administration With Dihydroartemisinin-piperaquine and Malaria Transmission Dynamics in The Gambia: A Prospective Cohort Study. Clin Infect Dis [Internet]. 2019 Jul 2 [cited 2022 Oct 4];69(2):278–86. Available from: https://pubmed.ncbi.nlm.nih.gov/30304511/
17. Chaccour C, Hammann F, Rabinovich NR. Ivermectin to reduce malaria transmission I. Pharmacokinetic and pharmacodynamic considerations regarding efficacy and safety. Malar J [Internet]. 2017 [cited 2019 Sep 6];16. Available from:

For peer review only - http://bmjopen.bmj.com/site/about/guidelines.xhtml

https://www.ncbi.nlm.nih.gov/pmc/articles/PMC5402169/
18. Awadzi K, Opoku NO, Addy ET, Quartey BT. The chemotherapy of onchocerciasis. XIX: The clinical and laboratory tolerance of high dose ivermectin - PubMed. Trop Med Parasitol [Internet]. 1995 Jun [cited 2022 Oct 4];46(2):131–7. Available from: https://pubmed.ncbi.nlm.nih.gov/8525285/
19. Guzzo CA, Furtek CI, Porras AG, Chen C, Tipping R, Clineschmidt CM, et al. Safety, tolerability, and pharmacokinetics of escalating high doses of ivermectin in healthy adult subjects. J Clin Pharmacol [Internet]. 2002 Oct [cited 2022 Oct 4];42(10):1122–33. Available from: https://pubmed.ncbi.nlm.nih.gov/12362927/
20. Kamgno J, Gardon J, Gardon-Wendel N, Demanga-Ngangue A, Duke BOL, Boussinesq M. Adverse systemic reactions to treatment of onchocerciasis with ivermectin at normal and high doses given annually or three-monthly. Trans R Soc Trop Med Hyg [Internet]. 2004 Aug [cited 2022 Oct 4];98(8):496–504. Available from: https://pubmed.ncbi.nlm.nih.gov/15186939/
21. Fobi G, Gardon J, Kamgno J, Aimard-Favennec L, Lafleur C, Gardon-Wendel N, et al. A randomized, double-blind, controlled trial of the effects of ivermectin at normal and high doses, given annually or three-monthly, against Onchocerca volvulus: ophthalmological results. Trans R Soc Trop Med Hyg [Internet]. 2005 Apr [cited 2022 Oct 4];99(4):279–89. Available from: https://pubmed.ncbi.nlm.nih.gov/15708387/
22. WHO. Malaria Policy Advisory Committee to the WHO: Conclusions and recommendations of eighth biannual meeting (September 2015). Malar J [Internet]. 2016 Feb 24 [cited 2022 Oct 5];15(1):1–12. Available from: https://malariajournal.biomedcentral.com/articles/10.1186/s12936-016-1169-x
23. Alout H, Krajacich BJ, Meyers JI, Grubaugh ND, Brackney DE, Kobylinski KC, et al. Evaluation of ivermectin mass drug administration for malaria transmission control across different West African environments. Malar J. 2014 Nov 3;13(1).
24. Slater HC, Walker PGT, Bousema T, Okell LC, Ghani AC. The Potential Impact of Adding Ivermectin to a Mass Treatment Intervention to Reduce Malaria Transmission: A Modelling Study. J Infect Dis [Internet]. 2014 Dec 15 [cited 2019 Oct 3];210(12):1972–80. Available from: http://www.ncbi.nlm.nih.gov/pubmed/24951826
25. Smit MR, Ochomo EO, Aljayyoussi G, Kwambai TK, Abong'o BO, Chen T, et al. Safety and mosquitocidal efficacy of high-dose ivermectin when co-administered with dihydroartemisinin-piperaquine in Kenyan adults with uncomplicated malaria (IVERMAL): a randomised, double-blind, placebo-controlled trial. Lancet Infect Dis [Internet]. 2018 Jun 1 [cited 2019 Sep 2];18(6):615–26. Available from: https://www.sciencedirect.com/science/article/pii/S1473309918301634
26. Smit MR, Ochomo E, Aljayyoussi G, Kwambai T, Abong'o B, Bayoh N, et al. Efficacy and Safety of High-Dose Ivermectin for Reducing Malaria Transmission (IVERMAL): Protocol for a Double-Blind, Randomized, Placebo-Controlled, Dose-Finding Trial in Western Kenya. JMIR Res Protoc [Internet]. 2016 Nov 17 [cited 2022 Oct 4];5(4):e213. Available from: https://pubmed.ncbi.nlm.nih.gov/27856406/
27. Abramowicz M, Zuccotti G. Drugs for Head Lice. JAMA [Internet]. 2017 May 16 [cited 2022 Oct 4];317(19):2010–1. Available from: https://pubmed.ncbi.nlm.nih.gov/28510677/
28. Ouedraogo AL, Bastiaens GJH, Tiono AB, Guelbeogo WM, Kobylinski KC, Ouedraogo A, et al. Efficacy and Safety of the Mosquitocidal Drug Ivermectin to Prevent Malaria Transmission After Treatment: A Double-Blind, Randomized, Clinical Trial. Clin Infect Dis [Internet]. 2015 Feb 1 [cited 2019 Sep 6];60(3):357–65. Available from: https://academic.oup.com/cid/article-lookup/doi/10.1093/cid/ciu797
29. Foy BD, Alout H, Seaman JA, Rao S, Magalhaes T, Wade M, et al. Efficacy and risk of

For peer review only - http://bmjopen.bmj.com/site/about/guidelines.xhtml

harms of repeat ivermectin mass drug administrations for control of malaria (RIMDAMAL): a cluster-randomised trial. Lancet (London, England) [Internet]. 2019 Apr 13 [cited 2019 Jul 13];393(10180):1517–26. Available from: http://www.ncbi.nlm.nih.gov/pubmed/30878222
30. Dabira ED, Soumare HM, Conteh B, Ceesay F, Ndiath MO, Bradley J, et al. Mass drug administration of ivermectin and dihydroartemisinin–piperaquine against malaria in settings with high coverage of standard control interventions: a cluster-randomised controlled trial in The Gambia. Lancet Infect Dis. 2021 Dec;0(0).
31. Kobylinski KC, Jittamala P, Hanboonkunupakarn B, Pukrittayakamee S, Pantuwatana K, Phasomkusolsil S, et al. Safety, Pharmacokinetics, and Mosquito-Lethal Effects of Ivermectin in Combination With Dihydroartemisinin-Piperaquine and Primaquine in Healthy Adult Thai Subjects. Clin Pharmacol Ther [Internet]. 2020 May 1 [cited 2022 Oct 5];107(5):1221–30. Available from: https://pubmed.ncbi.nlm.nih.gov/31697848/
32. Bradley J, Moulton LH, Hayes R. Analysis of the RIMDAMAL trial. Lancet [Internet]. 2019 Sep 21 [cited 2023 Jan 16];394(10203):1005–6. Available from: http://www.thelancet.com/article/S0140673619316630/fulltext
33. Metroblue. Observed historical climate & weather data for Bubaque - meteoblue [Internet]. Metroblue. 2022 [cited 2022 Feb 4]. Available from: https://www.meteoblue.com/en/weather/historyclimate/climateobserved/bubaque_guinea-bissau_2374583
34. Durrans S, Last A, Boiro H, Goncalves A, Mabey D, Greenland K. "Moving like birds": A qualitative study of population mobility and health implications in the Bijagós Islands, Guinea Bissau. Soc Sci Med [Internet]. 2019 Jun 1 [cited 2019 Oct 1];230:204–13. Available from: https://www.sciencedirect.com/science/article/pii/S0277953619301613
35. Hutchins H, Power G, Ant T, Teixeira da Silva E, Goncalves A, Rodrigues A, et al. A survey of knowledge, attitudes and practices regarding malaria and bed nets on Bubaque Island, Guinea-Bissau. Malar J [Internet]. 2020 Dec 1 [cited 2022 Jan 31];19(1):1–15. Available from: https://malariajournal.biomedcentral.com/articles/10.1186/s12936-020-03469-1
36. Ant T, Foley E, Tytheridge S, Johnston C, Goncalves A, Ceesay S, et al. A survey of Anopheles species composition and insecticide resistance on the island of Bubaque, Bijagos Archipelago, Guinea-Bissau. Malar J [Internet]. 2020 Jan 15 [cited 2022 Feb 1];19(1):1–9. Available from: https://malariajournal.biomedcentral.com/articles/10.1186/s12936-020-3115-1
37. Farrant O, Marlais T, Houghton J, Goncalves A, Teixeira da Silva Cassama E, Cabral MG, et al. Prevalence, risk factors and health consequences of soil-transmitted helminth infection on the Bijagos Islands, Guinea Bissau: A community-wide cross-sectional study. PLoS Negl Trop Dis [Internet]. 2020 Dec 1 [cited 2023 Jan 20];14(12):e0008938. Available from: /pmc/articles/PMC7773412/
38. Last AR, Burr SE, Harding-Esch E, Cassama E, Nabicassa M, Roberts CH, et al. The impact of a single round of community mass treatment with azithromycin on disease severity and ocular Chlamydia trachomatis load in treatment-naïve trachoma-endemic island communities in West Africa. Parasit Vectors [Internet]. 2017 Dec 28 [cited 2022 Oct 4];10(1). Available from: https://pubmed.ncbi.nlm.nih.gov/29282126/
39. Jones RT, Pretorius E, Ant TH, Bradley J, Last A, Logan JG. The use of islands and cluster-randomized trials to investigate vector control interventions: a case study on the Bijagós archipelago, Guinea-Bissau. Philos Trans R Soc B Biol Sci [Internet]. 2021 Feb 2 [cited 2023 Jan 24];376(1818). Available from: /pmc/articles/PMC7776941/
40. de Souza DK, Thomas R, Bradley J, Leyrat C, Boakye DA, Okebe J. Ivermectin

For peer review only - http://bmjopen.bmj.com/site/about/guidelines.xhtml

treatment in humans for reducing malaria transmission. Cochrane Database Syst Rev [Internet]. 2021 Jun 29 [cited 2023 Jan 23];2021(6). Available from: https://www.cochranelibrary.com/cdsr/doi/10.1002/14651858.CD013117.pub2/full

41. Hayes RJ, Moulton LH. Cluster randomised trials. 2nd ed. Chapman and Hall/CRC; 2017.
42. INEP Guine-Bissau. Guinea-Bissau 2009 Census [Internet]. Guinea-Bissau Data Portal. 2009 [cited 2020 Apr 16]. Available from: https://guinebissau.opendataforafrica.org/amthtjd/guinea-bissau-2009-census
43. Hung TY, Davis TME, Ilett KF, Karunajeewa H, Hewitt S, Denis MB, et al. Population pharmacokinetics of piperaquine in adults and children with uncomplicated falciparum or vivax malaria. Br J Clin Pharmacol [Internet]. 2004 Mar [cited 2022 Oct 13];57(3):253. Available from: /pmc/articles/PMC1884452/
44. Chaccour C, Lines J, Whitty CJM. Effect of Ivermectin on Anopheles gambiae Mosquitoes Fed on Humans: The Potential of Oral Insecticides in Malaria Control . J Infect Dis. 2010 Jul;202(1):113–6.
45. Hofmann N, Mwingira F, Shekalaghe S, Robinson LJ, Mueller I, Felger I. Ultra-Sensitive Detection of Plasmodium falciparum by Amplification of Multi-Copy Subtelomeric Targets. PLOS Med [Internet]. 2015 Mar 1 [cited 2023 Jan 21];12(3):e1001788. Available from: https://journals.plos.org/plosmedicine/article?id=10.1371/journal.pmed.1001788
46. Wu L, Hall T, Ssewanyana I, Oulton T, Patterson C, Vasileva H, et al. Optimisation and standardisation of a multiplex immunoassay of diverse Plasmodium falciparum antigens to assess changes in malaria transmission using sero-epidemiology. Wellcome Open Res. 2020 Apr 23;4:26.
47. Helb DA, Tetteh KKA, Felgner PL, Skinner J, Hubbard A, Arinaitwe E, et al. Novel serologic biomarkers provide accurate estimates of recent Plasmodium falciparum exposure for individuals and communities. Proc Natl Acad Sci U S A [Internet]. 2015 Aug 11 [cited 2022 Jan 7];112(32):E4438–47. Available from: /pmc/articles/PMC4538641/
48. Drakeley CJ, Corran PH, Coleman PG, Tongren JE, McDonald SLR, Carneiro I, et al. Estimating medium- and long-term trends in malaria transmission by using serological markers of malaria exposure. Proc Natl Acad Sci [Internet]. 2005 Apr 5 [cited 2022 Jan 10];102(14):5108–13. Available from: https://www.pnas.org/content/102/14/5108
49. Coetzee M. Key to the females of Afrotropical Anopheles mosquitoes (Diptera: Culicidae). Malar J [Internet]. 2020 Feb 13 [cited 2023 Jan 24];19(1):1–20. Available from: https://malariajournal.biomedcentral.com/articles/10.1186/s12936-020-3144-9
50. Detinova T. Age-grouping methods in diptera of medical importance [Internet]. Geneva: WHO; 1962 [cited 2023 Jan 24]. 1–213 p. Available from: https://apps.who.int/iris/bitstream/handle/10665/41724/WHO_MONO_47_(part1).pdf;sequence=1
51. Wirtz RA, Zavala F, Charoenvit Y, Campbell GH, Burkot TR, Schneider I, et al. Comparative testing of monoclonal antibodies against Plasmodium falciparum sporozoites for ELISA development. Bull World Health Organ [Internet]. 1987 [cited 2023 Jan 20];65(1):39. Available from: /pmc/articles/PMC2490858/?report=abstract
52. Kilama M, Smith DL, Hutchinson R, Kigozi R, Yeka A, Lavoy G, et al. Estimating the annual entomological inoculation rate for Plasmodium falciparum transmitted by Anopheles gambiae s.l. using three sampling methods in three sites in Uganda. Malar J [Internet]. 2014 Mar 21 [cited 2023 Jan 24];13(1). Available from: https://pubmed.ncbi.nlm.nih.gov/24656206/

For peer review only - http://bmjopen.bmj.com/site/about/guidelines.xhtml

53. Brogdon W, Chan A. Guideline for Evaluating Insecticide Resistance in Vectors Using the CDC Bottle Bioassay [Internet]. [cited 2023 Jan 20]. p. 1–28. Available from: https://www.cdc.gov/malaria/resources/pdf/fsp/ir_manual/ir_cdc_bioassay_en.pdf
54. WHO. Standard operating proceudre for testing insecticide susceptibility of adult mosquitoes in WHO test tubes [Internet]. Geneva; 2022. Available from: https://www.who.int/publications/i/item/9789240043831
55. Fanello C, Santolamazza F, Della Torre A. Simultaneous identification of species and molecular forms of the Anopheles gambiae complex by PCR-RFLP. Med Vet Entomol [Internet]. 2002 Dec 1 [cited 2023 Jan 20];16(4):461–4. Available from: https://onlinelibrary.wiley.com/doi/full/10.1046/j.1365-2915.2002.00393.x
56. Oyola SO, Ariani C V., Hamilton WL, Kekre M, Amenga-Etego LN, Ghansah A, et al. Whole genome sequencing of Plasmodium falciparum from dried blood spots using selective whole genome amplification. Malar J [Internet]. 2016 Dec 20 [cited 2023 Jan 23];15(1):1–12. Available from: https://malariajournal.biomedcentral.com/articles/10.1186/s12936-016-1641-7
57. Nag S, Dalgaard MD, Kofoed PE, Ursing J, Crespo M, Andersen LOB, et al. High throughput resistance profiling of Plasmodium falciparum infections based on custom dual indexing and Illumina next generation sequencing-technology. Sci Rep [Internet]. 2017 Dec 1 [cited 2023 Jan 23];7(1). Available from: /pmc/articles/PMC5445084/
58. Llewellyn S, Inpankaew T, Nery SV, Gray DJ, Verweij JJ, Clements ACA, et al. Application of a Multiplex Quantitative PCR to Assess Prevalence and Intensity Of Intestinal Parasite Infections in a Controlled Clinical Trial. Bethony JM, editor. PLoS Negl Trop Dis [Internet]. 2016 Jan 28 [cited 2019 Sep 23];10(1):e0004380. Available from: https://dx.plos.org/10.1371/journal.pntd.0004380
59. Basuni M, Rahumatullah A, Miswan N, Ahmad M, Noordin R, Verweij JJ, et al. A Pentaplex Real-Time Polymerase Chain Reaction Assay for Detection of Four Species of Soil-Transmitted Helminths. Am J Trop Med Hyg [Internet]. 2011 Feb 4 [cited 2019 Sep 23];84(2):338–43. Available from: http://www.ncbi.nlm.nih.gov/pubmed/21292911
60. Verweij JJ, Canales M, Polman K, Ziem J, Brienen EAT, Polderman AM, et al. Molecular diagnosis of Strongyloides stercoralis in faecal samples using real-time PCR. Trans R Soc Trop Med Hyg [Internet]. 2009 Apr [cited 2023 Jan 21];103(4):342–6. Available from: https://pubmed.ncbi.nlm.nih.gov/19195671/
61. Pastor AF, Silva MR, dos Santos WJT, Rego T, Brandão E, de-Melo-Neto OP, et al. Recombinant antigens used as diagnostic tools for lymphatic filariasis. Parasites and Vectors [Internet]. 2021 Dec 1 [cited 2023 Jan 21];14(1):1–14. Available from: https://parasitesandvectors.biomedcentral.com/articles/10.1186/s13071-021-04980-3
62. Dana D, Vlaminck J, Ayana M, Tadege B, Mekonnen Z, Geldhof P, et al. Evaluation of copromicroscopy and serology to measure the exposure to Ascaris infections across age groups and to assess the impact of 3 years of biannual mass drug administration in Jimma Town, Ethiopia. Gilleard JS, editor. PLoS Negl Trop Dis [Internet]. 2020 Apr 13 [cited 2020 Jun 3];14(4):e0008037. Available from: https://dx.plos.org/10.1371/journal.pntd.0008037
63. Mounsey K, Kearns T, Rampton M, Llewellyn S, King M, Holt D, et al. Use of dried blood spots to define antibody response to the Strongyloides stercoralis recombinant antigen NIE. Acta Trop. 2014 Oct 1;138:78–82.
64. Thompson K, Hutchins H, Baio A, Cassama E, Nabicassa M, Bailey R, et al. Health Beliefs and Perceptions of Trachoma in Communities on the Bijagos Archipelago of Guinea Bissau. Ophthalmic Epidemiol [Internet]. 2015 May 4 [cited 2018 Mar

For peer review only - http://bmjopen.bmj.com/site/about/guidelines.xhtml

10];22(3):190–9. Available from: http://www.tandfonline.com/doi/full/10.3109/09286586.2015.1036889
65. Standard Protocol Items: Recommendations for Interventional Trials. SPIRIT - Guidance for Clinical Trial Protocols [Internet]. 2013 [cited 2022 Sep 23]. Available from: https://www.spirit-statement.org/
66. Chan A-W, Tetzlaff J, Gotzsche P, Altman D, Mann H, Berlin J, et al. SPIRIT 2013 Explanation and Elaboration: Guidance for protocols of clinical trials. BMJ [Internet]. 2013 [cited 2023 Jan 28];346(e7586). Available from: https://www.goodreports.org/reporting-checklists/spirit/
67. CONSORT Group. CONSORT Statement [Internet]. 2010 [cited 2022 Sep 23]. Available from: https://www.consort-statement.org/
68. WMA Declaration of Helsinki – Ethical Principles for Medical Research Involving Human Subjects – WMA – The World Medical Association [Internet]. [cited 2020 Apr 14]. Available from: https://www.wma.net/policies-post/wma-declaration-of-helsinki-ethical-principles-for-medical-research-involving-human-subjects/
69. ICH. ICH GCP - ICH harmonised guideline integrated addendum to ICH E6(R1): Guideline for Good Clinical Practice ICH E6(R2) ICH Consensus Guideline [Internet]. Good Clinical Practice Network. 2020 [cited 2020 Jun 25]. Available from: https://ichgcp.net/
70. Public Domain. Map of Guinea-Bissau. Commons Wikimedia.

AUTHORS' CONTRIBUTIONS

AL, the principal investigator, conceived the initial design of the study, secured funding and contributed significantly to writing this protocol. HH, the trial manager, runs all field operations, consulted on field methods and generated this manuscript. EP, RTJ and JGL drafted the entomology methods. JB provided statistical expertise. HS modelled the trial's anticipated impact. CD and HV provided serological expertise. ETS, PD, CM, AR and JN, our local collaborators, provided extensive information on local context and logistics. UD and DM supervised the writing of this protocol and provided expertise on trial conduct.

London School of Hygiene & Tropical Medicine (Keppel Street, London, WC1E 7HT, UK. +442076368636) is the trial sponsor. The sponsor and funders had no role in trial design, management or analysis.

FUNDING STATEMENT

This work was supported by a Joint Global Health Trials award (NIHR, MRC, Wellcome, FCDO), funder reference MR/S005013/1.

COMPETING INTERESTS

JGL declares he is founder/CEO of Arctech Innovation, a company which aims to design mosquito lures and malaria diagnostics.

ACKNOWLEDGEMENTS

We wish to express our sincere thanks to the health workers and residents of the Bijagos Archipelago, whose patience and support is the foundation of this trial.

For peer review only - http://bmjopen.bmj.com/site/about/guidelines.xhtml

FIGURE LEGENDS

Figure 1: Map of Guinea-Bissau, showing the Bijagos Archipelago off the south-west coast.[70]

Figure 2: Map of the Bijagos Archipelago. MATAMAL clusters marked by stars and separated by lines where sharing a landmass.

Figure 3: Design elements of the MATAMAL clinical trial.

Figure 4: Graph showing modelled qPCR prevalence of malaria over time, assuming 70% coverage and 75% dihydroartemisinin-piperaquine (DP) efficacy. Green dots: Mass Drug Administration (MDA) rounds. Green bars: survey periods. Dashed line: no intervention. Blue line: MDA with DP only. Pink line: MDA with DP and ivermectin at 300µg/Kg.

For peer review only

For peer review only - http://bmjopen.bmj.com/site/about/guidelines.xhtml

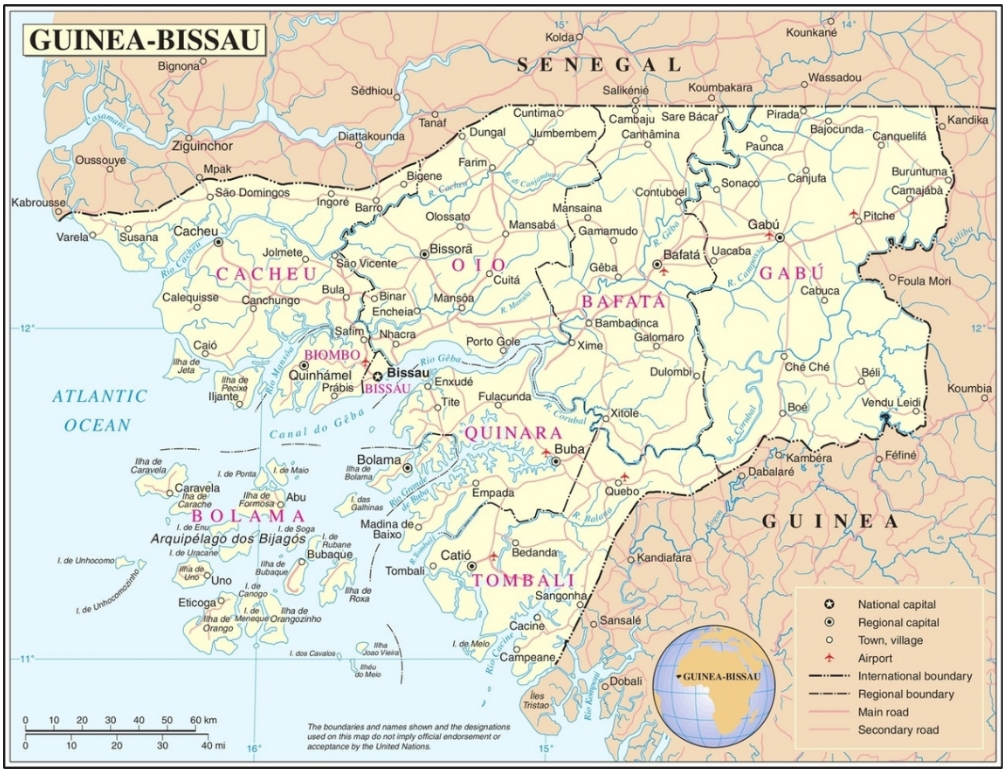

Figure 1: Map of Guinea-Bissau, showing the Bijagos Archipelago off the south-west coast.[69]

102x78mm (300 x 300 DPI)

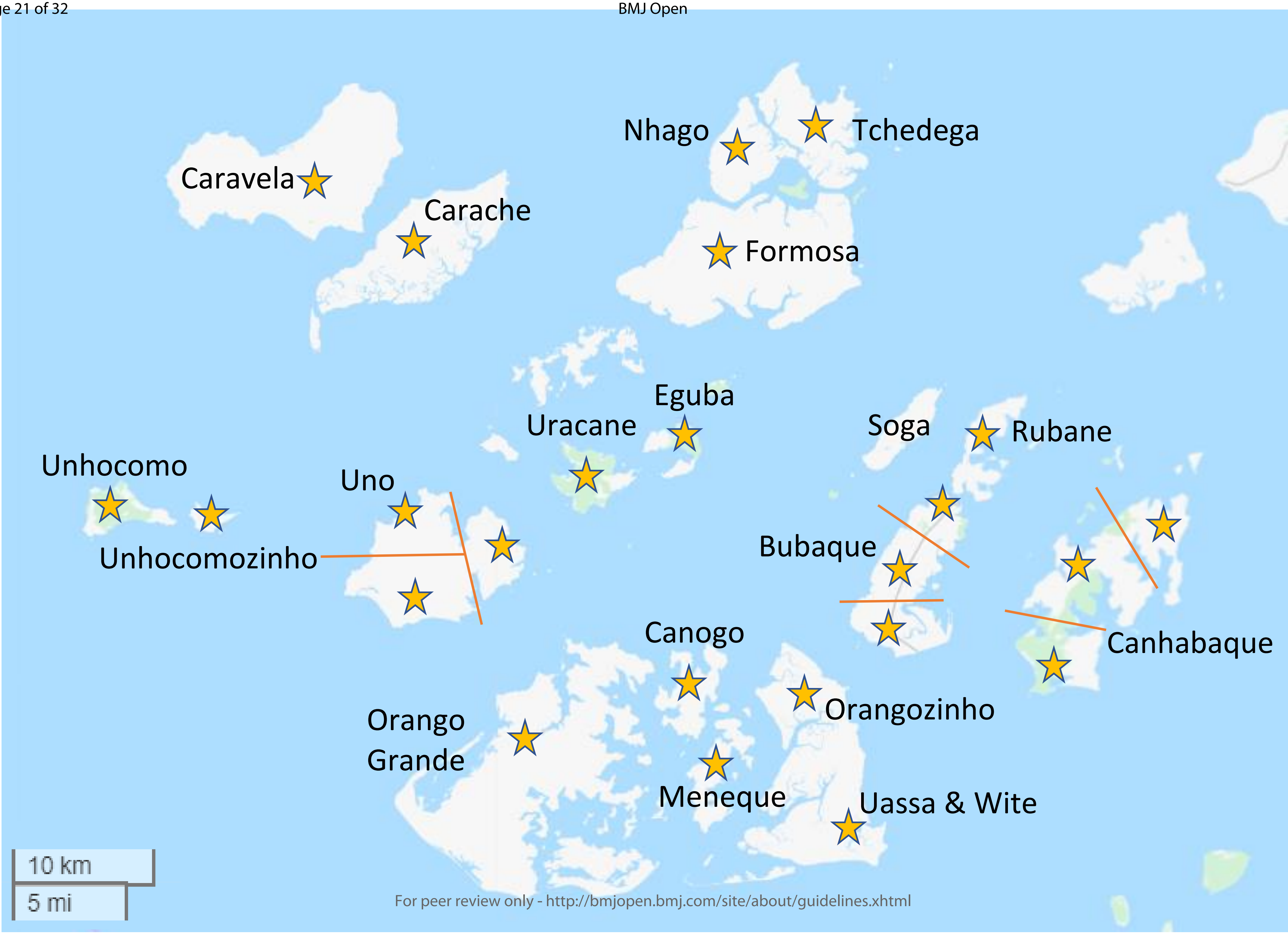
Caravela
Carache
Nhago
Tchedega
Formosa
Eguba
Uracane
Soga
Rubane
Unhocomo
Uno
Unhocomozinho
Bubaque
Canhabaque
Canogo
Orangozinho
Orango
Grande
Meneque
Uassa & Wite
10 km
5 mi

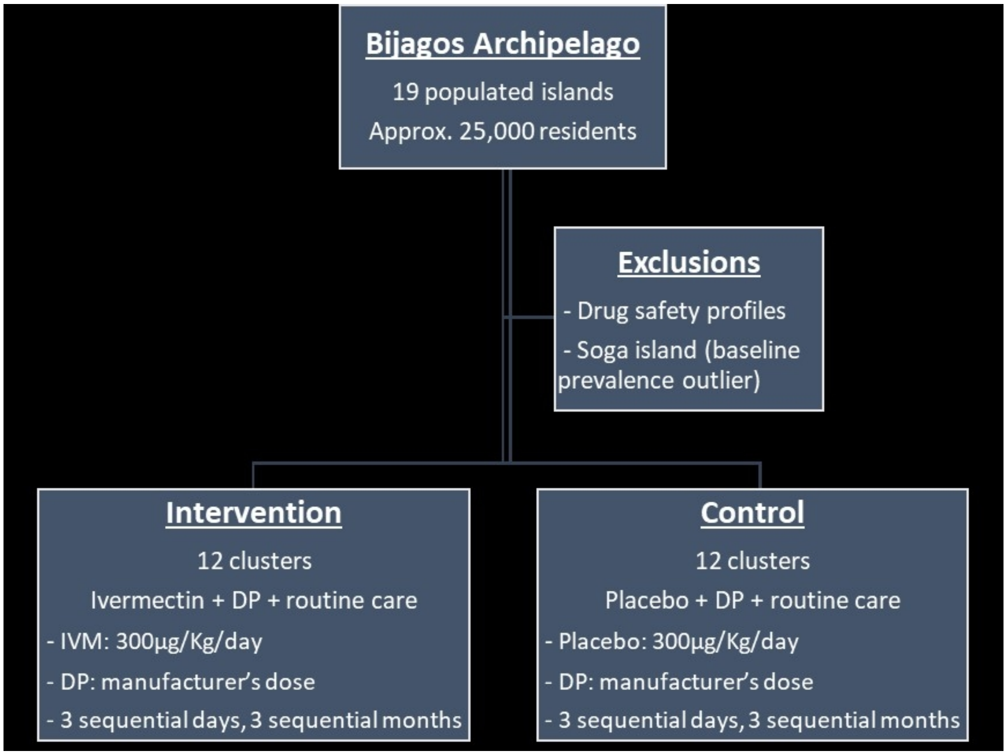

Figure 3: Design elements of the MATAMAL clinical trial.

146x109mm (150 x 150 DPI)

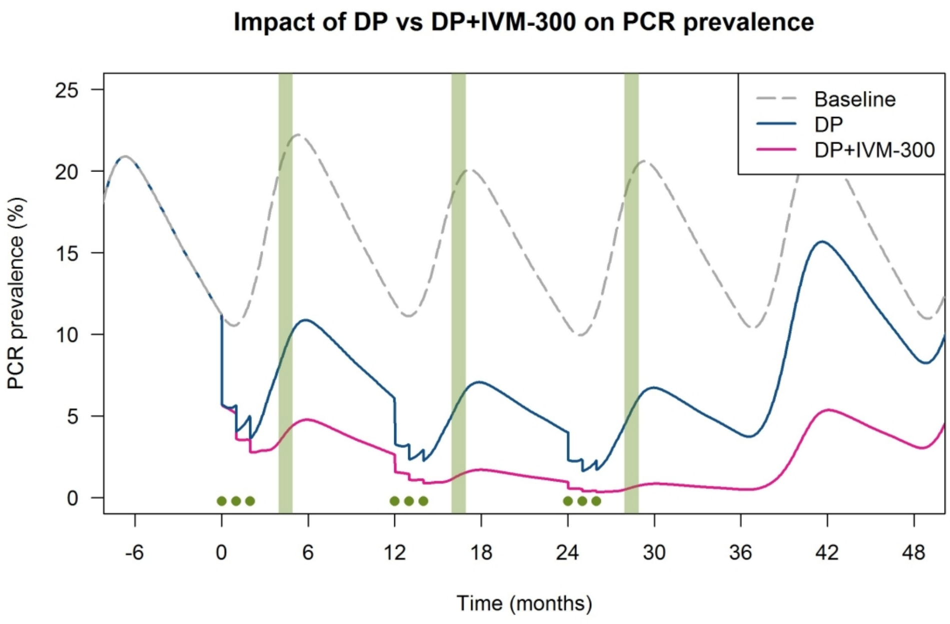

Figure 4: Graph showing modelled qPCR prevalence of malaria over time, assuming 70% coverage and 75% dihydroartemisinin-piperaquine (DP) efficacy. Green dots: Mass Drug Administration (MDA) rounds. Green bars: survey periods. Dashed line: no intervention. Blue line: MDA with DP only. Pink line: MDA with DP and ivermectin at 300μg/Kg.

199x134mm (300 x 300 DPI)

Reporting checklist for protocol of a clinical trial.

Based on the SPIRIT guidelines.

Instructions to authors

Complete this checklist by entering the page numbers from your manuscript where readers will find each of the items listed below.

Your article may not currently address all the items on the checklist. Please modify your text to include the missing information. If you are certain that an item does not apply, please write "n/a" and provide a short explanation.

Upload your completed checklist as an extra file when you submit to a journal.

In your methods section, say that you used the SPIRITreporting guidelines, and cite them as:

Chan A-W, Tetzlaff JM, Gøtzsche PC, Altman DG, Mann H, Berlin J, Dickersin K, Hróbjartsson A, Schulz KF, Parulekar WR, Krleža-Jerić K, Laupacis A, Moher D. SPIRIT 2013 Explanation and Elaboration: Guidance for protocols of clinical trials. BMJ. 2013;346:e7586

		Reporting Item	Page Number
**Administrative information**			
Title	#1	Descriptive title identifying the study design, population, interventions, and, if applicable, trial acronym	1

For peer review only

Trial registration	#2a	Trial identifier and registry name. If not yet registered, name of intended registry	2
Trial registration: data set	#2b	All items from the World Health Organization Trial Registration Data Set	N/A
Protocol version	#3	Date and version identifier	2
Funding	#4	Sources and types of financial, material, and other support	17
Roles and responsibilities: contributorship	#5a	Names, affiliations, and roles of protocol contributors	1, 17
Roles and responsibilities: sponsor contact information	#5b	Name and contact information for the trial sponsor	17
Roles and responsibilities: sponsor and funder	#5c	Role of study sponsor and funders, if any, in study design; collection, management, analysis, and interpretation of data; writing of the report; and the decision to submit the report for publication, including whether they will have ultimate authority over any of these activities	17
Roles and responsibilities: committees	#5d	Composition, roles, and responsibilities of the coordinating centre, steering committee, endpoint adjudication committee, data management team, and	8, 11, 17

For peer review only

		other individuals or groups overseeing the trial, if applicable (see Item 21a for data monitoring committee)	
**Introduction**			
Background and rationale	#6a	Description of research question and justification for undertaking the trial, including summary of relevant studies (published and unpublished) examining benefits and harms for each intervention	3
Background and rationale: choice of comparators	#6b	Explanation for choice of comparators	3
Objectives	#7	Specific objectives or hypotheses	5
Trial design	#8	Description of trial design including type of trial (eg, parallel group, crossover, factorial, single group), allocation ratio, and framework (eg, superiority, equivalence, non-inferiority, exploratory)	5
**Methods: Participants, interventions, and outcomes**			
Study setting	#9	Description of study settings (eg, community clinic, academic hospital) and list of countries where data will be collected. Reference to where list of study sites can be obtained	5

For peer review only

Eligibility criteria	#10	Inclusion and exclusion criteria for participants. If applicable, eligibility criteria for study centres and individuals who will perform the interventions (eg, surgeons, psychotherapists)	5
Interventions: description	#11a	Interventions for each group with sufficient detail to allow replication, including how and when they will be administered	5-6
Interventions: modifications	#11b	Criteria for discontinuing or modifying allocated interventions for a given trial participant (eg, drug dose change in response to harms, participant request, or improving / worsening disease)	6
Interventions: adherance	#11c	Strategies to improve adherence to intervention protocols, and any procedures for monitoring adherence (eg, drug tablet return; laboratory tests)	14
Interventions: concomitant care	#11d	Relevant concomitant care and interventions that are permitted or prohibited during the trial	7, 9-10
Outcomes	#12	Primary, secondary, and other outcomes, including the specific measurement variable (eg, systolic blood pressure), analysis metric (eg, change from baseline, final value, time to event), method of aggregation (eg, median, proportion), and time point for each outcome. Explanation of the clinical relevance of chosen efficacy and harm outcomes is strongly recommended	6-10

For peer review only

Participant timeline	#13	Time schedule of enrolment, interventions (including any run-ins and washouts), assessments, and visits for participants. A schematic diagram is highly recommended (see Figure)	6-7
Sample size	#14	Estimated number of participants needed to achieve study objectives and how it was determined, including clinical and statistical assumptions supporting any sample size calculations	5
Recruitment	#15	Strategies for achieving adequate participant enrolment to reach target sample size	5
**Methods: Assignment of interventions (for controlled trials)**			
Allocation: sequence generation	#16a	Method of generating the allocation sequence (eg, computer-generated random numbers), and list of any factors for stratification. To reduce predictability of a random sequence, details of any planned restriction (eg, blocking) should be provided in a separate document that is unavailable to those who enrol participants or assign interventions	5
Allocation concealment mechanism	#16b	Mechanism of implementing the allocation sequence (eg, central telephone; sequentially numbered, opaque,	6

For peer review only

		sealed envelopes), describing any steps to conceal the sequence until interventions are assigned	
Allocation: implementation	#16c	Who will generate the allocation sequence, who will enrol participants, and who will assign participants to interventions	6
Blinding (masking)	#17a	Who will be blinded after assignment to interventions (eg, trial participants, care providers, outcome assessors, data analysts), and how	6
Blinding (masking): emergency unblinding	#17b	If blinded, circumstances under which unblinding is permissible, and procedure for revealing a participant's allocated intervention during the trial	6
**Methods: Data collection, management, and analysis**			
Data collection plan	#18a	Plans for assessment and collection of outcome, baseline, and other trial data, including any related processes to promote data quality (eg, duplicate measurements, training of assessors) and a description of study instruments (eg, questionnaires, laboratory tests) along with their reliability and validity, if known. Reference to where data collection forms can be found, if not in the protocol	7-9

For peer review only

Data collection plan: retention	#18b	Plans to promote participant retention and complete follow-up, including list of any outcome data to be collected for participants who discontinue or deviate from intervention protocols	7-9
Data management	#19	Plans for data entry, coding, security, and storage, including any related processes to promote data quality (eg, double data entry; range checks for data values). Reference to where details of data management procedures can be found, if not in the protocol	7-10
Statistics: outcomes	#20a	Statistical methods for analysing primary and secondary outcomes. Reference to where other details of the statistical analysis plan can be found, if not in the protocol	9-10
Statistics: additional analyses	#20b	Methods for any additional analyses (eg, subgroup and adjusted analyses)	9-10
Statistics: analysis population and missing data	#20c	Definition of analysis population relating to protocol non-adherence (eg, as randomised analysis), and any statistical methods to handle missing data (eg, multiple imputation)	9-10

Methods: Monitoring

Data monitoring: formal committee	#21a	Composition of data monitoring committee (DMC); summary of its role and reporting structure; statement of whether it is independent from the sponsor and competing interests; and reference to where further	8, 11

For peer review only

		details about its charter can be found, if not in the protocol. Alternatively, an explanation of why a DMC is not needed	
Data monitoring: interim analysis	#21b	Description of any interim analyses and stopping guidelines, including who will have access to these interim results and make the final decision to terminate the trial	9-10
Harms	#22	Plans for collecting, assessing, reporting, and managing solicited and spontaneously reported adverse events and other unintended effects of trial interventions or trial conduct	7-8, 10
Auditing	#23	Frequency and procedures for auditing trial conduct, if any, and whether the process will be independent from investigators and the sponsor	17
**Ethics and dissemination**			
Research ethics approval	#24	Plans for seeking research ethics committee / institutional review board (REC / IRB) approval	11
Protocol amendments	#25	Plans for communicating important protocol modifications (eg, changes to eligibility criteria, outcomes, analyses) to relevant parties (eg, investigators, REC / IRBs, trial participants, trial registries, journals, regulators)	17

For peer review only

Consent or assent	#26a	Who will obtain informed consent or assent from potential trial participants or authorised surrogates, and how (see Item 32)	5
Consent or assent: ancillary studies	#26b	Additional consent provisions for collection and use of participant data and biological specimens in ancillary studies, if applicable	5
Confidentiality	#27	How personal information about potential and enrolled participants will be collected, shared, and maintained in order to protect confidentiality before, during, and after the trial	11
Declaration of interests	#28	Financial and other competing interests for principal investigators for the overall trial and each study site	17
Data access	#29	Statement of who will have access to the final trial dataset, and disclosure of contractual agreements that limit such access for investigators	17
Ancillary and post trial care	#30	Provisions, if any, for ancillary and post-trial care, and for compensation to those who suffer harm from trial participation	N/A
Dissemination policy: trial results	#31a	Plans for investigators and sponsor to communicate trial results to participants, healthcare professionals, the public, and other relevant groups (eg, via publication, reporting in results databases, or other data sharing arrangements), including any publication restrictions	11

For peer review only

Dissemination policy: authorship	#31b	Authorship eligibility guidelines and any intended use of professional writers	11, 17
Dissemination policy: reproducible research	#31c	Plans, if any, for granting public access to the full protocol, participant-level dataset, and statistical code	17

Appendices

Informed consent materials	#32	Model consent form and other related documentation given to participants and authorised surrogates	N/A
Biological specimens	#33	Plans for collection, laboratory evaluation, and storage of biological specimens for genetic or molecular analysis in the current trial and for future use in ancillary studies, if applicable	7-9

The SPIRIT Explanation and Elaboration paper is distributed under the terms of the Creative Commons Attribution License CC-BY-NC. This checklist was completed on 28. January 2023 using https://www.goodreports.org/, a tool made by the EQUATOR Network in collaboration with Penelope.ai

For peer review only

BMJ Open

Protocol for a cluster randomised placebo-controlled trial of adjunctive ivermectin mass drug administration for malaria control on the Bijagós Archipelago of Guinea-Bissau: The MATAMAL trial

Journal:	*BMJ Open*
Manuscript ID	bmjopen-2023-072347.R1
Article Type:	Protocol
Date Submitted by the Author:	05-Jun-2023
Complete List of Authors:	Hutchins, Harry; London School of Hygiene & Tropical Medicine, Clinical Research Department<br>Bradley, John; London School of Hygiene and Tropical Medicine<br>Pretorius, Elizabeth; London School of Hygiene & Tropical Medicine, Department of Disease Control<br>Teixeira da Silva, Eunice; Projecto de Saúde Bandim; Ministério de Saúde Pública<br>Vasileva, Hristina; London School of Hygiene & Tropical Medicine, Department of Infection Biology<br>Jones, Robert; London School of Hygiene & Tropical Medicine, Department of Disease Control<br>Ndiath, Mamadou; Medical Research Council The Gambia<br>dit Massire Soumare, Harouna; Medical Research Council The Gambia<br>Mabey, David; London School of Hygiene & Tropical Medicine, Clinical Research Department<br>Nante, Ernesto; Ministério de Saúde, Programa Nacional de Luta Contra o Paludismo<br>Martins, Cesario; Projecto de Saúde Bandim<br>Logan, James; London School of Hygiene & Tropical Medicine, Department of Disease Control<br>Slater, Hannah; PATH<br>Drakeley, Chris; London School of Hygiene & Tropical Medicine, Department of Infection Biology<br>D'Alessandro, Umberto; Medical Research Council The Gambia<br>Rodrigues, Amabelia; Projecto de Saúde Bandim; Ministério de Saúde Pública<br>Last, Anna R.; London School of Hygiene & Tropical Medicine, Clinical Research Department
**Primary Subject Heading**:	Global health
Secondary Subject Heading:	Epidemiology, Infectious diseases, Public health
Keywords:	Clinical trials < THERAPEUTICS, Malaria, Ivermectin, EPIDEMIOLOGIC STUDIES, Guinea-Bissau, Mass Drug Administration

For peer review only - http://bmjopen.bmj.com/site/about/guidelines.xhtml

Protocol for a cluster randomised placebo-controlled trial of adjunctive ivermectin mass drug administration for malaria control on the Bijagós Archipelago of Guinea-Bissau: The MATAMAL trial

Harry Hutchins*[1], John Bradley[1], Elizabeth Pretorius[2], Eunice Teixeira da Silva[3,4], Hristina Vasileva[1,5], Robert T Jones[2], Mamadou Ousmane Ndiath[6], Harouna dit Massire Soumare[6], David Mabey[1], Jose Ernesto Nante[7], Cesario Martins[3], James G Logan[2,8], Hannah Slater[9], Chris Drakeley[5], Umberto D'Alessandro[6], Amabelia Rodrigues[3,4], Anna Last[1].

1. Clinical Research Department, London School of Hygiene & Tropical Medicine (LSHTM), London, UK
2. Department of Disease Control, LSHTM, London, UK
3. Projecto de Saúde Bandim, Bissau, Guinea-Bissau
4. Ministério de Saúde Pública, Bissau, Guinea-Bissau
5. Department of Infection Biology, LSHTM, London, UK
6. Medical Research Council Unit The Gambia, Fajara, The Gambia
7. Programa Nacional de Luta Contra o Paludismo, Ministério de Saúde, Bissau, Guinea-Bissau
8. Arctech Innovation, London, UK
9. PATH, Seattle, USA

*Corresponding author

Email: harry.hutchins@lshtm.ac.uk

Address: Office 359, LSHTM, London, WC1E7HT, UK

Word count: 4469

For peer review only

KEY WORDS

- Clinical Trials
- Mass Drug Administration
- Guinea-Bissau
- Malaria
- Ivermectin
- Epidemiological Studies

ABSTRACT

Introduction

As malaria declines, innovative tools are required to further reduce transmission and achieve elimination. Mass drug administration (MDA) of artemisinin-based combination therapy (ACT) is capable of reducing malaria transmission where coverage of control interventions is already high, though the impact is short-lived. Combining ACT with ivermectin, an oral endectocide shown to reduce vector survival, may increase its impact whilst also treating ivermectin-sensitive co-endemic diseases and minimizing the potential impact of ACT resistance in this context.

Methods and Analysis

MATAMAL is a cluster-randomised placebo-controlled trial. The trial is being conducted in 24 clusters on the Bijagos Archipelago, Guinea-Bissau where the peak prevalence of *Plasmodium falciparum* (*Pf*) parasitaemia is approximately 15%. Clusters have been randomly allocated to receive MDA with dihydroartemisinin-piperaquine and either ivermectin or placebo. The primary objective is to determine whether the addition of ivermectin MDA is more effective than dihydroartemisinin-piperaquine MDA alone in reducing the prevalence of *Pf* parasitaemia, measured during peak transmission season after two years of seasonal MDA. Secondary objectives include assessing prevalence after one year of MDA; malaria incidence monitored through active and passive surveillance; age-adjusted prevalence of serological markers indicating exposure to *Pf* and anopheline mosquitoes; vector parous rates, species composition, population density and sporozoite rates; prevalence of vector pyrethroid resistance; prevalence of artemisinin resistance in *Pf* using genomic markers; ivermectin's impact on co-endemic diseases; coverage estimates, and the safety of combined MDA.

Ethics and Dissemination

The trial has been approved by the London School of Hygiene & Tropical Medicine Ethics Committee (UK) (19156) and the Comite Nacional de Eticas de Saude (Guinea-Bissau) (084/CNES/INASA/2020). Results will be disseminated in peer-reviewed publications and in discussion with the Bissau-Guinean Ministry of Public Health and participating communities.

Trial Registration

ClinicalTrials.gov (NCT04844905). Includes all items from the World Health Organisation Trial Registration Dataset. This version issued 10/05/2023.

STRENGTH AND LIMITATIONS OF THIS STUDY

- The use of a placebo control arm allows identification of ivermectin's effect on malaria and neglected tropical disease transmission, above that of dihydroartemisinin-piperaquine.

For peer review only - http://bmjopen.bmj.com/site/about/guidelines.xhtml

- Blinding of participants, distributors, investigators and outcome-assessors greatly reduces the risk of bias.
- The geographical separation of island clusters minimizes effects of contamination and spillover between clusters.
- Sample size of 200 over 24 clusters achieves power of 80% for the primary outcome measure.
- The unique setting means results may not be directly generalisable elsewhere, although malaria endemicity and transmission dynamics appear similar to those elsewhere in the region.

INTRODUCTION

Malaria in sub-Saharan Africa has declined dramatically since 2000, with much of the decrease due to vector control methods such as insecticide-treated nets (ITN) and Indoor Residual Spraying (IRS).[1] However, these methods are threatened by increasing insecticide resistance in vectors[2] and their limited efficacy against outdoor-biting or outdoor-resting mosquitoes.[3] There is a clear need for additional vector-control methodologies, including novel tools or novel uses of existing tools.

Mass Drug Administration (MDA) with Artemisinin-based Combination Therapy (ACT) could reduce transmission where coverage of vector-control interventions is high by impacting the human parasite reservoir,[4] reaching even asymptomatic cases which help perpetuate transmission in such settings.[5] Although MDA temporarily reduces malaria prevalence there is little evidence of prolonged effect[6] and modelling predicts that without additional vector-control measures, efficacy is short-lived.[7]

Dihydroartemisinin-piperaquine (DP) is an efficacious and safe antimalarial.[8–10] Piperaquine's long half-life makes it attractive for ACT MDA.[11–14] A cluster-randomised controlled trial in Zambia showed reduced community-level parasite prevalence after two rounds of DP MDA to households with at least one case, particularly in low-transmission areas.[15] DP MDA in The Gambia also resulted in a significant reduction in infection incidence, however these gains were short-lived in higher transmission areas.[16] There is currently little evidence of DP-resistance in Africa,[17] although surveillance data following MDA are lacking.[18]

Ivermectin (IVM) has been widely used in MDA campaigns against onchocerciasis and lymphatic filariasis (LF) in Africa for decades.[19–23] It is an effective endectocide and could be used in MDA to complement vector control strategies, particularly where existing measures have been maximized.[19,24] In West Africa, IVM MDA using 150µg/Kg decreased *An. gambiae* survival and sporozoite rates (SR).[25] Modelling predicts that three consecutive days at this dosage would reduce infectious vector populations by 68% for 60 days.[26] Three daily doses of 300 or 600µg/Kg/day were mosquitocidal at 28 days post-treatment[27,28] and whilst there was a slight increase in minor adverse events (AE) at the higher dose, significantly higher doses have been safely used to treat headlice and onchocerciasis.[20–23,29]

Several clinical trials have demonstrated that combined IVM/ACT MDA is safe, and that it remains an effective anti-malarial treatment and endectocide.[27,28,30–32] IVM/ACT MDA has been shown to be more lethal to vectors than IVM MDA alone[33] and population-level transmission modelling predicts adjunctive IVM would boost the efficacy of DP MDA in reducing malaria prevalence in both high and low prevalence settings.[26,34] Clinical trial data are needed to confirm these findings.

For peer review only - http://bmjopen.bmj.com/site/about/guidelines.xhtml

For peer review only

The RIMDAMAL trial in Burkina Faso reported that communities receiving IVM-only MDA (150-200μg/Kg), given five times at three-weekly intervals after a single dose of IVM and albendazole, saw reduced incidence of clinical malaria in young children compared to communities receiving the single dose alone.[31] However, independent statistical analysis has questioned these findings.[35] The MASSIV trial in The Gambia compared two years of IVM (300-400μg/Kg) and DP MDA on three consecutive days in three consecutive months, against no intervention.[32] It found significantly lower malaria prevalence in the intervention arm, however, it is impossible to separate the effect of IVM from DP due to the absence of MDA in the control arm. Further trials are therefore required not only to confirm optimal dosage and regimen for IVM/ACT MDA, but also its impact on malaria transmission.

The Bijagos Archipelago lies 50km off the Atlantic coast of Guinea-Bissau. Eighteen of the 88 tropical islands are inhabited, supporting a population of approximately 25,000 fishermen, hunter-gatherers and subsistence farmers. There is a long dry season alternating with a rainy season (June – October). The mean temperature is 27.3°C with little monthly variation.[36] Qualitative surveys and daily activity mapping have shown that population movement, commonly for farming or ceremonies, is more limited than in continental Guinea-Bissau, likely due to the islands' remoteness and lack of transport.[37]

Malaria transmission is highly seasonal, peaking at the end of the rains. Serial cross-sectional surveys, powered to estimate prevalence with +/-3% precision, 80% power and 95% confidence, were conducted across the archipelago prior to this trial and showed that, in 2018, qPCR prevalence of *Pf* infection was 8.5% in January (95% CI 6.2-10.8, n=578), 12.3% in July (95% CI 9.4-15.3, n=486) and 17.5% in November (95% CI 16.0-19.1, n=2305) (manuscript in preparation). Infection is highest in the 5-14 years age-group. Bed net coverage is estimated at 92% (95% CI 86%-96%) with reportedly high usage (86%), however, intermittent preventative therapy in pregnancy (IPTp) coverage is lower and, in some areas, non-existent.[38] Indoor residual spraying is not used. One pilot round of seasonal malaria chemoprevention (SMC) was conducted on the islands during the 2020 rainy season. Indoor CDC light trapping indicates *Anopheles gambiae sensu stricto* is the predominant mosquito species during peak malaria transmission, and SR, determined by Circumsporozoite Enzyme-linked Immunosorbent Assays (CSP ELISA) suggest it is the primary malaria vector.[39]

IVM-sensitive Neglected Tropical Diseases (NTDs) such as LF, Soil-Transmitted Helminths (STH) and scabies are co-endemic, the qPCR prevalence of any STH infection being 47.3%, for instance.[40] NTD studies and control measures have demonstrated that MDA is feasible, acceptable and effective in this setting.[41]

Malaria remains a significant public health problem on the Bijagos Archipelago despite high coverage with control measures. The discrete geographical nature of the islands and the associated limitation of population and vector movement further serve to highlight the Bijagos as an ideal study site.[42]

MATAMAL will fill an important knowledge gap as no trial has successfully assessed the effect on malaria of IVM in addition to DP MDA.[43] It will provide valuable data on the effects on co-endemic NTDs, with a view to future integrated strategies. It will complement previous studies in evaluating acceptability, feasibility and cost-effectiveness, adding to the evidence-base examining ivermectin's role in malaria control. Data will be used to inform and update existing models of the impact of MDA on malaria transmission.

For peer review only - http://bmjopen.bmj.com/site/about/guidelines.xhtml

METHODS AND ANALYSIS

Study Aim

To determine whether adjunctive IVM MDA co-administered with DP MDA significantly reduces the population-based prevalence of *Pf* parasitaemia during peak malaria transmission season compared to DP MDA alone.

Study Design

Quadruple-blind (participant, intervention provider, investigator, analyst) cluster-randomised placebo-controlled trial.

Twenty-four clusters have been assigned in a 1:1 ratio to one of two trial arms using restricted randomisation[44]:

1. Intervention (DP and IVM MDA)
2. Control (DP and IVM-placebo MDA)

Restriction variables included population, baseline *Pf* prevalence (qPCR and RDT), vector density, SMC coverage and presence of a health centre. Of 100,000 randomisations, the final randomisation was selected from the approximately 10% satisfying the criteria.

This protocol was developed using SPIRIT reporting guidelines.[45,46] A timeline is shown in Figure 1.

Population and Setting

The trial is being conducted on the Bijagos Archipelago, Guinea-Bissau (Figure 2). Government projections using the last formal census (2009) estimate a population of 25589.[47] Individual islands will constitute 15 clusters. Three larger islands will each be subdivided into 3 clusters providing 24 clusters in total (Figure 3). There will be buffer zones of at least 2.2km between settlements in different clusters using a modified fried-egg principle.[44] Only one populated island, Soga, will be excluded as its very high baseline malaria prevalence is an outlier.

All residents of the islands, defined as anyone sleeping on the island for the majority of a given month, will be invited to participate unless they meet any of the following exclusion criteria:

1. Severe illness
2. Age under 6 months (DP)
3. Height under 90cm or weight under 15Kg (IVM/placebo)
4. Pregnancy (any trimester) or breastfeeding (IVM/placebo). Pregnancy (first trimester) (DP)
5. Known hypersensitivity to either medication
6. Concomitant use of drugs affecting cardiac function or the QTc interval (DP)
7. Travel to a country endemic for *Loa loa* (IVM/placebo)

Residents excluded from MDA will still be eligible for participation in surveys.

An extensive sensitisation campaign will take place ahead of the intervention, led by community health workers (*Agentes de Saude Communitaria*: ASCs). Informed written consent will be obtained after providing written/spoken information (according to literacy) in participants' own language. An independent witness will sign consent forms of illiterate participants. Parents/guardians will be asked to consent on behalf of all children under 18 years. Children aged 12-17 will be invited to sign informed assent forms. Forms and participant information sheets for MDA are included as supplementary materials 1-4. We anticipate very low rates of refusal based on experience with previous interventions, including MDA (for

For peer review only - http://bmjopen.bmj.com/site/about/guidelines.xhtml

trachoma), in these communities, and the perceived importance of malaria.[38]

Intervention and Control

The intervention will be entire community MDA using:

1. Dihydroartemisinin-piperaquine (Alfasigma, Italy)
2. Ivermectin (Laboratorio Elea Phoenix, Argentina)
3. IVM placebo (Laboratorio Elea Phoenix, Argentina)

Standard National Malaria Control Programme interventions (triennial distribution of bed nets, IPTp, case detection and treatment with artemether-lumefantrine) will continue in both arms. The intervention arm will receive DP and IVM, the control arm will receive DP and placebo (Figure 4). A full course of MDA involves three sequential daily doses of both medications, given monthly in July, August and September of 2021 (year 1) and 2022 (year 2). Monthly MDA will begin in all clusters simultaneously, with at least 28 days between each round. Distribution will be conducted by ASCs in their own villages, supervised by experienced cluster-level field assistants. Participant age, sex, weight, pregnancy and eligibility will be recorded by household. Example case record forms, in English, are included in supplementary material 5.

DP is a rapidly-acting artemisinin-based schizontocidal drug used to treat uncomplicated malaria. It is safe, well tolerated, efficacious in clearing *Pf* infection and exhibits prophylactic activity for approximately four weeks.[48]

IVM is an avermectin, active against a range of human parasitic infections and infestations. It is active against *Anopheles spp*. at concentrations present in human blood post-ingestion.[49]

All treatment will be directly observed. Doses will be tablet formulations taken orally with water, with no food for three hours before or after. IVM and IVM-placebo will be visually-identical 6mg tablets dosed at 300µg/Kg to the nearest tablet. DP will be available at doses of 20/160mg and 40/320mg per tablet, administered according to bodyweight. DP can be crushed and mixed with water, and will be readministered in case of vomiting within 30 minutes (half-dose if between 30-60 minutes).

Before delivery to the field, an independent pharmacy team will relabel all ivermectin and placebo bottles with identical “ivermectin or placebo” labels bearing pre-printed cluster codes and recoded lot numbers to prevent unblinding. Only the pharmacist and the independent statistician who conducted the randomisation and anonymised the labels will be unblinded. All laboratory samples will bear only alphanumeric codes. Statistical analysis will be conducted by a blinded statistician. Unblinding will only occur after completion of the primary analysis.

Outcome Measures

Primary Outcome

The population-based qPCR prevalence of *Pf* parasitaemia in all age groups during the peak malaria transmission season after two years of intervention.

This was selected as the most appropriate method for quantifying malaria transmission due to its sensitivity, especially in submicroscopic infections, the reproducibility of methods and results for this well-established assay, and comparability to other trials. qPCR prevalence can also be reliably obtained from dried blood spots, a cheap, simple and robust tool with good participant acceptability.

For peer review only - http://bmjopen.bmj.com/site/about/guidelines.xhtml

Secondary Outcomes

1. Population-based prevalence of *Pf* parasitaemia in all ages, detected by qPCR, during the peak transmission season after the first year of MDA.
2. Incidence of clinical malaria confirmed by pLDH/HRP2 Rapid Diagnostic Test (RDT), determined through passive surveillance of all malaria cases presenting to health facilities throughout the trial.
3. Incidence of clinical malaria identified by RDT (CareStart Malaria PAN pLDH) during active surveillance of a cohort of children aged 5-14 years, during the intervention and peak transmission season.
4. Incidence of malaria infection identified by qPCR and serological analysis during the same period in this cohort of children.
5. Age-adjusted prevalence of serological markers indicating recent exposure to *Pf*.
6. Prevalence of serological markers of recent *Anopheles* exposure.
7. Parity, as a measure of *An. gambiae sensu latu* survival, measured in mosquitoes caught using indoor CDC light traps 7-14 days after the final MDA round in years 1 and 2.
8. Mosquito species composition, population density and SR in mosquitoes caught using indoor CDC light traps.
9. Prevalence of resistance to pyrethroids in anopheline mosquitoes using bioassay methodologies.
10. Prevalence of resistance to artemisinin and partner drugs in humans using molecular markers of resistance.
11. Safety of intervention through monitoring of AE.
12. Impact on IVM-susceptible NTDs (scabies, strongyloidiasis, other STHs and LF), headlice and bedbug infestation using clinical and serological parameters.
13. Cluster-level intervention coverage estimates.
14. MDA acceptability, feasibility and access.
15. Cost-effectiveness of adjunctive IVM in this setting.

Assessment of Outcomes

Primary

A cross-sectional survey will be conducted across all clusters beginning four weeks after the completion of the second year of MDA, during peak transmission. 200 participants will be selected by a two-step randomisation (household; individual) from within a "yolk" of villages in each cluster, purposively defined to capture sufficiently populated villages far from other clusters but logistically feasible to reach. Socio-demographic and GPS data will be collected alongside Dried Blood Spots (DBS) for varATS qPCR analysis, a technique capable of detecting 0.03-0.15 parasites/µL blood, 10 times the sensitivity of standard 18S rRNA qPCR.[50] Standard operating procedures are included as supplementary materials 6-8. Participants will also be asked if they were resident in other clusters during MDA.

In November 2019, mean malaria 18S qPCR prevalence across the islands was 14.8% (95% CI: 14.5-14.9%) (Last et al, Mapping NTDs and Malaria on the Bijagos Archipelago of Guinea-Bissau, unpublished). These results informed a conservative estimate of the co-efficient of variation of 0.46. 200 participants/cluster in 12 clusters/arm provides >80% power to detect a difference between arms if the primary outcome prevalence is 10% in the control arm and 5% in the intervention arm.

A mathematical model was generated to inform sample size calculations and cluster numbers. It was parameterised to simulate prevalence, seasonality, vector control and coverage.

For peer review only - http://bmjopen.bmj.com/site/about/guidelines.xhtml

Assumptions included coverage (70% of eligible persons), DP efficacy (75% of recipients clearing *Pf* parasites), baseline peak qPCR prevalence (21.2%), dominant vector species (*Anopheles gambiae*), fixed time lag to onward infectiousness (12.5 days from infection to gametocyte presence) and ideal conditions such as no population movement and no contamination of participants or vectors across trial arms. A validated pharmacokinetic model was used for estimating participant ivermectin levels over time, and mosquito mortality was assumed to increase relative to this when feeding on a given day after receiving ivermectin. This model predicts control arm qPCR prevalence of 9.2% after one year and 6.5% at three years, compared to 3.9% and 0.8% in the intervention arm, an effect size of 87.8% over control (Figure 5).

Secondary

1. A survey, identical to the primary outcome survey, will be conducted after one year of MDA.
2. On the last day of every month, data on RDT-confirmed incident malaria cases will be recorded from each of the 10 health centres on included islands: age, sex, cluster, fever, RDT performed, RDT result, treatment provided.
3. Cohorts of 50 children aged 5-14 will be followed in 18 clusters. Households will be randomly selected from household-head lists of yolk villages. One child will be randomly selected to participate from each household, more if there are fewer than 50 eligible households. A new cohort will be selected in year 2. Participants will be visited 7-14 days after each round of MDA, as well as during the peak transmission surveys for a total of 8 timepoints. Age, sex, village, GPS and temperature will be recorded. Febrile children will be offered an RDT and treated if positive. All children will be asked about intercurrent clinical malaria episodes, trial adherence and bed net usage. A DBS will be taken for molecular and serological analysis.
4. Assessed as for Outcomes 3 and 5.
5. Serological analysis will be performed on DBS taken during the cross-sectional and cohort surveys described above using a multiplex bead assay on the Luminex MAGPIX platform. The included antigens have antibodies which are associated with recent or long-term exposure to, and/or protection from, *Pf*: MSP1.19, AMA1, GLURP.R2, EBA175.RIII.V, EBA181.RIII.V, Etramp5.Ag1, Etramp4.Ag2, HSP40.Ag1, CSP, SBP1, Hyp2, GEXP18, Rh2.2030, Rh4.1 and Rh5.2.[51–53]
6. Assessed as for Outcome 5.
7. One yolk village will be randomly selected per cluster. Households will be randomly selected in these villages using household-head lists (10 households/cluster in year 1; 15/cluster in year 2). A CDC light trap will be placed inside each household overnight for three consecutive nights, 7 days after each round of MDA in year 1 (18 clusters) and 7 days after the third round of MDA in year 2 (24 clusters). There will be three further nights of trapping during the peak transmission survey. *Anopheles gambiae s.l.* will be identified morphologically.[54] Parity, estimating mosquito survival, will be done using the Detinova ovarian tracheation method.[55]
8. These mosquitoes will also be used to estimate cluster- and trial arm-level species composition (proportion of species present within *An. gambiae* complex), population density (number of mosquitoes/trap/night; a proxy for human biting rate) and sporozoite detection using CSP ELISA.[56] The Entomological Inoculation Rate (EIR), a proxy for human exposure to infectious mosquitoes and a key indicator of local transmission, will be estimated using these data.[57]

For peer review only

For peer review only - http://bmjopen.bmj.com/site/about/guidelines.xhtml

9. Anopheline mosquitoes will be reared from locally-caught larvae during the year 2 rainy season. Batches of 100 adult females will be tested for resistance to alpha-cypermethrin, permethrin and deltamethrin, with and without the synergist piperonyl butoxide, using CDC bottle bioassays and WHO test tubes at once, twice and five-times the discriminating dose.[58,59] Two hundred mosquitoes, including all resistant individuals, will undergo PCR species identification.[60]
10. DBS samples positive for *Pf* DNA will undergo further extraction, amplification by PCR[61] and sequencing using Illumina-based technology to identify genetic mutations previously associated with antimalarial drug resistance, including polymorphisms in *pfkelch, pfmdr1, pfcrt* and *pfexo* genes, before being analysed using bioinformatic tools.[62,63] Selective whole genome amplification steps will be conducted on the initial DNA extract to increase the quantity of *Pf* DNA. A cohort of RDT-positive malaria patients will also be recruited from select health centres during the rainy season to provide serial DBS throughout treatment. These will then undergo qPCR analysis for evidence of treatment success/failure and identification of resistance markers as described above.
11. AE data will be collected actively by distributors during MDA and for 48 hours after, and passively by sensitising participants and health centre nurses to report AEs. ASCs will monitor births, deaths and miscarriages. Supervisors will send daily reports to the trial manager, who will generate annual reports for the Data Safety Monitoring Board (DSMB). Cross-sectional and cohort surveys include *post hoc* questions on AEs. All doses will be directly observed. *Loa loa* is not endemic to this region, greatly reducing the risk of IVM-associated encephalitis. Appropriate health-seeking advice will provided by distributors to all participants reporting AEs.
12. A population-based cross-sectional survey will be performed in all clusters during the 2023 dry season i.e. after all MDA has been delivered: 100 households will be randomly selected from household-head lists in each cluster yolk, and one child aged 2-10 invited to participate from each household. Multiple children will be invited if the sample size is not met. Skin will be systematically examined to identify scabies, and impetigo in positive cases. Headlice examination and fine-tooth combing above a white cloth will occur over 10 minutes. Bedframes, mattresses, nets and adjacent walls will be inspected with torchlight for signs of bedbugs, or eggs, over ten minutes. A stool sample (5g) will be collected and stored in ethanol to undergo multiplex parasite PCR (STH, Strongyloides).[64–66] DBS will be collected for serological testing for LF, strongyloidiasis and STH.[67–69]
13. MDA distributors enter administration data into forms listing every householder, including absent members, and capture data on daily refusals, absences and exclusions. These forms generate a *de facto* census and can be used calculate monthly cluster-level coverage, absence, refusal and exclusion statistics.
14. Qualitative and quantitative analysis of MDA acceptability, feasibility and access, including population movement, will be outlined in a separate protocol.
15. Cost-effectiveness analysis will be detailed in a separate protocol.

Data Analysis Plan

Data will be entered by trained MDA delivery teams under the supervision of highly-trained and experienced local supervisors. Senior trial management will regularly review data entry and offer retraining as needed. Sample ID numbers will be electronically captured or double-

For peer review only - http://bmjopen.bmj.com/site/about/guidelines.xhtml

entered. A communication network will be established with remote villages to facilitate monitoring and correction of errors found on review.

All analysis will be intention-to-treat. Analysis will be performed using STATA software (StataCorp) and R.

Cluster-, arm-level and overall demographic characteristics will be presented using descriptive statistics, including age, sex, bed net use, household size, and village/cluster populations.

Primary Outcome

Trial arms will be compared using a t-test on cluster-level malaria qPCR prevalences. A risk difference, 95% CI and P-value will be presented. Analyses adjusting for age groups (<5, 5-14, 15 and above), bed net use and presence of a health centre will be presented for this and all secondary outcomes.

Secondary Outcomes

1. As for the primary outcome, using data from the year 1 survey.
2. Cluster-level incidence rates will be compared using a t-test. If the distribution is markedly skewed then the natural logarithm of each cluster-level summary will be taken. If some clusters have zero events, then 0.5 events will be added to each cluster. Rate differences, 95% CI and P-value will be presented.
3. As for Outcome 2, using RDT-confirmed incidence rates from the two annual cohorts of children.
4. As for Outcome 3, using qPCR-confirmed incidence rates. Serological analysis described in Outcome 5.
5. Cluster-level serological responses to malaria antigens will be presented as both continuous Median Fluorescent Intensity (MFI) data, a proxy for antibody titres, and binary seropositivity prevalence, with a seropositivity threshold of 3sd above mean malaria-naïve MFI responses. MFI will be compared between arms using mixed effect linear regression, with a random effect for cluster. Seropositivity will be compared in the same way as the primary outcome, including analysis by age-group. Cluster-level antigen-specific Seroconversion Rates (SCR) will be generated using serocatalytic models relying on seroprevalence, and age-seroprevalence plots will be generated by fitting data to a reversible catalytic conversion model using maximum likelihood methods. Trial arms' SCR will be compared using a t-test.[53]
6. As for Outcome 5, using the gSG6 antigen and not employing SCR.
7. Cluster-level anopheline parity will be compared between arms using a t-test. Adjustment will be made for species, MDA round, time relative to MDA date, temperature, humidity and rainfall. Parity difference, 95% CI and P-value will be presented.
8. The proportion of each species identified, and mosquito densities, will be presented by cluster and arm, and compared using chi-square and t-tests. Adjustment will be made for study month and year. Differences, 95% CIs and P-values will be presented. If the distribution of densities is markedly skewed, a log transformation will be applied. SR will be compared between arms using a t-test on cluster-level SR with adjustments and covariates as described for parity analysis above. Risk difference, 95% CI and P-value will be presented. EIR will be compared with a t-test on cluster level summaries.

For peer review only - http://bmjopen.bmj.com/site/about/guidelines.xhtml

9. Species composition and level of resistance will be presented using descriptive statistics. Dose-response curves will be generated for each insecticide and probit regression analysis will be performed on these data using maximum likelihood or least squares methods.
10. The number of alleles associated with antimalarial resistance will be compared over time, before and after MDA. However, appropriate statistical tests will need to be selected depending on the allele sample size before and after MDA.
11. Descriptive statistics will be presented on the number, nature, severity and relatedness of all reported AE, with additional details for serious AE. Cluster-level and age-group data will be presented. Rates will be compared between arms.
12. As for the primary outcome, and Outcome 5.
13. Cluster-, arm-level and overall coverage will be presented for each month and year of intervention, and for the trial overall. The denominator for these proportions will be the total number of people recorded on MDA Administration Records by distributors in each cluster, whether receiving MDA or not. Refusals, exclusions and absences will be presented similarly.
14. Qualitative and quantitative analysis of MDA acceptability, feasibility and access will be outlined in a separate protocol.
15. Cost-effectiveness analysis will be detailed in a separate protocol.

Patient and Public Involvement

Qualitative studies report that this population almost unanimously consider malaria to be a significant problem in their homes and the region, that additional malaria control measures would be welcome, and that MDA is acceptable,[38,70] all of which informed MATAMAL's design.

All field assistants and the deputy trial manager are local residents and MDA will be delivered by ASCs within their own communities. Methods were finalised in discussion with these stakeholders, the Regional Health Directorate and MINSAP. There will be monthly feedback sessions with ASCs and staff during MDA and surveys. Trial outcomes, social science, public engagement and qualitative work will be presented to stakeholders and communities using plans developed with the contributors themselves.

TRIAL STATUS

All necessary approvals are in place. A baseline malaria prevalence survey was completed in November 2019. Two years of MDA has successfully been delivered across all clusters, alongside two annual cohort surveys and entomological sample collection. Two annual peak transmission surveys and a dry season NTD survey have been completed. Qualitative and social science work has taken place throughout. Laboratory testing is ongoing ahead of statistical analysis.

ETHICS AND DISSEMINATION

MATAMAL will be reported according to CONSORT guidance.[71] It is a collaboration between LSHTM, Medical Research Council The Gambia and MINSAP. Ethical approval has been obtained from LSHTM Research Ethics Committee (UK) (19156) and CNES (Guinea-Bissau) (084/CNES/INASA/2020), with additional regulatory approvals from The Gambia. Any changes to the protocol will be agreed by investigators and submitted to these same bodies for

For peer review only - http://bmjopen.bmj.com/site/about/guidelines.xhtml

approval. The independent DSMB will oversee trial safety through annual meetings, advising the Trial Steering Committee on ongoing trial conduct. Members of these committees are listed in supplemental material 9. Independent audit may be carried out by LSHTM, our funders or MRC The Gambia. This work abides by the Declaration of Helsinki[72] and Good Clinical Practice.[73]

Electronic forms are encrypted upon submission to a LSHTM HERA-compliant secure server. Paper forms will be kept in limited-access locked storage at the site office. Data will be de-identified, except for consent and MDA record forms. All data will be held for a minimum of 7 years, including in LSHTM electronic repositories, whence access to de-identified data can be requested. All investigators will have access to cleaned databases.

Results will be published in peer-review journals, presented at conferences and to local collaborators. Field staff, nurses and ASCs will disseminate findings to participating communities in appropriate and accessible formats. Authorship will be decided on paper-by-paper basis, to recognise significant contributions to design, conduct, analysis and reporting. Writing will not be out-sourced.

AUTHORS’ CONTRIBUTIONS

AL, the principal investigator, conceived the initial design of the study, secured funding and contributed significantly to writing this protocol. HH, the trial manager, runs all field operations, consulted on field methods and generated this manuscript. EP, RTJ and JGL drafted the entomology methods. JB provided statistical expertise. HS modelled the trial’s anticipated impact. CD and HV provided serological expertise. MON and HdMS provided laboratory supervision and support. ETS, CM, AR and JEN, our local collaborators, provided extensive information on local context and logistics. UD and DM supervised the writing of this protocol and provided expertise on trial conduct.

London School of Hygiene & Tropical Medicine (Keppel Street, London, WC1E 7HT, UK. +442076368636) is the trial sponsor. The sponsor and funders had no role in trial design, management or analysis.

FUNDING STATEMENT

This work was supported by a Joint Global Health Trials award (NIHR, MRC, Wellcome, FCDO), funder reference MR/S005013/1.

COMPETING INTERESTS

JGL declares he is founder/CEO of Arctech Innovation, a company which aims to design mosquito lures and malaria diagnostics.

ACKNOWLEDGEMENTS

We wish to express our sincere thanks to the health workers and residents of the Bijagos Archipelago, whose patience and support is the foundation of this trial.

For peer review only

For peer review only - http://bmjopen.bmj.com/site/about/guidelines.xhtml

REFERENCES

1. Bhatt S, Weiss DJ, Cameron E, Bisanzio D, Mappin B, Dalrymple U, et al. The effect of malaria control on Plasmodium falciparum in Africa between 2000 and 2015. Nature [Internet]. 2015 Oct 8 [cited 2019 Sep 23];526(7572):207–11. Available from: http://www.ncbi.nlm.nih.gov/pubmed/26375008
2. Barnes KG, Weedall GD, Ndula M, Irving H, Mzihalowa T, Hemingway J, et al. Genomic Footprints of Selective Sweeps from Metabolic Resistance to Pyrethroids in African Malaria Vectors Are Driven by Scale up of Insecticide-Based Vector Control. PLOS Genet [Internet]. 2017 Feb 1 [cited 2022 Oct 4];13(2):e1006539. Available from: https://journals.plos.org/plosgenetics/article?id=10.1371/journal.pgen.1006539
3. Griffin JT, Hollingsworth TD, Okell LC, Churcher TS, White M, Hinsley W, et al. Reducing Plasmodium falciparum malaria transmission in Africa: a model-based evaluation of intervention strategies. PLoS Med [Internet]. 2010 Aug [cited 2022 Oct 4];7(8). Available from: https://pubmed.ncbi.nlm.nih.gov/20711482/
4. Global Malaria Programme. Malaria Policy Advisory Committee meeting report [Internet]. Geneva; 2016 Mar [cited 2022 Oct 4]. Available from: https://www.who.int/publications/i/item/WHO-HTM-GMP-MPAC-2016.3
5. Bousema T, Okell L, Felger I, Drakeley C. Asymptomatic malaria infections: detectability, transmissibility and public health relevance. Nat Rev Microbiol [Internet]. 2014 Dec 18 [cited 2022 Jan 11];12(12):833–40. Available from: https://pubmed.ncbi.nlm.nih.gov/25329408/
6. Shah MP, Hwang J, Choi L, Lindblade KA, Kachur SP, Desai M. Mass drug administration for malaria. Cochrane Database Syst Rev [Internet]. 2021 Sep 29 [cited 2022 Oct 4];2021(9). Available from: /pmc/articles/PMC8479726/
7. Brady OJ, Slater HC, Pemberton-Ross P, Wenger E, Maude RJ, Ghani AC, et al. Role of mass drug administration in elimination of Plasmodium falciparum malaria: a consensus modelling study. Lancet Glob Heal [Internet]. 2017 Jul 1 [cited 2022 Oct 4];5(7):e680–7. Available from: https://pubmed.ncbi.nlm.nih.gov/28566213/
8. Zani B, Gathu M, Donegan S, Olliaro PL, Sinclair D. Dihydroartemisinin-piperaquine for treating uncomplicated Plasmodium falciparum malaria. Cochrane database Syst Rev [Internet]. 2014 Jan 20 [cited 2022 Oct 5];2014(1). Available from: https://pubmed.ncbi.nlm.nih.gov/24443033/
9. Keating GM. Dihydroartemisinin/Piperaquine: a review of its use in the treatment of uncomplicated Plasmodium falciparum malaria. Drugs [Internet]. 2012 [cited 2022 Oct 5];72(7):937–61. Available from: https://pubmed.ncbi.nlm.nih.gov/22515619/
10. Adjei A, Narh-Bana S, Amu A, Kukula V, Nagai RA, Owusu-Agyei S, et al. Treatment outcomes in a safety observational study of dihydroartemisinin/piperaquine (Eurartesim®) in the treatment of uncomplicated malaria at public health facilities in four African countries. Malar J [Internet]. 2016 Jan 27 [cited 2022 Oct 5];15(1):1–10. Available from: https://malariajournal.biomedcentral.com/articles/10.1186/s12936-016-1099-7
11. Pekyi D, Ampromfi AA, Tinto H, Traoré-Coulibaly M, Tahita MC, Valéa I, et al. Four artemisinin-based treatments in African pregnant women with malaria. N Engl J Med [Internet]. 2016 Mar 10 [cited 2022 Oct 5];28(3):139–49. Available from: https://www.nejm.org/doi/full/10.1056/NEJMoa1508606
12. Pfeil J, Borrmann S, Bassat Q, Mulenga M, Talisuna A, Tozan Y. An Economic Evaluation of the Posttreatment Prophylactic Effect of Dihydroartemisinin-Piperaquine Versus Artemether-Lumefantrine for First-Line Treatment of Plasmodium falciparum Malaria Across Different Transmission Settings in Africa. Am J Trop Med Hyg [Internet]. 2015 Nov 4 [cited 2022 Oct 5];93(5):961–6. Available from: https://pubmed.ncbi.nlm.nih.gov/26240155/

For peer review only - http://bmjopen.bmj.com/site/about/guidelines.xhtml

13. Eisele TP, Silumbe K, Finn T, Chalwe V, Kamuliwo M, Hamainza B, et al. Assessing the effectiveness of household-level focal mass drug administration and community-wide mass drug administration for reducing malaria parasite infection prevalence and incidence in Southern Province, Zambia: Study protocol for a community randomized controlled trial. Trials [Internet]. 2015 Aug 13 [cited 2022 Oct 5];16(1):1–13. Available from: https://trialsjournal.biomedcentral.com/articles/10.1186/s13063-015-0862-3
14. Zani B, Gathu M, Donegan S, Olliaro PL, Sinclair D. Dihydroartemisinin-piperaquine for treating uncomplicated Plasmodium falciparum malaria. Cochrane database Syst Rev. 2014 Jan;2014(1).
15. Eisele TP, Bennett A, Silumbe K, Finn TP, Chalwe V, Kamuliwo M, et al. Short-term Impact of Mass Drug Administration With Dihydroartemisinin Plus Piperaquine on Malaria in Southern Province Zambia: A Cluster-Randomized Controlled Trial. J Infect Dis [Internet]. 2016 Dec 12 [cited 2022 Oct 5];214(12):1831. Available from: /pmc/articles/PMC5142084/
16. Mwesigwa J, Achan J, Affara M, Wathuo M, Worwui A, Mohammed NI, et al. Mass Drug Administration With Dihydroartemisinin-piperaquine and Malaria Transmission Dynamics in The Gambia: A Prospective Cohort Study. Clin Infect Dis [Internet]. 2019 Jul 2 [cited 2022 Oct 4];69(2):278–86. Available from: https://pubmed.ncbi.nlm.nih.gov/30304511/
17. World Health Organization. Strategy to respond to antimalarial drug resistance in Africa [Internet]. Geneva; 2022. Available from: https://www.who.int/publications/i/item/9789240060265
18. Moss S, Mańko E, Krishna S, Campino S, Clark TG, Last A. How has mass drug administration with dihydroartemisinin-piperaquine impacted molecular markers of drug resistance? A systematic review. Malar J [Internet]. 2022 Dec 1 [cited 2023 May 10];21(1):1–23. Available from: https://malariajournal.biomedcentral.com/articles/10.1186/s12936-022-04181-y
19. Chaccour C, Hammann F, Rabinovich NR. Ivermectin to reduce malaria transmission I. Pharmacokinetic and pharmacodynamic considerations regarding efficacy and safety. Malar J [Internet]. 2017 [cited 2019 Sep 6];16. Available from: https://www.ncbi.nlm.nih.gov/pmc/articles/PMC5402169/
20. Awadzi K, Opoku NO, Addy ET, Quartey BT. The chemotherapy of onchocerciasis. XIX: The clinical and laboratory tolerance of high dose ivermectin - PubMed. Trop Med Parasitol [Internet]. 1995 Jun [cited 2022 Oct 4];46(2):131–7. Available from: https://pubmed.ncbi.nlm.nih.gov/8525285/
21. Guzzo CA, Furtek CI, Porras AG, Chen C, Tipping R, Clineschmidt CM, et al. Safety, tolerability, and pharmacokinetics of escalating high doses of ivermectin in healthy adult subjects. J Clin Pharmacol [Internet]. 2002 Oct [cited 2022 Oct 4];42(10):1122–33. Available from: https://pubmed.ncbi.nlm.nih.gov/12362927/
22. Kamgno J, Gardon J, Gardon-Wendel N, Demanga-Ngangue A, Duke BOL, Boussinesq M. Adverse systemic reactions to treatment of onchocerciasis with ivermectin at normal and high doses given annually or three-monthly. Trans R Soc Trop Med Hyg [Internet]. 2004 Aug [cited 2022 Oct 4];98(8):496–504. Available from: https://pubmed.ncbi.nlm.nih.gov/15186939/
23. Fobi G, Gardon J, Kamgno J, Aimard-Favennec L, Lafleur C, Gardon-Wendel N, et al. A randomized, double-blind, controlled trial of the effects of ivermectin at normal and high doses, given annually or three-monthly, against Onchocerca volvulus: ophthalmological results. Trans R Soc Trop Med Hyg [Internet]. 2005 Apr [cited 2022 Oct 4];99(4):279–89. Available from: https://pubmed.ncbi.nlm.nih.gov/15708387/
24. WHO. Malaria Policy Advisory Committee to the WHO: Conclusions and recommendations of eighth biannual meeting (September 2015). Malar J [Internet].

For peer review only - http://bmjopen.bmj.com/site/about/guidelines.xhtml

2016 Feb 24 [cited 2022 Oct 5];15(1):1–12. Available from: https://malariajournal.biomedcentral.com/articles/10.1186/s12936-016-1169-x
25. Alout H, Krajacich BJ, Meyers JI, Grubaugh ND, Brackney DE, Kobylinski KC, et al. Evaluation of ivermectin mass drug administration for malaria transmission control across different West African environments. Malar J. 2014 Nov 3;13(1).
26. Slater HC, Walker PGT, Bousema T, Okell LC, Ghani AC. The Potential Impact of Adding Ivermectin to a Mass Treatment Intervention to Reduce Malaria Transmission: A Modelling Study. J Infect Dis [Internet]. 2014 Dec 15 [cited 2019 Oct 3];210(12):1972–80. Available from: http://www.ncbi.nlm.nih.gov/pubmed/24951826
27. Smit MR, Ochomo EO, Aljayyoussi G, Kwambai TK, Abong'o BO, Chen T, et al. Safety and mosquitocidal efficacy of high-dose ivermectin when co-administered with dihydroartemisinin-piperaquine in Kenyan adults with uncomplicated malaria (IVERMAL): a randomised, double-blind, placebo-controlled trial. Lancet Infect Dis [Internet]. 2018 Jun 1 [cited 2019 Sep 2];18(6):615–26. Available from: https://www.sciencedirect.com/science/article/pii/S1473309918301634
28. Smit MR, Ochomo E, Aljayyoussi G, Kwambai T, Abong'o B, Bayoh N, et al. Efficacy and Safety of High-Dose Ivermectin for Reducing Malaria Transmission (IVERMAL): Protocol for a Double-Blind, Randomized, Placebo-Controlled, Dose-Finding Trial in Western Kenya. JMIR Res Protoc [Internet]. 2016 Nov 17 [cited 2022 Oct 4];5(4):e213. Available from: https://pubmed.ncbi.nlm.nih.gov/27856406/
29. Abramowicz M, Zuccotti G. Drugs for Head Lice. JAMA [Internet]. 2017 May 16 [cited 2022 Oct 4];317(19):2010–1. Available from: https://pubmed.ncbi.nlm.nih.gov/28510677/
30. Ouedraogo AL, Bastiaens GJH, Tiono AB, Guelbeogo WM, Kobylinski KC, Ouedraogo A, et al. Efficacy and Safety of the Mosquitocidal Drug Ivermectin to Prevent Malaria Transmission After Treatment: A Double-Blind, Randomized, Clinical Trial. Clin Infect Dis [Internet]. 2015 Feb 1 [cited 2019 Sep 6];60(3):357–65. Available from: https://academic.oup.com/cid/article-lookup/doi/10.1093/cid/ciu797
31. Foy BD, Alout H, Seaman JA, Rao S, Magalhaes T, Wade M, et al. Efficacy and risk of harms of repeat ivermectin mass drug administrations for control of malaria (RIMDAMAL): a cluster-randomised trial. Lancet (London, England) [Internet]. 2019 Apr 13 [cited 2019 Jul 13];393(10180):1517–26. Available from: http://www.ncbi.nlm.nih.gov/pubmed/30878222
32. Dabira ED, Soumare HM, Conteh B, Ceesay F, Ndiath MO, Bradley J, et al. Mass drug administration of ivermectin and dihydroartemisinin–piperaquine against malaria in settings with high coverage of standard control interventions: a cluster-randomised controlled trial in The Gambia. Lancet Infect Dis. 2021 Dec;0(0).
33. Kobylinski KC, Jittamala P, Hanboonkunupakarn B, Pukrittayakamee S, Pantuwatana K, Phasomkusolsil S, et al. Safety, Pharmacokinetics, and Mosquito-Lethal Effects of Ivermectin in Combination With Dihydroartemisinin-Piperaquine and Primaquine in Healthy Adult Thai Subjects. Clin Pharmacol Ther [Internet]. 2020 May 1 [cited 2022 Oct 5];107(5):1221–30. Available from: https://pubmed.ncbi.nlm.nih.gov/31697848/
34. Slater HC, Foy BD, Kobylinski K, Chaccour C, Watson OJ, Hellewell J, et al. Ivermectin as a novel complementary malaria control tool to reduce incidence and prevalence: a modelling study. Lancet Infect Dis. 2020 Apr 1;20(4):498–508.
35. Bradley J, Moulton LH, Hayes R. Analysis of the RIMDAMAL trial. Lancet [Internet]. 2019 Sep 21 [cited 2023 Jan 16];394(10203):1005–6. Available from: http://www.thelancet.com/article/S0140673619316630/fulltext
36. Metroblue. Observed historical climate & weather data for Bubaque - meteoblue [Internet]. Metroblue. 2022 [cited 2022 Feb 4]. Available from:

For peer review only

For peer review only - http://bmjopen.bmj.com/site/about/guidelines.xhtml

https://www.meteoblue.com/en/weather/historyclimate/climateobserved/bubaque_guinea-bissau_2374583

37. Durrans S, Last A, Boiro H, Goncalves A, Mabey D, Greenland K. “Moving like birds”: A qualitative study of population mobility and health implications in the Bijagós Islands, Guinea Bissau. Soc Sci Med [Internet]. 2019 Jun 1 [cited 2019 Oct 1];230:204–13. Available from: https://www.sciencedirect.com/science/article/pii/S0277953619301613
38. Hutchins H, Power G, Ant T, Teixeira da Silva E, Goncalves A, Rodrigues A, et al. A survey of knowledge, attitudes and practices regarding malaria and bed nets on Bubaque Island, Guinea-Bissau. Malar J [Internet]. 2020 Dec 1 [cited 2022 Jan 31];19(1):1–15. Available from: https://malariajournal.biomedcentral.com/articles/10.1186/s12936-020-03469-1
39. Ant T, Foley E, Tytheridge S, Johnston C, Goncalves A, Ceesay S, et al. A survey of Anopheles species composition and insecticide resistance on the island of Bubaque, Bijagos Archipelago, Guinea-Bissau. Malar J [Internet]. 2020 Jan 15 [cited 2022 Feb 1];19(1):1–9. Available from: https://malariajournal.biomedcentral.com/articles/10.1186/s12936-020-3115-1
40. Farrant O, Marlais T, Houghton J, Goncalves A, Teixeira da Silva Cassama E, Cabral MG, et al. Prevalence, risk factors and health consequences of soil-transmitted helminth infection on the Bijagos Islands, Guinea Bissau: A community-wide cross-sectional study. PLoS Negl Trop Dis [Internet]. 2020 Dec 1 [cited 2023 Jan 20];14(12):e0008938. Available from: /pmc/articles/PMC7773412/
41. Last AR, Burr SE, Harding-Esch E, Cassama E, Nabicassa M, Roberts CH, et al. The impact of a single round of community mass treatment with azithromycin on disease severity and ocular Chlamydia trachomatis load in treatment-naïve trachoma-endemic island communities in West Africa. Parasit Vectors [Internet]. 2017 Dec 28 [cited 2022 Oct 4];10(1). Available from: https://pubmed.ncbi.nlm.nih.gov/29282126/
42. Jones RT, Pretorius E, Ant TH, Bradley J, Last A, Logan JG. The use of islands and cluster-randomized trials to investigate vector control interventions: a case study on the Bijagós archipelago, Guinea-Bissau. Philos Trans R Soc B Biol Sci [Internet]. 2021 Feb 2 [cited 2023 Jan 24];376(1818). Available from: /pmc/articles/PMC7776941/
43. de Souza DK, Thomas R, Bradley J, Leyrat C, Boakye DA, Okebe J. Ivermectin treatment in humans for reducing malaria transmission. Cochrane Database Syst Rev [Internet]. 2021 Jun 29 [cited 2023 Jan 23];2021(6). Available from: https://www.cochranelibrary.com/cdsr/doi/10.1002/14651858.CD013117.pub2/full
44. Hayes RJ, Moulton LH. Cluster randomised trials. 2nd ed. Chapman and Hall/CRC; 2017.
45. Standard Protocol Items: Recommendations for Interventional Trials. SPIRIT - Guidance for Clinical Trial Protocols [Internet]. 2013 [cited 2022 Sep 23]. Available from: https://www.spirit-statement.org/
46. Chan A-W, Tetzlaff J, Gotzsche P, Altman D, Mann H, Berlin J, et al. SPIRIT 2013 Explanation and Elaboration: Guidance for protocols of clinical trials. BMJ [Internet]. 2013 [cited 2023 Jan 28];346(e7586). Available from: https://www.goodreports.org/reporting-checklists/spirit/
47. INEP Guine-Bissau. Guinea-Bissau 2009 Census [Internet]. Guinea-Bissau Data Portal. 2009 [cited 2020 Apr 16]. Available from: https://guinebissau.opendataforafrica.org/amthtjd/guinea-bissau-2009-census
48. Hung TY, Davis TME, Ilett KF, Karunajeewa H, Hewitt S, Denis MB, et al. Population pharmacokinetics of piperaquine in adults and children with uncomplicated falciparum or vivax malaria. Br J Clin Pharmacol [Internet]. 2004 Mar [cited 2022 Oct

For peer review only

For peer review only - http://bmjopen.bmj.com/site/about/guidelines.xhtml

13];57(3):253. Available from: /pmc/articles/PMC1884452/

49. Chaccour C, Lines J, Whitty CJM. Effect of Ivermectin on Anopheles gambiae Mosquitoes Fed on Humans: The Potential of Oral Insecticides in Malaria Control . J Infect Dis. 2010 Jul;202(1):113–6.
50. Hofmann N, Mwingira F, Shekalaghe S, Robinson LJ, Mueller I, Felger I. Ultra-Sensitive Detection of Plasmodium falciparum by Amplification of Multi-Copy Subtelomeric Targets. PLOS Med [Internet]. 2015 Mar 1 [cited 2023 Jan 21];12(3):e1001788. Available from: https://journals.plos.org/plosmedicine/article?id=10.1371/journal.pmed.1001788
51. Wu L, Hall T, Ssewanyana I, Oulton T, Patterson C, Vasileva H, et al. Optimisation and standardisation of a multiplex immunoassay of diverse Plasmodium falciparum antigens to assess changes in malaria transmission using sero-epidemiology. Wellcome Open Res. 2020 Apr 23;4:26.
52. Helb DA, Tetteh KKA, Felgner PL, Skinner J, Hubbard A, Arinaitwe E, et al. Novel serologic biomarkers provide accurate estimates of recent Plasmodium falciparum exposure for individuals and communities. Proc Natl Acad Sci U S A [Internet]. 2015 Aug 11 [cited 2022 Jan 7];112(32):E4438–47. Available from: /pmc/articles/PMC4538641/
53. Drakeley CJ, Corran PH, Coleman PG, Tongren JE, McDonald SLR, Carneiro I, et al. Estimating medium- and long-term trends in malaria transmission by using serological markers of malaria exposure. Proc Natl Acad Sci [Internet]. 2005 Apr 5 [cited 2022 Jan 10];102(14):5108–13. Available from: https://www.pnas.org/content/102/14/5108
54. Coetzee M. Key to the females of Afrotropical Anopheles mosquitoes (Diptera: Culicidae). Malar J [Internet]. 2020 Feb 13 [cited 2023 Jan 24];19(1):1–20. Available from: https://malariajournal.biomedcentral.com/articles/10.1186/s12936-020-3144-9
55. Detinova T. Age-grouping methods in diptera of medical importance [Internet]. Geneva: WHO; 1962 [cited 2023 Jan 24]. 1–213 p. Available from: https://apps.who.int/iris/bitstream/handle/10665/41724/WHO_MONO_47_(part1).pdf;sequence=1
56. Wirtz RA, Zavala F, Charoenvit Y, Campbell GH, Burkot TR, Schneider I, et al. Comparative testing of monoclonal antibodies against Plasmodium falciparum sporozoites for ELISA development. Bull World Health Organ [Internet]. 1987 [cited 2023 Jan 20];65(1):39. Available from: /pmc/articles/PMC2490858/?report=abstract
57. Kilama M, Smith DL, Hutchinson R, Kigozi R, Yeka A, Lavoy G, et al. Estimating the annual entomological inoculation rate for Plasmodium falciparum transmitted by Anopheles gambiae s.l. using three sampling methods in three sites in Uganda. Malar J [Internet]. 2014 Mar 21 [cited 2023 Jan 24];13(1). Available from: https://pubmed.ncbi.nlm.nih.gov/24656206/
58. Brogdon W, Chan A. Guideline for Evaluating Insecticide Resistance in Vectors Using the CDC Bottle Bioassay [Internet]. [cited 2023 Jan 20]. p. 1–28. Available from: https://www.cdc.gov/malaria/resources/pdf/fsp/ir_manual/ir_cdc_bioassay_en.pdf
59. WHO. Standard operating proceudre for testing insecticide susceptibility of adult mosquitoes in WHO test tubes [Internet]. Geneva; 2022. Available from: https://www.who.int/publications/i/item/9789240043831
60. Fanello C, Santolamazza F, Della Torre A. Simultaneous identification of species and molecular forms of the Anopheles gambiae complex by PCR-RFLP. Med Vet Entomol [Internet]. 2002 Dec 1 [cited 2023 Jan 20];16(4):461–4. Available from: https://onlinelibrary.wiley.com/doi/full/10.1046/j.1365-2915.2002.00393.x
61. Oyola SO, Ariani C V., Hamilton WL, Kekre M, Amenga-Etego LN, Ghansah A, et al.

For peer review only

For peer review only - http://bmjopen.bmj.com/site/about/guidelines.xhtml

Whole genome sequencing of Plasmodium falciparum from dried blood spots using selective whole genome amplification. Malar J [Internet]. 2016 Dec 20 [cited 2023 Jan 23];15(1):1–12. Available from: https://malariajournal.biomedcentral.com/articles/10.1186/s12936-016-1641-7

62. Nag S, Dalgaard MD, Kofoed PE, Ursing J, Crespo M, Andersen LOB, et al. High throughput resistance profiling of Plasmodium falciparum infections based on custom dual indexing and Illumina next generation sequencing-technology. Sci Rep [Internet]. 2017 Dec 1 [cited 2023 Jan 23];7(1). Available from: /pmc/articles/PMC5445084/

63. Moss S, Mańko E, Vasileva H, Da Silva ET, Goncalves A, Osborne A, et al. Population dynamics and drug resistance mutations in Plasmodium falciparum on the Bijagós Archipelago, Guinea-Bissau. Sci Rep [Internet]. 2023 Dec 1 [cited 2023 May 11];13(1):6311. Available from: /pmc/articles/PMC10113324/

64. Llewellyn S, Inpankaew T, Nery SV, Gray DJ, Verweij JJ, Clements ACA, et al. Application of a Multiplex Quantitative PCR to Assess Prevalence and Intensity Of Intestinal Parasite Infections in a Controlled Clinical Trial. Bethony JM, editor. PLoS Negl Trop Dis [Internet]. 2016 Jan 28 [cited 2019 Sep 23];10(1):e0004380. Available from: https://dx.plos.org/10.1371/journal.pntd.0004380

65. Basuni M, Rahumatullah A, Miswan N, Ahmad M, Noordin R, Verweij JJ, et al. A Pentaplex Real-Time Polymerase Chain Reaction Assay for Detection of Four Species of Soil-Transmitted Helminths. Am J Trop Med Hyg [Internet]. 2011 Feb 4 [cited 2019 Sep 23];84(2):338–43. Available from: http://www.ncbi.nlm.nih.gov/pubmed/21292911

66. Verweij JJ, Canales M, Polman K, Ziem J, Brienen EAT, Polderman AM, et al. Molecular diagnosis of Strongyloides stercoralis in faecal samples using real-time PCR. Trans R Soc Trop Med Hyg [Internet]. 2009 Apr [cited 2023 Jan 21];103(4):342–6. Available from: https://pubmed.ncbi.nlm.nih.gov/19195671/

67. Pastor AF, Silva MR, dos Santos WJT, Rego T, Brandão E, de-Melo-Neto OP, et al. Recombinant antigens used as diagnostic tools for lymphatic filariasis. Parasites and Vectors [Internet]. 2021 Dec 1 [cited 2023 Jan 21];14(1):1–14. Available from: https://parasitesandvectors.biomedcentral.com/articles/10.1186/s13071-021-04980-3

68. Dana D, Vlaminck J, Ayana M, Tadege B, Mekonnen Z, Geldhof P, et al. Evaluation of copromicroscopy and serology to measure the exposure to Ascaris infections across age groups and to assess the impact of 3 years of biannual mass drug administration in Jimma Town, Ethiopia. Gilleard JS, editor. PLoS Negl Trop Dis [Internet]. 2020 Apr 13 [cited 2020 Jun 3];14(4):e0008037. Available from: https://dx.plos.org/10.1371/journal.pntd.0008037

69. Mounsey K, Kearns T, Rampton M, Llewellyn S, King M, Holt D, et al. Use of dried blood spots to define antibody response to the Strongyloides stercoralis recombinant antigen NIE. Acta Trop. 2014 Oct 1;138:78–82.

70. Thompson K, Hutchins H, Baio A, Cassama E, Nabicassa M, Bailey R, et al. Health Beliefs and Perceptions of Trachoma in Communities on the Bijagos Archipelago of Guinea Bissau. Ophthalmic Epidemiol [Internet]. 2015 May 4 [cited 2018 Mar 10];22(3):190–9. Available from: http://www.tandfonline.com/doi/full/10.3109/09286586.2015.1036889

71. CONSORT Group. CONSORT Statement [Internet]. 2010 [cited 2022 Sep 23]. Available from: https://www.consort-statement.org/

72. WMA Declaration of Helsinki – Ethical Principles for Medical Research Involving Human Subjects – WMA – The World Medical Association [Internet]. [cited 2020 Apr 14]. Available from: https://www.wma.net/policies-post/wma-declaration-of-

For peer review only

For peer review only - http://bmjopen.bmj.com/site/about/guidelines.xhtml

helsinki-ethical-principles-for-medical-research-involving-human-subjects/
73. ICH. ICH GCP - ICH harmonised guideline integrated addendum to ICH E6(R1): Guideline for Good Clinical Practice ICH E6(R2) ICH Consensus Guideline [Internet]. Good Clinical Practice Network. 2020 [cited 2020 Jun 25]. Available from: https://ichgcp.net/
74. Public Domain. Map of Guinea-Bissau. Commons Wikimedia.

FIGURE LEGENDS

Figure 1: Timeline of the MATAMAL Clinical Trial showing months of Mass Drug Administration (MDA), cohort surveys, entomological surveys (Ento), major cross-sectional surveys (Survey) and Neglected Tropical Disease survey (NTD). Rainy season months indicated in dark colour.

Figure 2: Map of Guinea-Bissau, showing the Bijagos Archipelago off the south-west coast.[74]

Figure 3: Map of the Bijagos Archipelago. MATAMAL clusters marked by stars and separated by lines where sharing a landmass.

Figure 4: Design elements of the MATAMAL clinical trial.

Figure 5: Graph showing modelled qPCR prevalence of malaria over time, assuming 70% coverage and 75% dihydroartemisinin-piperaquine (DP) efficacy. Green dots: Mass Drug Administration (MDA) rounds. Green bars: survey periods. Dashed line: no intervention. Blue line: MDA with DP only. Pink line: MDA with DP and ivermectin at 300µg/Kg.

For peer review only

For peer review only - http://bmjopen.bmj.com/site/about/guidelines.xhtml

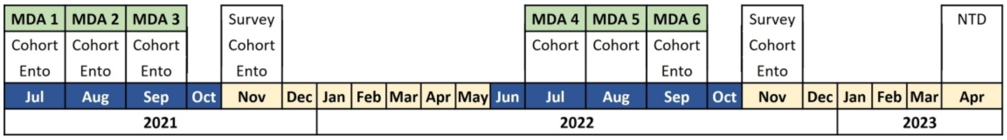

Figure 1: Timeline of the MATAMAL Clinical Trial showing months of Mass Drug Administration (MDA), cohort surveys, entomological surveys (Ento), major cross-sectional surveys (Survey) and Neglected Tropical Disease survey (NTD). Rainy season months indicated in dark colour

230x32mm (300 x 300 DPI)

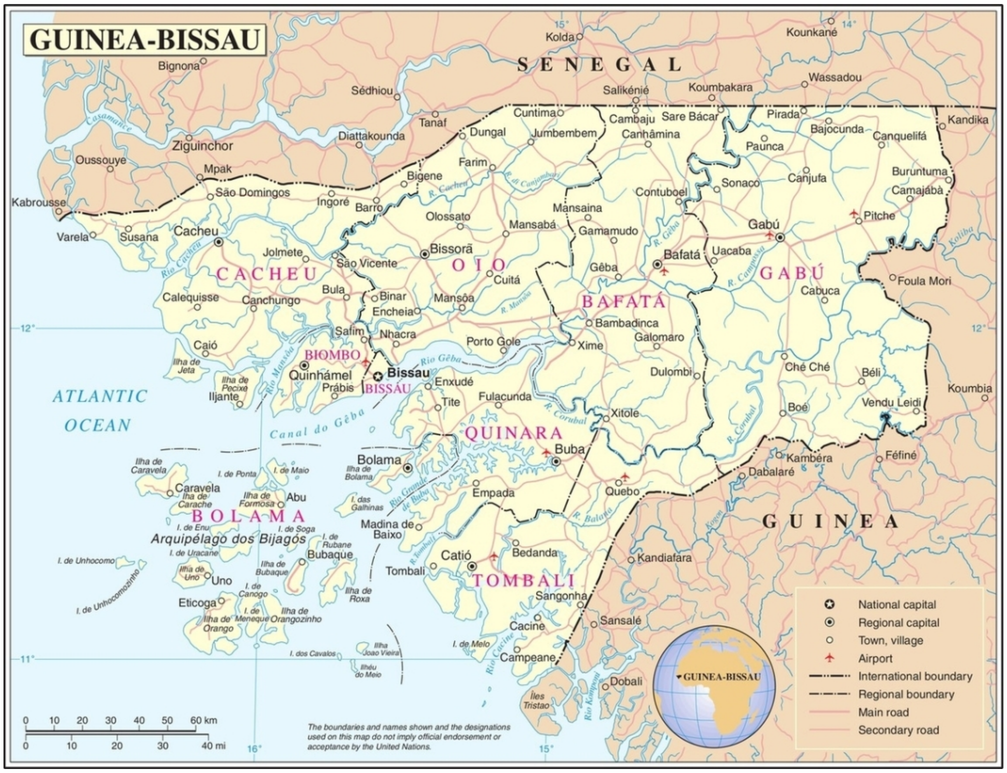

Figure 2: Map of Guinea-Bissau, showing the Bijagos Archipelago off the south-west coast.[73]

102x78mm (300 x 300 DPI)

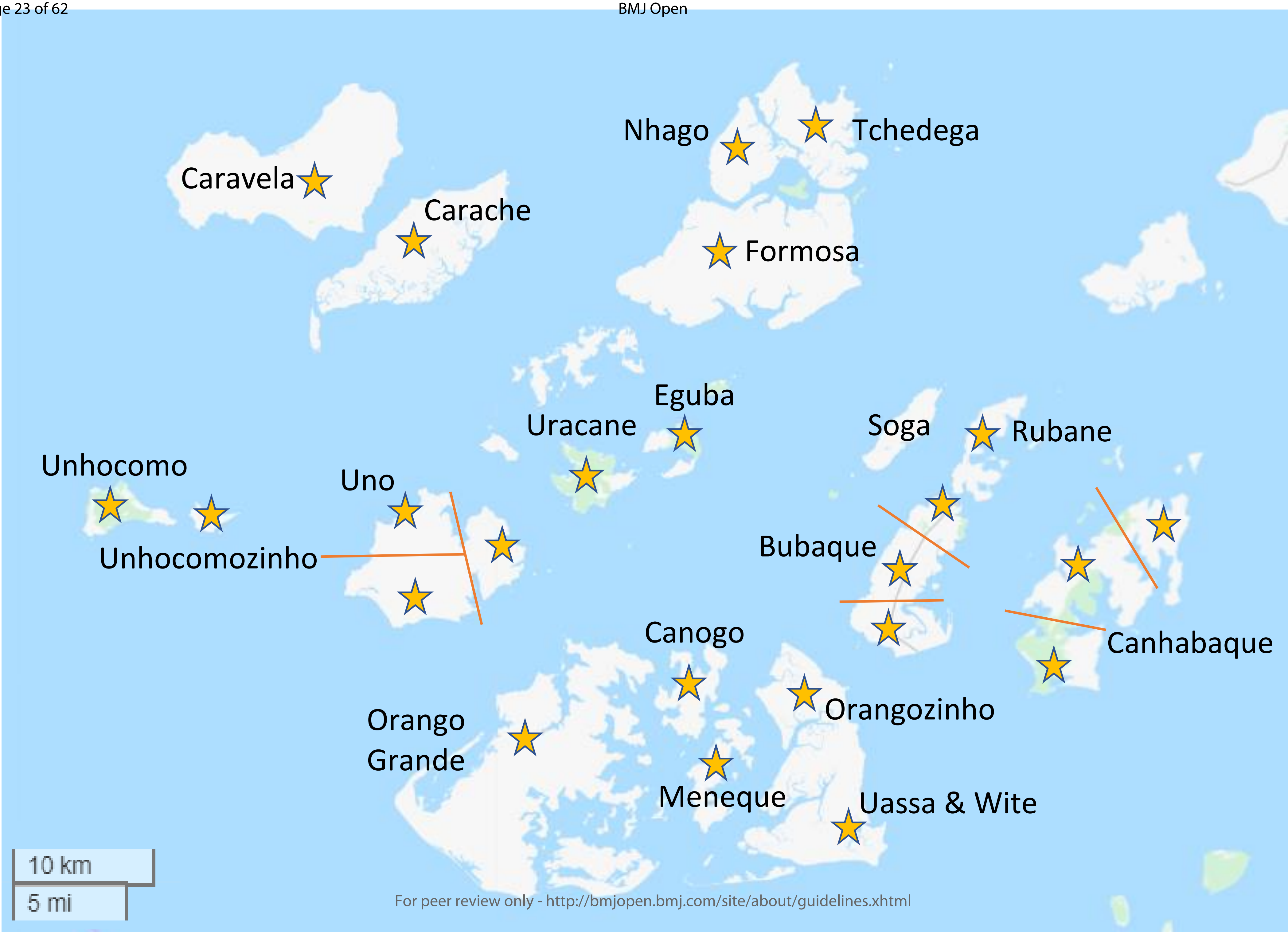
Caravela
Carache
Nhago
Tchedega
Formosa
Eguba
Uracane
Soga
Rubane
Unhocomo
Uno
Unhocomozinho
Bubaque
Canhabaque
Canogo
Orangozinho
Orango
Grande
Meneque
Uassa & Wite
10 km
5 mi

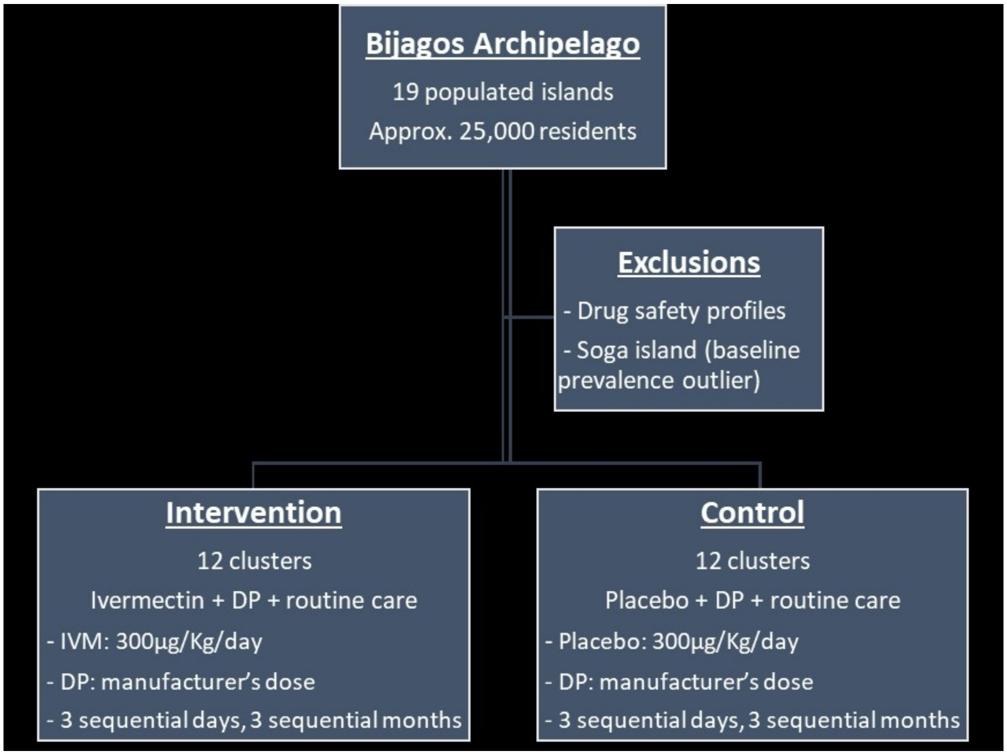

Figure 4: Design elements of the MATAMAL clinical trial.

146x109mm (150 x 150 DPI)

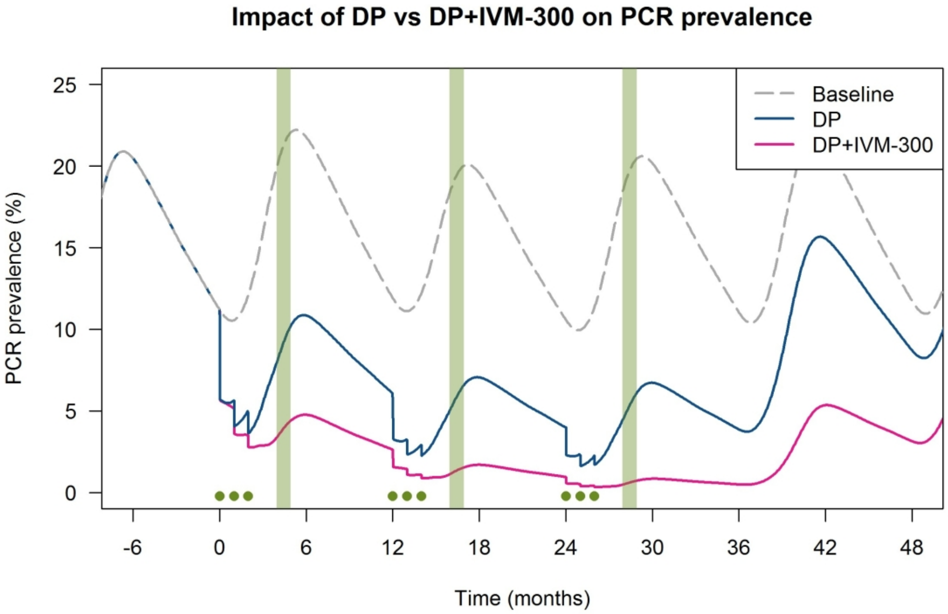

Figure 5: Graph showing modelled qPCR prevalence of malaria over time, assuming 70% coverage and 75% dihydroartemisinin-piperaquine (DP) efficacy. Green dots: Mass Drug Administration (MDA) rounds. Green bars: survey periods. Dashed line: no intervention. Blue line: MDA with DP only. Pink line: MDA with DP and ivermectin at 300µg/Kg.

199x134mm (300 x 300 DPI)

PARTICIPANT INFORMATION SHEET (ADULT)

Study Title:

MATAMAL

Ivermectin Mass Drug Administration for Malaria Control on the Bijagós Archipelago of Guinea Bissau: A cluster randomised placebo-controlled trial

Sponsor & Funder: The Joint Global Health Trials Scheme (DFID / NIHR / MRC / Wellcome Trust) is funding the study and LSHTM is the sponsor.

What is informed consent?

You are invited to take part in a research study. Before you decide you need to understand why the research study is being done and what it will involve. Please take time to read the following information or get the information explained to you in your language. Listen carefully and feel free to ask if there is anything that is not clear or you do not understand. You may also wish to consult your spouse, family members, friends or others before deciding to take part in the study.

If you decide to join the study, you will need to sign or put a thumbprint on a consent form saying that you agree to be in the study.

Why is this study being done?

This study is being done to assess the impact of a new approach to prevent malaria by using two different medicines, which will be given to everyone on the islands. As you may know, malaria in several African countries has declined substantially over the past few years, because of control measures like bed nets and use of medicines to prevent people from getting malaria or to treat people when they become ill with malaria. Even though many people are using these interventions, people on the island still get sick with malaria. To prevent malaria, we need to find extra control interventions. One of these interventions could be mass drug administration, which means giving medicines (tablets) to everyone from every community on the islands. We are going to use two medicines. One is called dihydroartemisinin-piperaquine (DP). This is an antimalarial drug that will kill the parasite that causes malaria if it is in your blood. This has worked well to reduce malaria in other countries in West Africa. The other drug is called ivermectin (IVM). When a person takes IVM, their blood can kill the mosquitoes that transmit malaria when they bite you. IVM is a drug that is also used to kill other parasites that infect people (such as some intestinal worms, headlice and bedbugs). We want to see if giving treatment to the whole community with these two drugs is better than just giving the whole community DP at reducing malaria on the islands. We also want to find out whether giving IVM can treat other parasites in addition to reducing malaria.

For peer review only

What does this study involve?

We are working in all of the villages on all the islands in the archipelago. We have done previous surveys on the islands to measure the proportion of people with malaria infection. All the islands will be included in this study. People from every island will be asked to participate in the study and will be given the two medicines described above. Half the islands will be given a placebo ('sugar' or 'dummy') medicine instead of ivermectin. The placebo medicine does not contain any drug of any kind. It looks exactly the same as the 'real' ivermectin medicine. The islands that are given the placebo medicine have been chosen randomly (by chance). Everyone will receive medication that looks exactly the same. Neither the study team nor you will know which medicine you will receive (placebo or ivermectin). Dihydroartemesinin-piperaquine will be given to everyone. The reason that we are using a placebo medicine is so that we can see whether it is better to ivermectin MDA with DP MDA or whether DP alone is good enough. All medicines will be given according to body weight or height. The medicine will be given to you once each day (by mouth with water and without food) for three days each month in June, July and August every year for two years. The medicine should be taken at least three hours after you last ate food and no food should be eaten for three hours after each dose. If you are unable to swallow the tablets, the medicines may be crushed and mixed with water. The mixture will be used immediately after preparation. If you vomit within 30 minutes of taking the medicine, the whole dose will be given again, but if vomiting occurs after 30-60 minutes, only half the dose will be given again. All the treatment will be directly supervised by our study team. All the islands will also receive the malaria programme control measures provided by the Bissau-Guinean Ministry of Public Health (bed nets, preventative treatment in pregnancy and antimalarial treatment if anyone becomes unwell with malaria).

We will conduct surveys for the presence of malaria and other diseases in your communities. Participants in these surveys will be examined by trained health care members of the study research team. We will conduct one survey each year after the rainy season in October/November. We will conduct an additional survey for other diseases such as scabies and worms in February. During the surveys, a questionnaire will be used to collect information on whether you slept under a bed net the previous night. You will also be asked whether you have travelled recently. Questions will also be asked about how and whether you took the medicines given during the mass drug administration. In addition, several droplets of blood will be taken from your finger onto a filter paper to see if there are parasites in his/her blood. This test will be done later in the MRC laboratory in The Gambia. However, if you have fever or recent history of fever, a malaria rapid diagnostic test will be done at the time to check if he/she is sick with malaria. If this is positive, he/she will receive treatment for malaria at that time.

During the course of the study, you will be encouraged to visit your nearest health post or talk to the ASC responsible for your tabanca if you feel sick with a fever. At the health facility a finger prick will be done for a malaria rapid diagnostic test. If the rapid diagnostic test is positive,

For peer review only

additional droplets of blood will be collected onto a filter paper and slide for malaria tests in the MRC laboratory in The Gambia and you will be treated with artemether-lumefantrine according to national guidelines.

Your house may be selected to house a special light trap to capture mosquitoes for six nights each month during the intervention and surveys. This will be like having a low level lamp on in your house during the night.

The following three paragraphs of information are relevant to you if you have children, for whom you will be asked to provide consent on their behalf on a separate form.

*Most cases of malaria happen in children between the ages of five to fourteen years. During the rainy season we will randomly select 50 children from each island in this age group and our study team will visit them each month until the end of the transmission season (six months from June-November). Your child may be selected to participate in this cohort (group). The team will complete a short questionnaire, take your child's temperature and take a finger prick blood sample to collect several droplets of blood onto a filter paper to see if there are parasites in his/her blood. This test will be done later in the MRC laboratory in The Gambia. However, if your child has a fever or recent history of fever, a malaria rapid diagnostic test will be done at the time to check if he/she is sick with malaria. If this is positive, he/she will receive treatment for malaria at that time and a blood film will be taken to look for parasites under the microscope.

*The medicine ivermectin also has additional benefits because it also treats common infestations and worms that we see in children. To see its effect on these infections, we will randomly select 100 children from each island, between the ages of four to thirteen years, the age group at highest risk of these infections. Your child may be selected for this assessment and will participate in a survey that will be done to estimate the prevalence of these common infections including parasites that affect the external body surface, scabies and worms. Bedbugs will be detected by visual inspection of the beds for 10 minutes using torchlight. Bed nets, mattresses (if present), bed frames and the wall next to the bed will be searched for the presence of eggs, immature and adult bedbugs. Head lice will be detected by a visual search of the scalp. The field assistant will sit with a white cloth across her/his lap and search the child's scalp before and after combing with a fine-toothed white nit comb. The comb and sheet will be inspected for the presence of lice and will take about 10 minutes to complete. Hair length will also be recorded. Scabies will be identified from a complete physical examination of the body and additionally, lesions will be examined for secondary infection. Skin swabs may be taken if there is any evidence of secondary skin infection. The examination will take approximately 10 minutes to complete. A stool sample will be taken to identify the presence of worms. All of the lab tests will be done at the MRC laboratory in The Gambia up to several months after the survey.

For peer review only

*If our study team find that your child has an infection that needs treatment at that time, our clinical team will dispense treatment or arrange for treatment at your health post. If our study team discovers that your child is sick and decides that your child cannot participate in the study because of that, your child will receive immediate care at the study site and then be referred to the appropriate health facility.

If the research study needs to be stopped for any reason, you will be informed and you and/or your child will have normal medical care.

What will happen to the samples taken in this study?

The blood samples will be used to determine whether malaria parasites are present in your blood. In addition, we will do tests to study how your body reacts to malaria parasites (immune tests) that will tell us whether you have had recent or past malaria exposure. These tests will be done in the MRC laboratory The Gambia but for quality control a selection of samples may be sent abroad to collaborating laboratories. Samples are labelled with unique identifying labels, so the laboratory technicians will not have any personal information about the sample. Stool samples will be used to test for the presence of worms. Additional stool tests for immune responses may be sent abroad to collaborators. Additional tests for infection and resistance to the medicines that we are using in this study may be carried out on stored blood samples from this study.

What harm or discomfort can you expect in the study?

All risks associated with the trial are low or moderate. You may experience discomfort from the finger prick blood samples we are taking. Sterile equipment will be used to take these samples. The medications used in this study are safe for use in this setting. Individuals to be treated will be advised to take the medicines as per manufacturer's instructions. Women in the first trimester of pregnancy or infants under 12 months of age will not be given dihydroartemisinin-piperaquine. Children less than 90 cm in height (or weighing less than 15kg), pregnant or breastfeeding women will not be treated with ivermectin or placebo. All participants will be monitored for any possible adverse events that may occur during the trial.

What benefits can you expect in the study?

This is the first large trial investigating the impact of ivermectin MDA with dihydroartemisinin-piperaquine MDA on malaria. Your participation in this trial is providing essential information to researchers and health care programmes so that we can establish whether ivermectin MDA will be successful in reducing malaria in your communities. If it does, ivermectin MDA might be able to accelerate the path towards malaria elimination on the Bijagos Islands and in other African countries where malaria is a big problem.

Will you be compensated for your participation in the study?

You will not get paid for your participation. In case the study clinician feels it is in your interest to visit a health facility, you will either get transport provided by the study team or get the costs for the transport reimbursed.

For peer review only

A clinical assessment and simple treatment may be provided by the study team in the field before referral.

What happens if you decide not to participate in the study or change your mind later?

You are free to decide whether to participate or not in the study and you have the right to stop participating at any time without giving a reason. This will not affect the medical care that you would normally receive. In case you decide to withdraw your participation during the study, any information already generated from the samples until the time of withdrawal will be used and samples already collected, for which you have given consent, will also be analysed and data used. The study doctor may also ask for tests for your safety.

Should any new information become available during the study that may affect your participation, you will be informed as soon as possible.

What compensation will be available if you are injured during the study?

We will be responsible for the provision of treatment for injuries caused by procedures related to the research study. If medical treatment is required as an emergency, please go directly to your health centre or clinic and contact the field assistant who gave his/her telephone number to you or contact Mrs Eunice Teixeira e Silva Cassama on +245 96 6288282. In addition, LSHTM carries Clinical Trial/Non-Negligent Harm Insurance and Medical Malpractice Insurance applicable to this study.

How will your information be kept and who will be allowed to see it?

All information that is collected about you in the course of the study will be kept strictly confidential. Your personal information will only be available to the study team members and might be seen by some rightful persons from the Ethics Committees, the sponsor and for confidential external monitoring purposes.

At the end of the project, the study data will be archived in London. The data will be made available to other researchers worldwide for research and to improve medical knowledge and patient care. Your personal information will not be included and there is no way that you can be identified, as these data will have been de-identified, or 'de-personalised', meaning that we have removed any personal identifiers, before making the data available for use in the circumstances above.

Who should you contact if you have questions?

If you have any queries or concerns you can contact Dr Harry Hutchins on +245 96 6871504 or Mrs Eunice Teixeira e Silva Cassama on +245 96 6288282 and you can always call the mobile phone numbers of the study staff that are given to you.

Please feel free to ask any question you might have about the research study.

For peer review only

Who has reviewed this study?

This study has been reviewed and approved by the Comite de Eticas na Saude in Bissau and the Ethics Committee at the London School of Hygiene and Tropical Medicine (UK). It has also been reviewed by a panel of scientists at the Medical Research Council The Gambia Unit. These committees review such studies to protect your rights and wellbeing before approving them.

Thank you for taking time to read this information leaflet. If you agree to take part in the study please read and sign the consent form in the presence of our researcher.

For peer review only

MATAMAL v1.0 22/01/2020

CONSENT FORM (CHILD under the age of 12 years)

MATAMAL (CONSENT TO MASS DRUG ADMINISTRATION)

Ivermectin Mass Drug Administration for Malaria Control on the Bijagós Archipelago of Guinea Bissau: A cluster randomised placebo-controlled trial

A study conducted by the London School of Hygiene and Tropical Medicine (UK), The Ministry of Public Health (Guinea Bissau), The National Institute of Public Health (INASA), The Bandim Health Project and MRC @ LSHTM The Gambia Unit (The Gambia).

(Principal Investigator/Responsible Researcher: Dr Anna Last)

Participant's Name ____

Participant's Identification Number: |__|__|__|__|__|__|__|__|__|__|

______________________ **OR** ______________________

(Printed name of parent) (Printed name of guardian)

Tick as appropriate

☐ I have read the written information **OR**
☐ I have had the information explained to me by study personnel in a language that I understand
☐ I confirm that my choice for my child to participate is entirely voluntary and that I can withdraw consent at any time
☐ I confirm that I have had the opportunity to ask questions about this study and I am satisfied with the answers and explanations that have been provided by the study team
☐ I understand that I am allowing access to information about/from my child to authorised persons described in the information sheet
☐ I understand that information about/from my child may be shared on a public data repository or with other researchers but that I will not be identifiable from this information
☐ I have received sufficient time to consider my child's participation in this study
☐ I agree to my child taking part in this study

Participant's parent or guardian's signature/thumbprint* [ ] ______________________

Date (dd/mmm/yyyy) Time (24hr)

Printed name of witness* ______________________

Printed Name of Person obtaining consent ______________________

I attest that I have explained the study information accurately in ______________________ (*insert language*) to, and was understood to the best of my knowledge by, the participant/parent/guardian. He/she has freely given consent to participate in the presence of the above named witness*.

Signature of Person obtaining consent ______________ ______________________

Date (dd/mmm/yyyy) Time (24hr)

* *Only required if the participant is unable to read or write*

A copy of this informed consent document has been provided to the participant

For peer review only

MATAMAL v1.0 22/01/2020

ASSENT FORM (12-17 years)

MATAMAL (ASSENT TO MASS DRUG ADMINISTRATION)

Ivermectin Mass Drug Administration for Malaria Control on the Bijagós Archipelago of Guinea Bissau: A cluster randomised placebo-controlled trial

A study conducted by the London School of Hygiene and Tropical Medicine (UK), The Ministry of Public Health (Guinea Bissau), The National Institute of Public Health (INASA), The Bandim Health Project and MRC @ LSHTM The Gambia Unit (The Gambia).

(Principal Investigator/Responsible Researcher: Dr Anna Last)

Participant's Name ____

Participant's Identification Number: |__|__|__|__|__|__|__|__|__|__|

Tick as appropriate

☐ I have read the written information **OR**
☐ I have had the information explained to me by study personnel in a language that I understand
☐ I confirm that my choice to participate is entirely voluntary and that I can withdraw consent at any time
☐ I confirm that I have had the opportunity to ask questions about this study and I am satisfied with the answers and explanations that have been provided by the study team
☐ I understand that I am allowing access to information about/from me to authorised persons described in the information sheet
☐ I understand that information about/from me may be shared on a public data repository or with other researchers but that I will not be identifiable from this information
☐ I have received sufficient time to consider my participation in this study
☐ I agree to take part in this study

Participant's signature/thumbprint* ____________________ ____________________

Date (dd/mmm/yyyy) Time (24hr)

Printed name of parent or guardian ____

Signature/thumbprint of parent or guardian* ____________________ ____________________

Date (dd/mmm/yyyy) Time (24hr)

Printed name of witness* ____

Printed Name of Person obtaining consent ____

I attest that I have explained the study information accurately in ______________________ (*insert language*) to, and was understood to the best of my knowledge by, the participant/parent/guardian. He/she has freely given consent to participate in the presence of the above named witness*.

Signature of Person obtaining consent ____________________ ____________________

Date (dd/mmm/yyyy) Time (24hr)

** Only required if the participant is unable to read or write*

A copy of this informed consent document has been provided to the participant

For peer review only

MATAMAL v1.0 22/01/2020

CONSENT FORM (ADULT)

MATAMAL (CONSENT TO MASS DRUG ADMINISTRATION)

Ivermectin Mass Drug Administration for Malaria Control on the Bijagós Archipelago of Guinea Bissau: A cluster randomised placebo-controlled trial

A study conducted by the London School of Hygiene and Tropical Medicine (UK), The Ministry of Public Health (Guinea Bissau), The National Institute of Public Health (INASA), The Bandim Health Project and MRC @ LSHTM The Gambia Unit (The Gambia)

(Principal Investigator/Responsible Researcher: Dr Anna Last)

Participant's Name ____

Participant's Identification Number: |__|__|__|__|__|__|__|__|__|__|

Tick as appropriate

☐ I have read the written information **OR**
☐ I have had the information explained to me by study personnel in a language that I understand
☐ I confirm that my choice to participate is entirely voluntary and that I can withdraw consent at any time
☐ I confirm that I have had the opportunity to ask questions about this study and I am satisfied with the answers and explanations that have been provided by the study team
☐ I understand that I am allowing access to information about/from me to authorised persons described in the information sheet
☐ I understand that information about/from me may be shared on a public data repository or with other researchers but that I will not be identifiable from this information
☐ I have received sufficient time to consider my participation in this study
☐ I agree to take part in this study

Participant's signature/thumbprint* ______________ ______________

Date (dd/mmm/yyyy) Time (24hr)

Printed name of witness* ____

Printed Name of Person obtaining consent ____

I attest that I have explained the study information accurately in _______________________ (*insert language*) to, and was understood to the best of my knowledge by, the participant/parent/guardian. He/she has freely given consent to participate in the presence of the above named witness*.

Signature of Person obtaining consent ______________ ______________

Date (dd/mmm/yyyy) Time (24hr)

** Only required if the participant is unable to read or write.*

A copy of this informed consent document has been provided to the participant

For peer review only - http://bmjopen.bmj.com/site/about/guidelines.xhtml

MDA Administration Record

MATAMAL

Cluster	Island	Village	Head of Family, Name	Head of Family, Nickname	Year
					2021 2022

Number	Name	Surname	Age	Sex	Consent			Month 1			Month 2			Month 3		
				M F	DP eligible			1	2	3	1	2	3	1	2	3
			Weight		IVM eligible		**DP**									
			________ Kg		Pregnant		**IVM**									
Number	Name	Surname	Age	Sex	Consent			Month 1			Month 2			Month 3		
				M F	DP eligible			1	2	3	1	2	3	1	2	3
			Weight		IVM eligible		**DP**									
			________ Kg		Pregnant		**IVM**									
Number	Name	Surname	Age	Sex	Consent			Month 1			Month 2			Month 3		
				M F	DP eligible			1	2	3	1	2	3	1	2	3
			Weight		IVM eligible		**DP**									
			________ Kg		Pregnant		**IVM**									
Number	Name	Surname	Age	Sex	Consent			Month 1			Month 2			Month 3		
				M F	DP eligible			1	2	3	1	2	3	1	2	3
			Weight		IVM eligible		**DP**									
			________ Kg		Pregnant		**IVM**									

For peer review only

Number	Name	Surname	Age	Sex	Consent			Month 1			Month 2			Month 3		
				M F	DP eligible			1	2	3	1	2	3	1	2	3
			Weight		IVM eligible		DP									
			________ Kg		Pregnant		IVM									

Number	Name	Surname	Age	Sex	Consent			Month 1			Month 2			Month 3		
				M F	DP eligible			1	2	3	1	2	3	1	2	3
			Weight		IVM eligible		DP									
			________ Kg		Pregnant		IVM									

Number	Name	Surname	Age	Sex	Consent			Month 1			Month 2			Month 3		
				M F	DP eligible			1	2	3	1	2	3	1	2	3
			Weight		IVM eligible		DP									
			________ Kg		Pregnant		IVM									

Number	Name	Surname	Age	Sex	Consent			Month 1			Month 2			Month 3		
				M F	DP eligible			1	2	3	1	2	3	1	2	3
			Weight		IVM eligible		DP									
			________ Kg		Pregnant		IVM									

Number	Name	Surname	Age	Sex	Consent			Month 1			Month 2			Month 3		
				M F	DP eligible			1	2	3	1	2	3	1	2	3
			Weight		IVM eligible		DP									
			________ Kg		Pregnant		IVM									

Number	Name	Surname	Age	Sex	Consent			Month 1			Month 2			Month 3		
				M F	DP eligible			1	2	3	1	2	3	1	2	3
			Weight		IVM eligible		DP									
			________ Kg		Pregnant		IVM									

For peer review only

MATAMAL

Adjunctive Ivermectin Mass Drug Administration for Malaria Control: A cluster-randomised placebo-controlled trial

Standard Operating Procedure<br>Finger Prick Blood Sampling for Dry Blood Spot Collection	
SOP Ref:	MATAMAL/SOP/C9
Version:	1.0
Authors:	Harry Hutchins
Effective Date:	
Review by:	
Approved by:	
Approval Date:	
Signed by:	

SOP Chronology

Version	Date	Reason for Change	Author
1.0		N/A	

For peer review only - http://bmjopen.bmj.com/site/about/guidelines.xhtml

For peer review only

Table of Contents	Page
1. Purpose	3
2. Policy Statement	3
3. Background	3
4. Scope	3
5. Responsibilities	3
6. Equipment	3
7. Procedure	4
8. References	5
9. Appendices	

For peer review only

For peer review only - http://bmjopen.bmj.com/site/about/guidelines.xhtml

Purpose

This Standard Operating Procedure (SOP) describes the technique for obtaining uniform and high-quality dry blood spot (DBS) samples to facilitate laboratory analysis and reduce harm to participants.

Policy Statement

All LSHTM SOPs will be produced and approved in accordance with the LSHTM SOP on SOPs. All studies sponsored or hosted by LSHTM, including within the LSHTM Clinical Trials Unit, should comply with these procedures and must be used in conjunction with overarching LSHTM policies and guidelines and all relevant legal and ethical requirements governing the conduct of the study.

Background

DBS have been an important tool for sample collection, especially in infectious disease and metabolic medicine, for over a century. They provide a robust medium for transport and preservation of clinical samples from even remote and hostile environments. They are low-cost, simple to produce and collect, and can be used for complex laboratory analysis.[1,2]

Development of techniques in serological and molecular analysis mean a simple DBS sample can now be used to identify and quantify a variety of infections as part of a single assay[3]. This can then be compared with other diagnostic techniques, timepoints and locations to improve understanding of disease epidemiology.

A structured SOP combined with effective training ensures that high-quality DBS are taken on properly prepared booklets, improving the likelihood of successful and accurate analysis on delivery to the laboratory.

Scope

This SOP applies to the collection of all DBS in the context of the MATAMAL trial.

Responsibilities

Clinician: informing participants and taking DBS using the correct method.

Field assistant: identifying participants and ensuring informed consent is in place. Safely storing DBS. Assisting the clinician as directed.

Equipment

- Informed consent/assent form
- Non-sterile gloves
- Hand sanitiser
- Clinical waste bag
- Sharps bin
- Alcohol wipe
- Lancet (paediatric or adult)
- Cotton wool balls
- Pre-made DBS booklet (see appropriate SOP)
- Zip-lock/sealable plastic bag or box
- Elastic band

For peer review only - http://bmjopen.bmj.com/site/about/guidelines.xhtml

- Silica gel (1g sachets)

Procedure[1,4]

1. Ensure informed consent is in place and explain procedure to participant.
2. Ensure sample ID is affixed to the DBS booklet and documented alongside participant ID.
3. Sanitise hands and don gloves.
4. It is important that no-one touches the filter paper without gloves.
5. Collect the sample:
    a. Clean the pulp of the 4th finger of the participants' non-dominant hand with an alcohol wipe. Allow to air dry.
    b. Use a lancet to prick the finger. Dispose of the lancet into a sharps bin. (Figure 1)
    c. Wipe the first bead of blood away with cotton wool.
    d. With the hand palm-down, exert gentle pressure to encourage bleeding and press the blood against the filter paper in three distinct circles. (Figure 2)
    e. Ensure enough blood in each circle to soak through to the other side. (Figure 3A, unlike Figure 3B)
    f. Allow samples to air dry to brown before folding securely.
6. Collect 10-25 DBS booklets together with an elastic band and place in a zip-lock bag or sealed box containing silica gel sachets. (Figure 4)
7. These DBS should ultimately be stored in -20°C freezers. They may be kept at -4°C or even ambient temperature for up to 14 days.
8. Discard all waste: sharps into sharps bin, other waste into clinical waste bag.

Figure 1	Figure 2
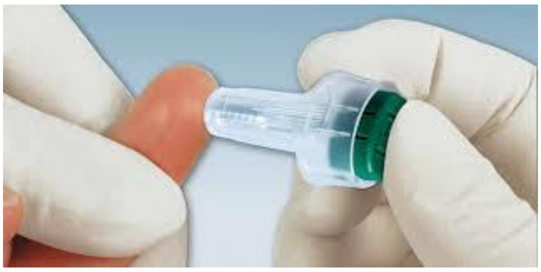	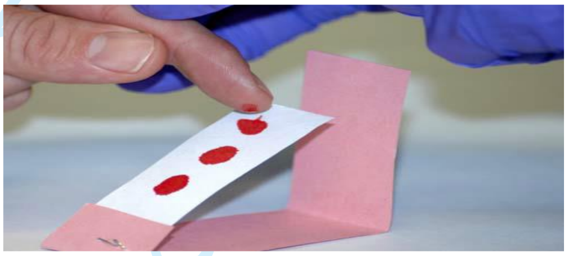
Figure 3	Figure 4
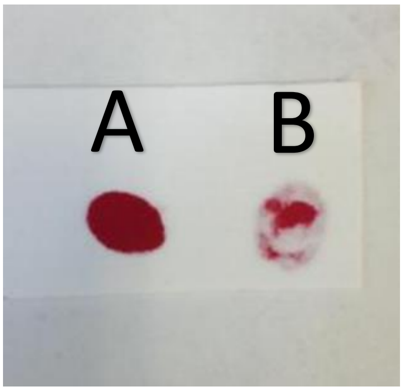	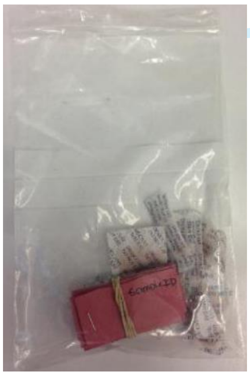

For peer review only - http://bmjopen.bmj.com/site/about/guidelines.xhtml

References

1. Grüner N, Stambouli O, Ross RS. Dried blood spots - Preparing and processing for use in immunoassays and in molecular techniques. *J Vis Exp*. 2015;2015(97). doi:10.3791/52619

2. Zakaria R, Allen KJ, Koplin JJ, Roche P, Greaves RF. Advantages and challenges of dried blood spot analysis by mass spectrometry across the total testing process. *JIFCC*. 2016;27(4):288-317.

3. Sanprasert V, Kerdkaew R, Srirungruang S, Charuchaibovorn S, Phadungsaksawasdi K, Nuchprayoon S. Development of Conventional Multiplex PCR: A Rapid Technique for Simultaneous Detection of Soil-Transmitted Helminths. *Pathogens*. 2019;8(3):152. doi:10.3390/pathogens8030152

4. Mei J V, Richard Alexander J, Adam BW, Harry Hannon W, Finley A. Use of Filter Paper for the Collection and Analysis of Human Whole Blood Specimens. *J Nutr*. 2001;131:1631S-1636S. https://academic.oup.com/jn/article-abstract/131/5/1631S/4686823. Accessed April 19, 2020.

For peer review only

MATAMAL
Adjunctive Ivermectin Mass Drug Administration for Malaria Control: A cluster-randomised placebo-controlled trial
Standard Operating Procedure

DNA extraction of dry blood spots (DBS)	
SOP Ref:	MATAMAL/SOP/J2
Version:	1.0
Authors:	Hristina Vasileva
Effective Date:	21.01.2020
Review by:	
Approved by:	
Approval Date:	
Signed by:	

Version	Date	Reason for Change
1.0	21.01.2020	N/A

For peer review only

For peer review only - http://bmjopen.bmj.com/site/about/guidelines.xhtml

Date: 21.01.2020

Equipment and Consumables:

- PureLink™ Pro 96 Genomic DNA purification Kit (cat No: K182104A)
- 96 well 0.8mL polypropylene deepwell storage plate (cat No: AB0765)
- Filter Tips (200µL; 1200µL)
- Multichannel pipette (200µL; 1200µL)
- 96 well plate centrifuge
- Water bath
- 200mL measuring cylinder/ 50mL Stripettes

Reagents:

- 99.9% pure Ethanol

Preparation of Buffer Solutions (according to PureLink™ Pro 96 Genomic DNA purification kit guidelines)

1. **Wash buffer 1:**
- Add 150mL of 99.9% pure Ethanol to the Wash Buffer 1 bottle and mix well.
- Indicate that ethanol is added and note the date on the bottle.
2. **Wash buffer 2:**
- Add 175mL of 99.9% pure Ethanol to the Wash Buffer 2 bottle and mix well. #
- Indicate that ethanol is added and note the date on the bottle.
3. **Digestion buffer*:**
- Add 20µL Proteinase K (PTK) to 180µL Genomic Digestion (GD) buffer per sample and mix well.
- Make as much buffer as needed each day and allow for 10% access as follow:
  - For 1x96 well plate: 2200µL PTK to 19800µL GD buffer
  - For 2x96 well plates: 4400µL PTK to 39600µL GD buffer
  - For 3x96 well plates: 6600µL PTK to 59400µL GD buffer
  - For 4x96 well plates: 8800µL PTK to 79200µL GD buffer
4. **Lysing binding buffer:**
- Add 200µL of 99.9% pure Ethanol (EtOH) to 200µL genomic lysing-binding (L-B) buffer per sample and mix well.
- Make as much buffer as needed each day and allow for 10% access as follow:
  - For 1x96 well plate: 22000µL EtOH to 22000µL L-B buffer
  - For 2x96 well plates: 44000µL EtOH to 44000µL L-B buffer
  - For 3x96 well plates: 66000µL EtOH to 66000µL L-B buffer
  - For 4x96 well plates: 88000µL EtOH to 88000µL L-B buffer

- ***If the digestion buffer appears stringy with small crystals, put it in a warm incubator for 30 minutes before using (or until the crystals have dissolved). The stringiness indicates that the temperature in the room where the kit is stored is below the normal room temperature.***

For peer review only

For peer review only - http://bmjopen.bmj.com/site/about/guidelines.xhtml

Laboratory Procedure

1. Prepare the needed buffers as described above.
2. Take up to 4 96 deep well plates containing punched DBS as described in MATAMAL SOP J1.
3. Switch on the water bath and set it to 55ºC.
4. Using filter tips and multichannel pipette, add 200µL of digestion buffer to each well to completely immerse the filter paper pieces (press with the tips if pieces and not immersed).
5. Change tips for each sample.
6. Seal the plate with foil tape provided in the kit and vortex the plate for 5 seconds.
7. Incubate the seal plate in the water bath (at 55ºC) for 30 minutes.
8. Centrifuge the plate at 2100g (RFC) for 3 minutes at room temperature (20ºC) to pellet the paper fibres.
9. In a new 96 deep well plate add 20µL RNase A per well and label it.
10. Add the 200µL of digest material without paper fibres to the 96 well plate containing the RNase A

- **Make sure you keep the order of the samples (eg: transfer A1 to A1, B1 to B1 etc).**

11. Prepare the 96 well column plate included in the kit on top of a fresh 96 deep well plate and label it.
12. Add 400µL of genomic lysis/binding buffer to each well containing the digest and the RNase A

- **Use the same tip to transfer the lysate to the assembled 96 well column plate.**
- **Make sure again that the sample order is kept while transferring.**

13. Seal the stacked plate with foil tape and centrifuge at 2100g for 10 minutes.
14. Discard the flow throw, collected in the deep well, and place the column plate back on top.
15. Add 500µL Wash buffer 1 into each well of the column plate
16. Centrifuge the stacked plate at 2100g for 5 minutes.
17. Discard the flow through and reassemble the stack.
18. Add 500µL Wash buffer 2 to each well of the column plate.
19. Centrifuge the stacked plate at 2100g for 5 minutes.
20. Discard the flow through and reassemble the stack.
21. Centrifuge the empty stack at 2100g for 5 minutes.
22. Discard the deep well pate and stack the column plate on top of a fresh 96 deep well plate.
23. Add 80µL of Elution buffer to each well and incubate on the bench for 10 minutes.
24. Centrifuge the stack at 2100g for 3 minutes.
25. Take the elution (flow through) and add it back on the column.
26. Reassemble the column on top of the same plate.

- **Very crucial step to not cross contaminate as the elution contains the DNA.**

27. Incubate at room temperature for 10 minutes.
28. Centrifuge at 2100g for 3 minutes.
29. Discard the column plate and seal the 96 deep well plate, containing the eluted DNA, with a foil tape.
30. Store the DNA at 4ºC (fridge) for short term storage (up to 2 weeks) or at -20ºC (freezer) for long term storage.

- **Avoid multiple freeze thawing.**

For peer review only

For peer review only - http://bmjopen.bmj.com/site/about/guidelines.xhtml

MATAMAL

Adjunctive Ivermectin Mass Drug Administration for Malaria Control: A cluster-randomised placebo-controlled trial

Standard Operating Procedure

Plasmodium falciparum VarATS **Quantifying Real-Time Polymerase Chain Reaction (qPCR).**	
SOP Ref:	MATAMAL/SOP/J3
Version:	1.0
Authors:	Hristina Vasileva
Effective Date:	21.04.2020
Review by:	
Approved by:	
Approval Date:	
Signed by:	

Version	Date	Reason for Change
1.0	21.04.2020	N/A

For peer review only

For peer review only - http://bmjopen.bmj.com/site/about/guidelines.xhtml

Date: 22.01.2020

Equipment and Consumables:

- Bench top tube centrifuge
- Vortex (SLS, Serial: 12030824)
- 1.5mL DNA LowBind Eppendorf tubes (cat No: 10031282)
- Filter tips (10µL, 20µL, 200µL, 1000µL)
- Multichannel micropipettes (P10; P50)
- Singlechannel micropipettes (P10; P20; P200; P1000)
- 96 well PCR plate (Life Sciences; cat No: 04729692001)
- MicroAmp optical adhesive film (Thermofisher Scientific; cat No: 4311971)
- 1.5mL DNA LoBind Eppendorf tubes (Eppendorf; cat No: 0030108051)
- Plate centrifuge (Benchmark, Serial: E081501133)
- Bio Rad CFX996 real-time PCR machine (Serial: 785BR13221)
- Mustang Purple Dye Spectral Calibration Plate, 96-weels (Life Technologies; cat No:4461599)

Reagents:

- TaqMan™ Universal Master Mix (Applied Biosystems; cat No: 4304437) stored at 4°C
- *P. falciparum varATS* forward primer (5'- CCCATACACAACCAAYTGGA -3') stored at -20°C
- *P. falciparum varATS* reverse primer (5'- TTCGCACATATCTCTATGTCTATCT -3') stored at -20°C
- *P. falciparum varATS* probe (6FAM- TRTTCCATAAATGGT-NFQ-MGB) stored at -20°C
- Nuclase-free water (Qiagen; cat No: 129114) *stored at 4°C
- Bleach (Madar)
- Ethanol (VWR; cat No: 20821.321)

For peer review only

Laboratory Procedure:

1. Generate a plate plan with corresponding sample IDs which should include the following and shown in Table 1:
   - Serial dilution standards with known parasitaemia (for trend-line and PCR positive control), refer to MATAMAL SOP J…
   -
2. Make 500µL of 10µM forward and reverse primers.
   - Original concentration of primers: 100µM
   - Dilution factor to make 10µM stock: 100/10=10
   - For 500µL primer mix use 500/10=50µL of each primer
   - Add 400µL of Nuclease-free water to make up the volume to 500µL
3. Make 500µL of 4µM FAM probe.
   - Original concentration of probe: 100µM
   - Dilution factor to make 4µM stoke: 100/4=25
   - For 500µL probe mix use 500/25=20µL of FAM probe
   - Add 480µL of Nuclease-free water to make the volume up to 500µL3.
4. Calculate volume of 10µM primers and 4µM probe needed for 1 qPCR reaction of 20µL.
   - TaqMan Multiplex Master mix: 10µL
   - 100nM/20µL primers: 10/0.2=50 (dilution factor); 20/50=0.4µL of 10µM stoke
   - 80nM/20µL probe: 4/0.2=50 (dilution factor); 20/50=0.4µL of 4µM stoke
   - Sample/Standards/Control DNA: 5µL
   - Nuclease-free water: 4.2µL
5. Working in a DNA hood, make master mix (excluding the sample volume) enough for 110 samples (to allow for excess) in a 2mL DNA LowBind Eppendorf.
   - TAqMan Multiplex Master Mix: 110*10=1100µL
   - Primers: 110*0.4=44µL of 10µM stock
   - Probe: 110*0.4=44µL of 4µM stock
   - Nuclease-free water: 110*4.2=462µL
6. Vortex the master mix for 10 seconds.
7. Using a repetitive pipette, pipette 15µL in each well of a 96 well optical plate.
8. Take everything out of the DNA hood and UV blast it.
9. On the working bench, pipette 5µL of Samples, Standards and Controls as shown in Figure1 and mix by aspirating up and down multiple times.

Figure1

For peer review only

For peer review only - http://bmjopen.bmj.com/site/about/guidelines.xhtml

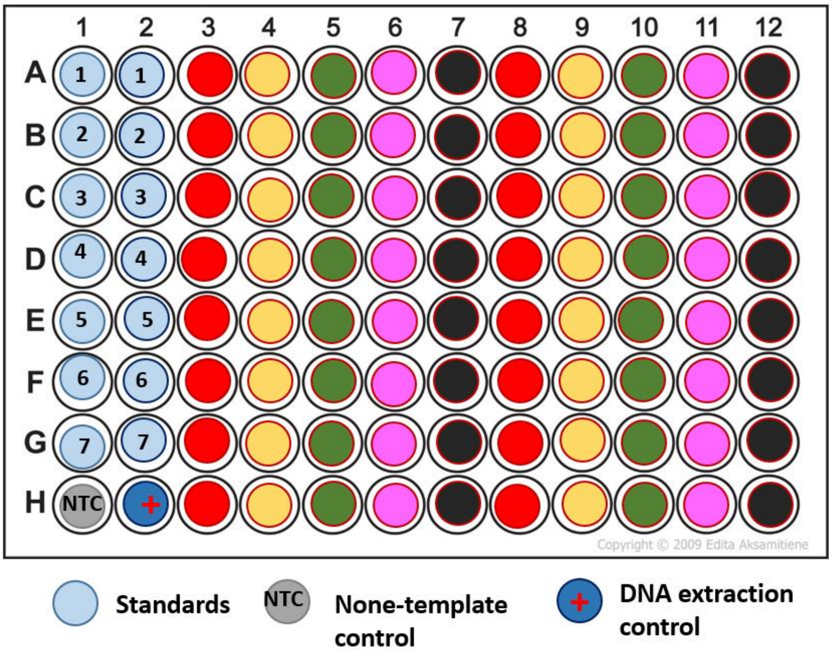

10. The grid of Figure1 shows the organization of samples and standards, where same colours indicate the same samples in duplicates
- Eg: Columns 3 and 8 contain the same samples in the exact same order.
- None-template control is 5µL of Nuclease-free water.
- DNA extraction control information is found in MATAMAL SOP J2.
11. Seal the plate with optical adhesive film and press around the edges.
12. Centrifuge the plate in the plate centrifuge for 5 seconds and make sure that there are no bubbles on the bottom of the wells.
13. Run the plate an Applied Biosystems 7500 Real-Time PCR machine at the absolute quantification mode.
14. Set the parameters of the run as shown below*.

- ***Ensure that MUSTANG PURPLE is selected as a reference dye. If MUSTANG PURPLE is not present as option, a calibration using the Mustang Purple Dye Spectral Calibration Plate must be performed.***
- ***Ensure that the amplification cycle number is set as 45, rather than the default set up of 40 cycles.***

For peer review only

For peer review only - http://bmjopen.bmj.com/site/about/guidelines.xhtml

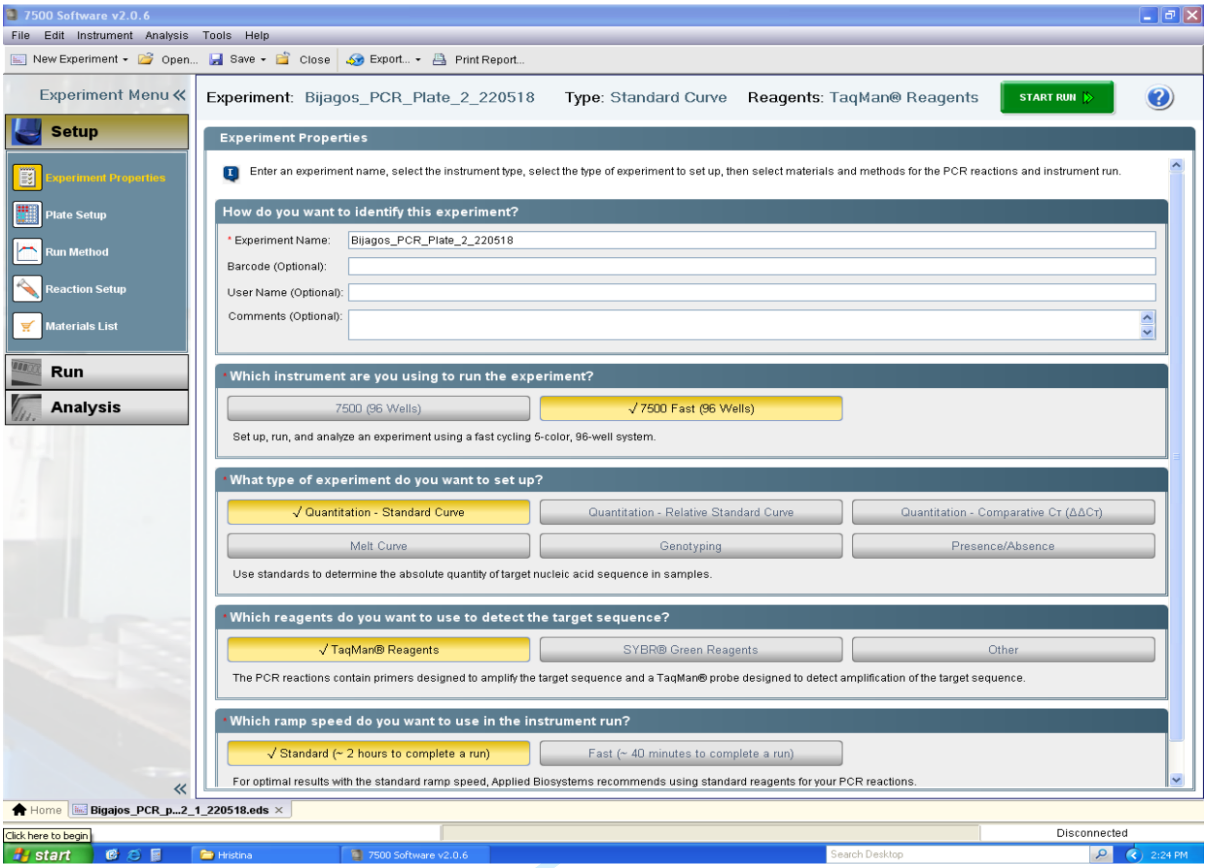

For peer review only

For peer review only - http://bmjopen.bmj.com/site/about/guidelines.xhtml

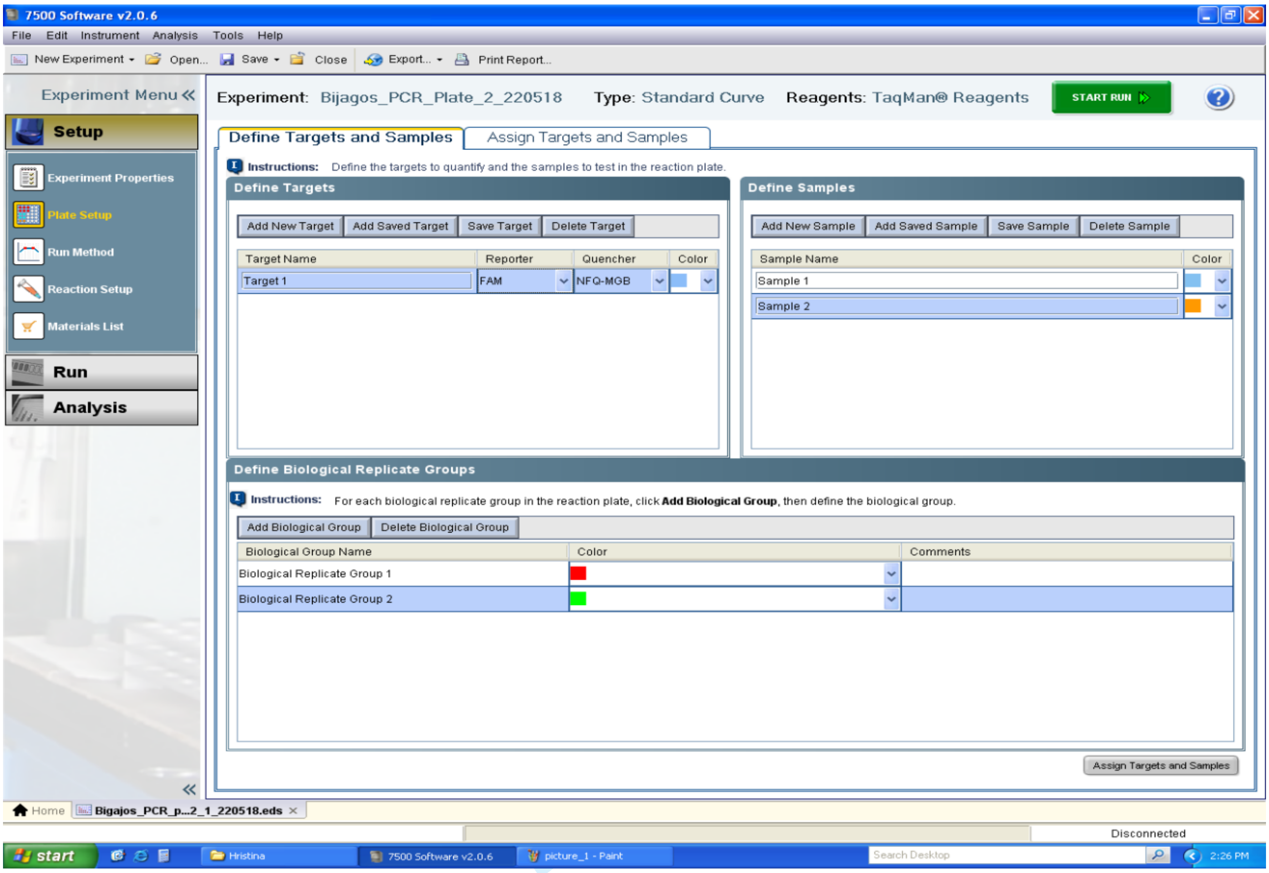

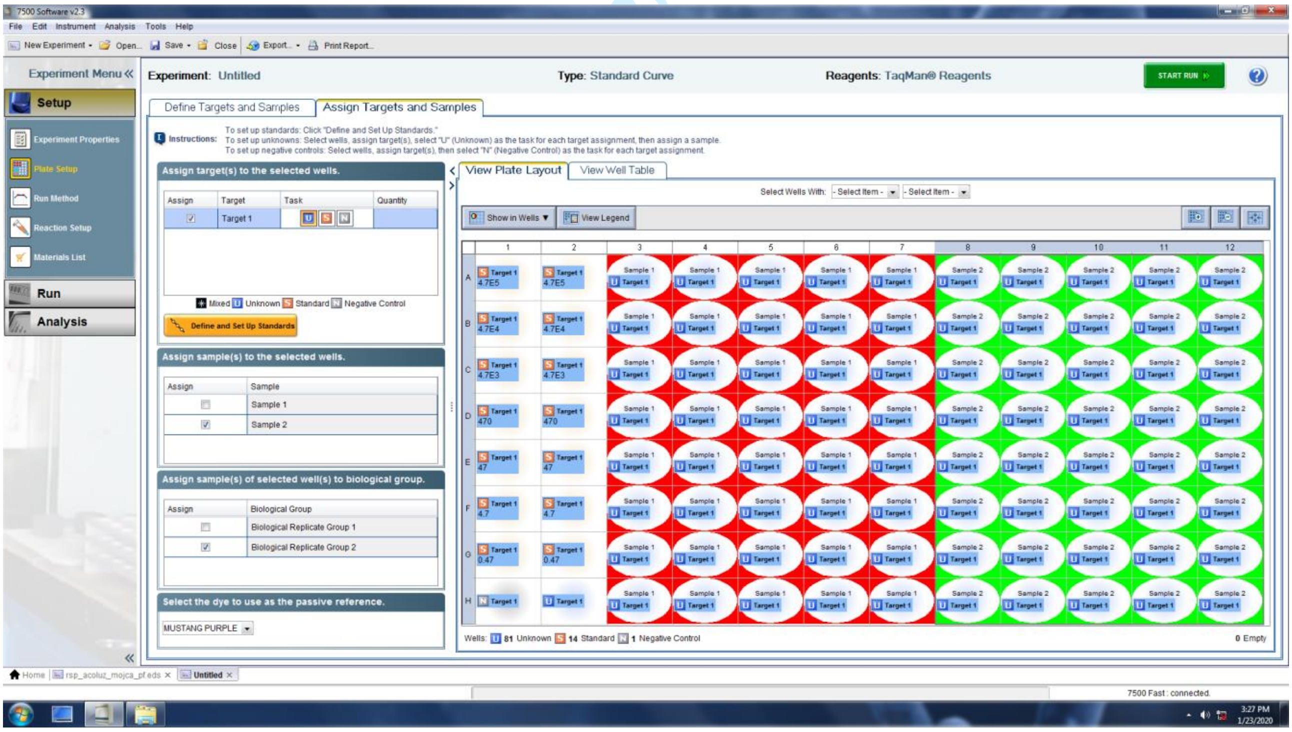

For peer review only - http://bmjopen.bmj.com/site/about/guidelines.xhtml

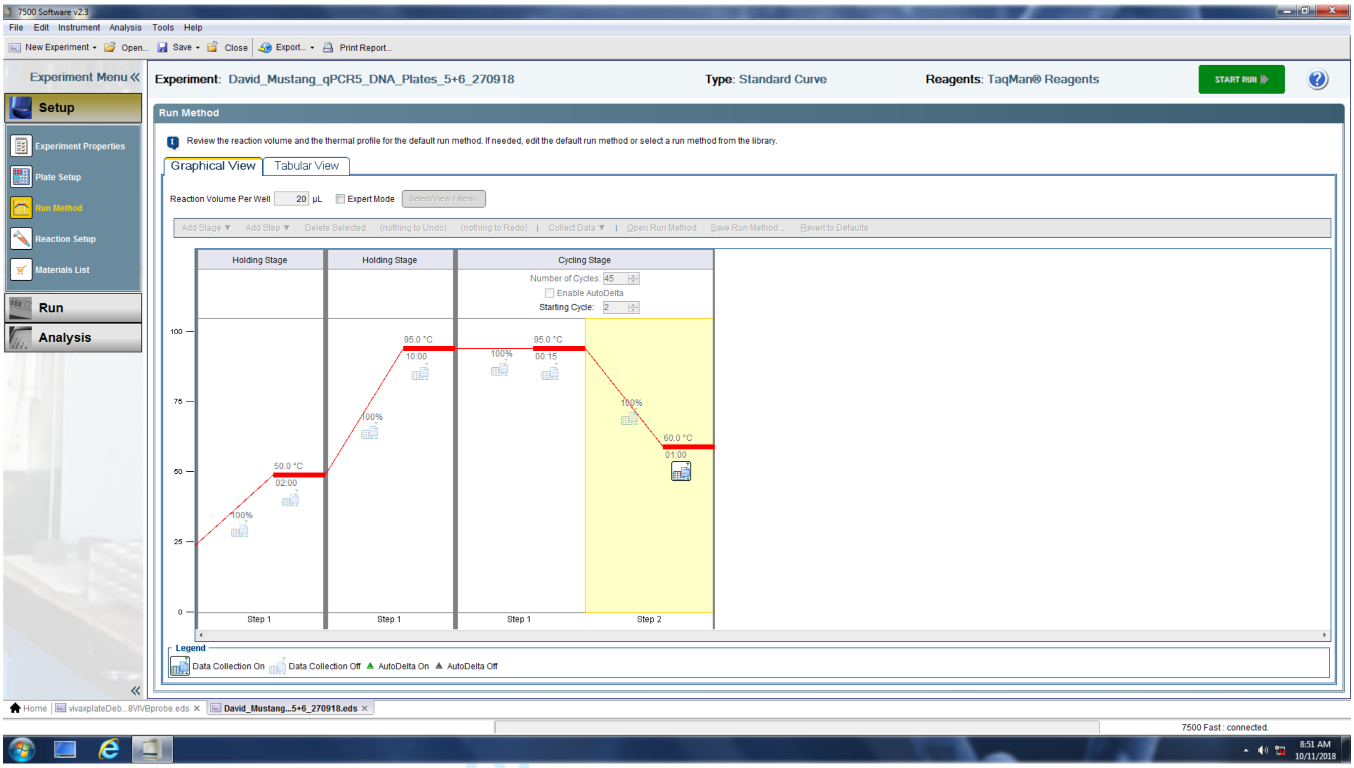

15. Select threshold and baseline value based on the Standards output and investigate the results quality using output plots show below.

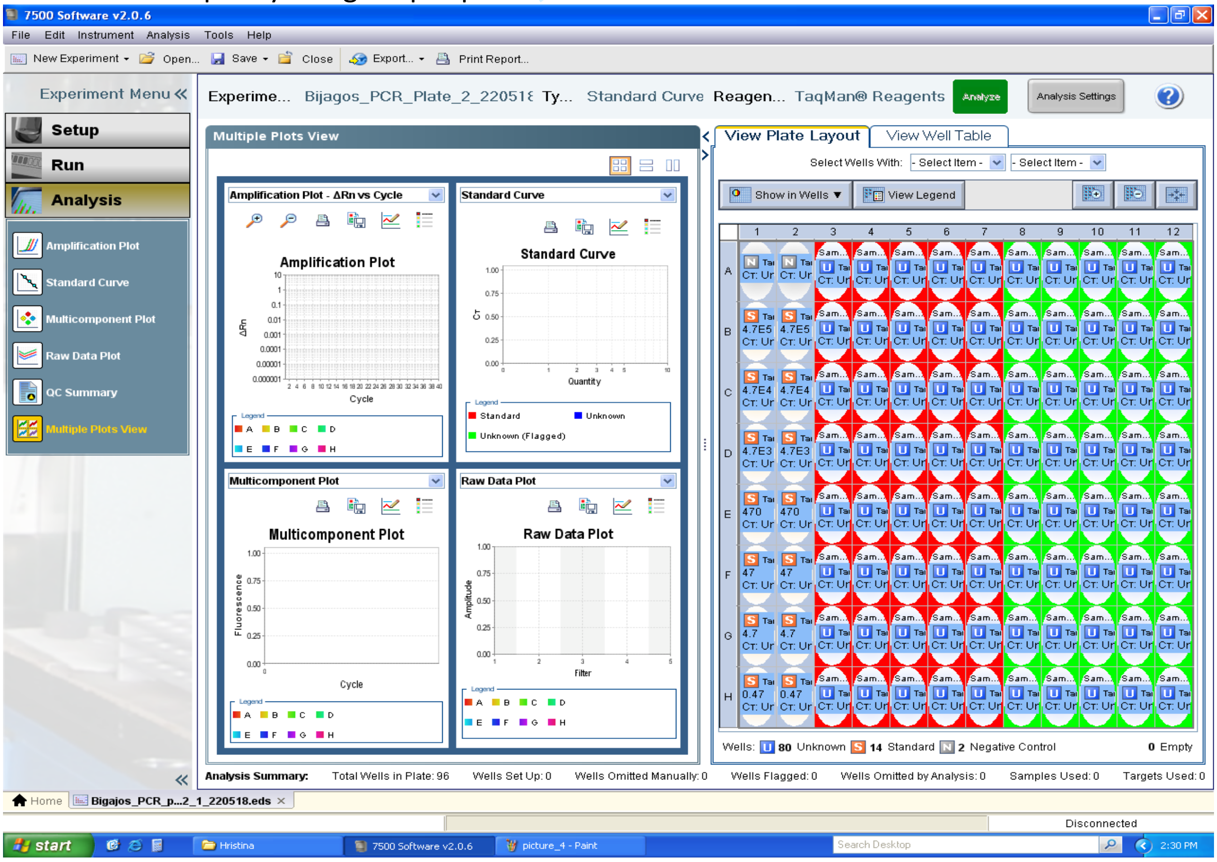

For peer review only - http://bmjopen.bmj.com/site/about/guidelines.xhtml

Membership of Trial Oversight Bodies

MATAMAL Clinical Trial

All appointments have been approved, and made in line with the guidelines issued by, the trial funders and independent ethics review boards. All bodies are independent of the sponsor. All members declare that they have no competing interests.

Trial Steering Committee

- Steve Lindsay (***Independent Chair***)
  Professor of Public Health Entomology
  Durham University, Durham, UK
- Menno Smit
  Honorary Associate Professor
  LSTM, Liverpool, UK
- Thom Eisele
  Director, Applied Malaria Research and Evaluation
  Tulane University School of Public Health and Tropical Medicine, USA
- Oumar Gaye
  Professor of Medical Parasitology
  University Cheikh Anta Diop, Dakar, Senegal
- Anna Last (***Principal Investigator***)
  Associate Professor in Infectious Diseases
  LSHTM, London, UK
- David Mabey
  Professor of Communicable Diseases
  LSHTM, London, UK
- Umberto D'Alessandro
  Director
  MRC The Gambia @ LSHTM, Fajara, The Gambia
- Amabelia Rodrigues (***Local Principal Investigator***)
  Director of Research
  Bandim Health Project, Bissau, Guinea-Bissau
- John Bradley (***Trial Statistician***)
  Assistant Professor of Medical Statistics and Epidemiology
  LSHTM, London, UK
- Harry Hutchins (***Clinical Trial Manager***)
  Clinical Research Fellow
  LSHTM, London, UK

Trial Steering Committee Observers

- Immo Kleinschmidt

For peer review only

Professor of Epidemiology
LSHTM, London, UK

- Brian Greenwood
  Professor of Clinical Tropical Medicine
  LSHTM, London, UK
- MRC appointed observer (Jo Mulligan, FCDO)
- LSHTM appointed observer (Sponsor/Host)

Data Safety Monitoring Board

- Chi Eziefula
  Clinical Epidemiologist
  Brighton and Sussex Medical School, Brighton, UK
- Johan Ursing
  Clinical Epidemiologist
  Karolinska Institute, Sweden
- Mahamadou Ali Thera (***Chair***)
  Malariologist
  University of Sciences Techniques and Technology, Bamako, Mali
- Jennifer Smith
  Epidemiologist and Biostatistician
  University of California, San Francisco, USA

For peer review only

Reporting checklist for protocol of a clinical trial.

Based on the SPIRIT guidelines.

Instructions to authors

Complete this checklist by entering the page numbers from your manuscript where readers will find each of the items listed below.

Your article may not currently address all the items on the checklist. Please modify your text to include the missing information. If you are certain that an item does not apply, please write "n/a" and provide a short explanation.

Upload your completed checklist as an extra file when you submit to a journal.

In your methods section, say that you used the SPIRIT reporting guidelines, and cite them as:

Chan A-W, Tetzlaff JM, Gøtzsche PC, Altman DG, Mann H, Berlin J, Dickersin K, Hróbjartsson A, Schulz KF, Parulekar WR, Krleža-Jerić K, Laupacis A, Moher D. SPIRIT 2013 Explanation and Elaboration: Guidance for protocols of clinical trials. BMJ. 2013;346:e7586

For peer review only

		Reporting Item	Page Number
**Administrative information**			
Title	#1	Descriptive title identifying the study design, population, interventions, and, if applicable, trial acronym	1

Trial registration	#2a	Trial identifier and registry name. If not yet registered, name of intended registry	2
Trial registration: data set	#2b	All items from the World Health Organization Trial Registration Data Set	N/A, Registry
Protocol version	#3	Date and version identifier	2
Funding	#4	Sources and types of financial, material, and other support	12
Roles and responsibilities: contributorship	#5a	Names, affiliations, and roles of protocol contributors	1, 12
Roles and responsibilities: sponsor contact information	#5b	Name and contact information for the trial sponsor	12
Roles and responsibilities: sponsor and funder	#5c	Role of study sponsor and funders, if any, in study design; collection, management, analysis, and interpretation of data; writing of the report; and the decision to submit the report for publication, including whether they will have ultimate authority over any of these activities	12
Roles and responsibilities: committees	#5d	Composition, roles, and responsibilities of the coordinating centre, steering committee, endpoint adjudication committee, data management team, and	11, supp.

For peer review only

		other individuals or groups overseeing the trial, if applicable (see Item 21a for data monitoring committee)	
**Introduction**			
Background and rationale	#6a	Description of research question and justification for undertaking the trial, including summary of relevant studies (published and unpublished) examining benefits and harms for each intervention	3-4
Background and rationale: choice of comparators	#6b	Explanation for choice of comparators	3-4
Objectives	#7	Specific objectives or hypotheses	5
Trial design	#8	Description of trial design including type of trial (eg, parallel group, crossover, factorial, single group), allocation ratio, and framework (eg, superiority, equivalence, non-inferiority, exploratory)	5
**Methods: Participants, interventions, and outcomes**			
Study setting	#9	Description of study settings (eg, community clinic, academic hospital) and list of countries where data will be collected. Reference to where list of study sites can be obtained	5-6

For peer review only

Eligibility criteria	#10	Inclusion and exclusion criteria for participants. If applicable, eligibility criteria for study centres and individuals who will perform the interventions (eg, surgeons, psychotherapists)	5
Interventions: description	#11a	Interventions for each group with sufficient detail to allow replication, including how and when they will be administered	6
Interventions: modifications	#11b	Criteria for discontinuing or modifying allocated interventions for a given trial participant (eg, drug dose change in response to harms, participant request, or improving / worsening disease)	6
Interventions: adherance	#11c	Strategies to improve adherence to intervention protocols, and any procedures for monitoring adherence (eg, drug tablet return; laboratory tests)	6
Interventions: concomitant care	#11d	Relevant concomitant care and interventions that are permitted or prohibited during the trial	6
Outcomes	#12	Primary, secondary, and other outcomes, including the specific measurement variable (eg, systolic blood pressure), analysis metric (eg, change from baseline, final value, time to event), method of aggregation (eg, median, proportion), and time point for each outcome. Explanation of the clinical relevance of chosen efficacy and harm outcomes is strongly recommended	6-9, Fig 1

For peer review only

Participant timeline	#13	Time schedule of enrolment, interventions (including any run-ins and washouts), assessments, and visits for participants. A schematic diagram is highly recommended (see Figure)	6-11, Fig 1
Sample size	#14	Estimated number of participants needed to achieve study objectives and how it was determined, including clinical and statistical assumptions supporting any sample size calculations	7
Recruitment	#15	Strategies for achieving adequate participant enrolment to reach target sample size	6
**Methods: Assignment of interventions (for controlled trials)**			
Allocation: sequence generation	#16a	Method of generating the allocation sequence (eg, computer-generated random numbers), and list of any factors for stratification. To reduce predictability of a random sequence, details of any planned restriction (eg, blocking) should be provided in a separate document that is unavailable to those who enrol participants or assign interventions	5
Allocation concealment mechanism	#16b	Mechanism of implementing the allocation sequence (eg, central telephone; sequentially numbered, opaque,	5-6

For peer review only

		sealed envelopes), describing any steps to conceal the sequence until interventions are assigned	
Allocation: implementation	#16c	Who will generate the allocation sequence, who will enrol participants, and who will assign participants to interventions	6
Blinding (masking)	#17a	Who will be blinded after assignment to interventions (eg, trial participants, care providers, outcome assessors, data analysts), and how	6
Blinding (masking): emergency unblinding	#17b	If blinded, circumstances under which unblinding is permissible, and procedure for revealing a participant's allocated intervention during the trial	6
**Methods: Data collection, management, and analysis**			
Data collection plan	#18a	Plans for assessment and collection of outcome, baseline, and other trial data, including any related processes to promote data quality (eg, duplicate measurements, training of assessors) and a description of study instruments (eg, questionnaires, laboratory tests) along with their reliability and validity, if known. Reference to where data collection forms can be found, if not in the protocol	Suppl, 6-11

For peer review only

Data collection plan: retention	#18b	Plans to promote participant retention and complete follow-up, including list of any outcome data to be collected for participants who discontinue or deviate from intervention protocols	6
Data management	#19	Plans for data entry, coding, security, and storage, including any related processes to promote data quality (eg, double data entry; range checks for data values). Reference to where details of data management procedures can be found, if not in the protocol	9, 11
Statistics: outcomes	#20a	Statistical methods for analysing primary and secondary outcomes. Reference to where other details of the statistical analysis plan can be found, if not in the protocol	9-11
Statistics: additional analyses	#20b	Methods for any additional analyses (eg, subgroup and adjusted analyses)	9-11
Statistics: analysis population and missing data	#20c	Definition of analysis population relating to protocol non-adherence (eg, as randomised analysis), and any statistical methods to handle missing data (eg, multiple imputation)	9-11
**Methods: Monitoring**			
Data monitoring: formal committee	#21a	Composition of data monitoring committee (DMC); summary of its role and reporting structure; statement of whether it is independent from the sponsor and competing interests; and reference to where further	11, suppl.

For peer review only

		details about its charter can be found, if not in the protocol. Alternatively, an explanation of why a DMC is not needed	
Data monitoring: interim analysis	#21b	Description of any interim analyses and stopping guidelines, including who will have access to these interim results and make the final decision to terminate the trial	9-11
Harms	#22	Plans for collecting, assessing, reporting, and managing solicited and spontaneously reported adverse events and other unintended effects of trial interventions or trial conduct	9-11
Auditing	#23	Frequency and procedures for auditing trial conduct, if any, and whether the process will be independent from investigators and the sponsor	12
**Ethics and dissemination**			
Research ethics approval	#24	Plans for seeking research ethics committee / institutional review board (REC / IRB) approval	11
Protocol amendments	#25	Plans for communicating important protocol modifications (eg, changes to eligibility criteria, outcomes, analyses) to relevant parties (eg, investigators, REC / IRBs, trial participants, trial registries, journals, regulators)	11

For peer review only

Consent or assent	#26a	Who will obtain informed consent or assent from potential trial participants or authorised surrogates, and how (see Item 32)	5-6
Consent or assent: ancillary studies	#26b	Additional consent provisions for collection and use of participant data and biological specimens in ancillary studies, if applicable	5-6
Confidentiality	#27	How personal information about potential and enrolled participants will be collected, shared, and maintained in order to protect confidentiality before, during, and after the trial	11
Declaration of interests	#28	Financial and other competing interests for principal investigators for the overall trial and each study site	12
Data access	#29	Statement of who will have access to the final trial dataset, and disclosure of contractual agreements that limit such access for investigators	11
Ancillary and post trial care	#30	Provisions, if any, for ancillary and post-trial care, and for compensation to those who suffer harm from trial participation	N/A
Dissemination policy: trial results	#31a	Plans for investigators and sponsor to communicate trial results to participants, healthcare professionals, the public, and other relevant groups (eg, via publication, reporting in results databases, or other data sharing arrangements), including any publication restrictions	11-12

For peer review only

Dissemination policy: authorship	#31b	Authorship eligibility guidelines and any intended use of professional writers	12
Dissemination policy: reproducible research	#31c	Plans, if any, for granting public access to the full protocol, participant-level dataset, and statistical code	11

Appendices

Informed consent materials	#32	Model consent form and other related documentation given to participants and authorised surrogates	Suppl.
Biological specimens	#33	Plans for collection, laboratory evaluation, and storage of biological specimens for genetic or molecular analysis in the current trial and for future use in ancillary studies, if applicable	Suppl.

The SPIRIT Explanation and Elaboration paper is distributed under the terms of the Creative Commons Attribution License CC-BY-NC. This checklist was completed on 28. January 2023 using https://www.goodreports.org/, a tool made by the EQUATOR Network in collaboration with Penelope.ai

For peer review only
